# Predicting the Solubility of Amino Acids and Peptides with the SAFT-γ Mie Approach: Neutral and Charged Models

**DOI:** 10.1021/acs.iecr.4c02995

**Published:** 2024-11-11

**Authors:** Ahmed Alyazidi, Shubhani Paliwal, Felipe A. Perdomo, Amy Mead, Mingxia Guo, Jerry Y. Y. Heng, Thomas Bernet, Andrew J. Haslam, Claire S. Adjiman, George Jackson, Amparo Galindo

**Affiliations:** † Department of Chemical Engineering, Institute for Molecular Science and Engineering, and Sargent Centre for Process Systems Engineering, Imperial College London, South Kensington Campus, London SW7 2AZ, United Kingdom

## Abstract

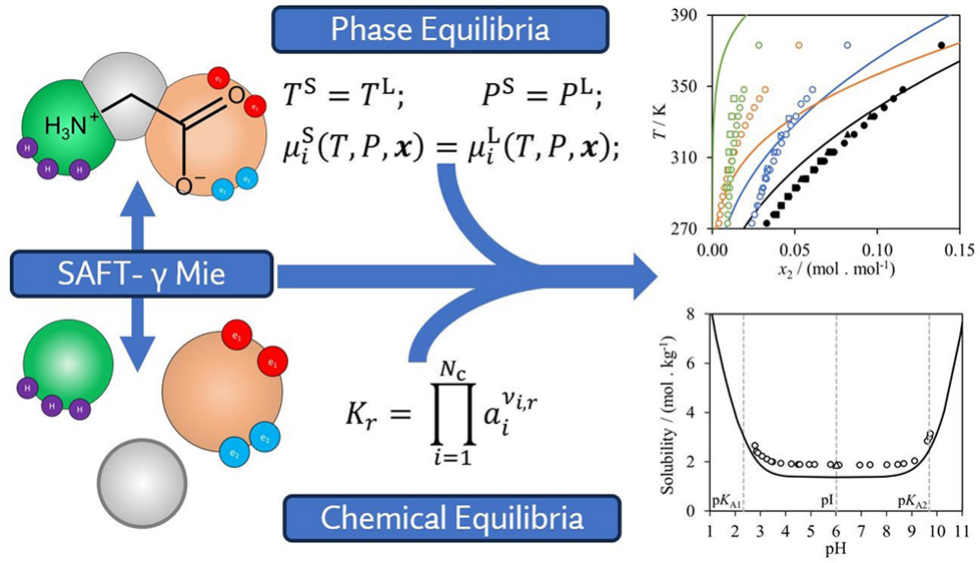

Modeling approaches that can be used to predict accurately the solubility of amino acids and peptides are of interest for the design of new pharmaceutical processes and in the development of new peptide-based therapeutics. We investigate the capability of the SAFT-γ Mie group-contribution approach to predict the aqueous and alcohol solubility of glycine, alananine, valine, leucine, and serine and of di- and tripeptides containing these amino acids. New SAFT-γ Mie group interactions are characterized using experimental thermodynamic and phase-equilibrium data of compounds and mixtures that contain groups relevant to the amino acids and peptides, but no solubility data (except for the case of glycine). Once all the group interaction parameters are developed, predictive solid–liquid solubility calculations are carried out. Neutral and charged models are considered to account explicitly for the zwitterionic nature of the molecules in aqueous solution, and the solubility of the solution is presented as a function of pH. A detailed discussion of the molecular models and Helmholtz free-energy expressions used to represent the ionic and zwitterionic forms of the amino acids, together with their speciation in solution is also provided. Overall, very good agreement with available data is shown, with an absolute average deviation (AAD) in mole fraction of 0.0038 over 283 solubility data points for the amino acids studied and an AAD in mole fraction of 0.02128 over 141 peptide-solubility points when the systems are studied at their isoelectric point and neutral models are used. The solubility as a function of pH for a range of temperatures is also predicted accurately when charged models are incorporated. These results confirm the predictive accuracy of the SAFT-γ Mie method and pave the way for future studies involving larger peptides.

## Introduction

Peptides are gaining popularity as active pharmaceutical ingredients (APIs) for the treatment of illnesses such as diabetes^1^ and some cancers.^2,3^ Of special interest are those that mimic natural hormones or can disrupt protein–protein interactions,^4^ while also exhibiting very low toxicity and good *in vivo* stability.^5^ As new peptide-based therapeutics are proposed, a good understanding of the thermodynamic properties of these molecules and their mixtures is crucial for product development and manufacturing. In particular, the solubility of APIs in pure and mixed solvents is especially important as it determines the bioavailability of the drug and the optimal solvent choice in the synthesis and purification stages. With the number of peptide-based APIs in discovery increasing year by year, it can be expensive and time-consuming to determine experimentally the solubility of each candidate under broad thermodynamic conditions and diverse solvent media. In this context, inexpensive computational tools that can be used to model and predict accurately the solid–liquid equilibrium (the solubility) of APIs in general and peptides in particular are becoming increasingly important in the development and design of pharmaceutical products.

Since peptides are oligomers made from a finite pool of amino-acid residues, a good understanding of the physicochemical properties of amino-acid mixtures serves as a key to the modeling of peptide mixtures. In 1930, Harris and Birch^6^ demonstrated that amino acids exist as zwitterions in aqueous solution, and since then many authors^7,8^ have assumed that they transform from their neutral form to the zwitterionic form after dissolution. However, more recent X-ray diffraction measurements have confirmed that amino acids exist in zwitterionic form even in the solid phase.^9−11^ In the case of peptides and proteins, the state of charge is not yet clear.^12−16^

Due to their zwitterionic nature, amino acids are generally soluble in water and insoluble in nonpolar organic solvents, although the nature and size of side chains play an important role in the extent of their solubility. For example, amino acids with hydrophilic, i.e., charged and polar, side chains (e.g., lysine and serine) tend to have a higher aqueous solubility than those with hydrophobic side chains (e.g., leucine and valine). Peptides exhibit varying solubilities depending on their size, the nature of any side chains, the precise sequence of amino-acid residues in their backbone, and the structure(s) they exhibit in the solid state. These factors contribute to other important phenomena, such as the formation of intramolecular hydrogen bonds and aggregate formation that further impact their phase-equilibrium behavior.

There have been surprisingly few theoretical attempts to model amino-acid solubility. One of the earliest is due to Kirkwood^17^ in 1934, who used a statistical-mechanical approach treating the amino acid as a sphere with discrete point charges and the solvent as a dielectric continuum to study the effect of the dielectric constant of the solvent on the solubility of amino acids. Kirkwood neglected the dipole–dipole interactions between the amino acid molecules; thus, his theory applied strictly only to solutions at infinite dilution. In the same year, Cohn et al.^18^ performed a systematic experimental investigation of the solubility of amino acids in water, alcohols, and alcohol–water mixtures, in which they found that amino acids behave in a similar manner to strong electrolytes, i.e., they are soluble in water but highly insoluble in alcohols and exhibit high solid densities, reflecting the charged nature of the molecules. Cohn et al. reported that “the activity coefficients of the larger amino acids deviate far more than those of glycine from any relation proportional to change in the mole fraction of alcohol or the dielectric constant of the solutions”. This partly explains why Kirkwood’s^17^ theoretical treatment was accurate only in the case of glycine, and only at very low concentrations.

Interest in modeling amino-acid and peptide systems was revived in the late 1980s and 1990s through the adoption of semiempirical models. Chen et al.^19^ combined the nonrandom two-liquid (NRTL) equation with a Pitzer–Debye–Hückel term^20^ that varied inversely with the solvent dielectric constant and, unlike Kirkwood, treated the coefficient of the electrostatic term as an adjustable parameter; this allows one to capture the varying behavior of different solvents. Orella and Kirwan^21^ combined an excess-solubility approach with the three-suffix Margules, NRTL, and Wilson activity-coefficient models to correlate solubility data of amino acids in mixed water + alcohol solvents. They used published solvent–solvent parameters and correlated the solute–solvent parameters using solubility data. They reported that out of the three models, Wilson’s, yielded the best agreement with experiments, although, as concluded later by Ferreira et al.,^22^ the global quality of results in Orella and Kirwan’s work seems to contradict this conclusion.

Gude et al.^23^ combined the excess-solubility approach with a simple activity-coefficient model comprising a combinatorial term based on the Flory–Huggins theory and a Margules residual term, and reported good correlations of the solubility and partition coefficient of amino acids and small peptides in mixed water + alcohol solvents. van Berlo et al.^24^ extended the use of the model of Gude et al. to correlate the solubility of glycine in the ternary solvent water + ethanol + 1-butanol. They used the vapor−liquid equilibria (VLE) of the water + ethanol + 1-butanol solute-free system, along with single-solvent solubility data of glycine to develop a one-parameter excess Gibbs model to predict the solubility and partition coefficient of glycine in the ternary solvent system at the same temperature. Rudolph et al.^25^ used the same model to study the solubility and partition coefficients of amoxicillin, ampicillin, and their precursors. They found, however, that they could not accurately reproduce the increasing relative solubility of the molecules in the aqueous phase observed for an increasing 1-butanol concentration. To capture the correct aqueous solubility behavior, they replaced the combinatorial term in Gude et al.’s model with that from the universal quasichemical activity-coefficient (UNIQUAC) model to account for the size and shape differences of the molecules. Unfortunately, neither model allows one to describe simultaneously the solubilities and partitioning accurately. The model of Ferreira et al.,^22^ in which the excess solubility approach is combined with the NRTL equation, showed an improvement in correlating the solubility of amino acids and small peptides in mixed solvents, as compared to the similar models of Orella and Kirwan^21^ and Gude et al.,^23^ while using the same number of adjustable parameters.

In addition to the use of local-composition and group-contribution activity-coefficient models, molecular-based models have been increasingly adopted since the 1990s. Khoshkbarchi and Vera^26^ developed a simplified perturbed hard-sphere model to correlate the activity coefficients and solubilities of amino acids in water, both at the isoelectric point (p*I*) and at varying pH. A Lennard-Jones (LJ) potential was used to model the dispersion forces, and a Keesom term was introduced to account for the dipole–dipole interactions between amino acids. The solvent was treated as a dielectric continuum. The dipole moments of the amino acids were calculated using a quantum-mechanical approach, whereas the LJ potential parameters were adjusted using activity-coefficient data. The enthalpy of fusion and melting temperature were adjusted using the amino acid solubility curves, and in order to model the pH-dependent solubility, experimental dissociation equilibrium constants were used.

In later work, the authors extended their treatment to study how the presence of salts in solution affects the solubility of amino acids^27,28^ and to model the solubility of a mixture of two amino acids in aqueous solutions.^28^ These models marked a significant improvement in the description of the physical behavior of amino acid solutions, especially in accounting for the large dipole moments of the amino acids, although the experimental solubility data required for adjusting the many model parameters are often scarce. It is also important to note that a key shortcoming of these approaches in modeling pH-dependent solubility is that they account for the speciation of the amino acids solely through the concentration of protons in the system. The activities of the amino-acid cation and anion are accounted for only as mole fractions.^26^ This assumption applies only to the system at or close to the isoelectric point and cannot be extrapolated to other pH values.

Fuchs et al.^29^ have used the PC-SAFT^30^ version of the statistical associating fluid theory (SAFT)^31,32^ to model the solubility of glycine, dl-alanine, and dl-methionine at their p*I* in pure water and alcohols and in mixed water+alcohol mixtures. Pure-component model parameters were adjusted using vapor-pressure and liquid-density data of the amino acid aqueous solutions, and an additional solute–solvent interaction parameter was adjusted using solubility data. Moreover, the enthalpy of fusion and melting temperature were also treated as adjustable parameters rather than using experimental values as had been the case in previous work; these solid-state properties were first calculated using the group-contribution method of Marrero and Gani^33^ and then allowed to vary within the average deviation reported for the method (16%) to provide the best description of the experimental solubility data. The solubility in mixed-solvent systems was then predicted to be in fair agreement with experiment. The same model was also used to predict the solubility of the amino acids at variable pH, in good agreement with experimental data, although the influence of the different ionized forms of the amino acid was neglected (much as in ref (26)), with speciation accounted for solely through the concentration of protons. By adoption of this strategy, the need to model the electrostatic interactions between amino acid species is circumvented.

Cameretti and Sadowski^34^ also used the PC-SAFT equation of state (EoS) to correlate the density and vapor pressure of aqueous solutions of glycine, alanine, serine, proline, and valine, treating the enthalpy of fusion and melting temperature as adjustable parameters. The same model parameters were then used to model the properties of peptides with an additional adjustment of the segment-number parameter. Ferreira et al.^35^ later employed a different strategy, treating amino acids as nonassociating, and using only three component-specific PC-SAFT parameters; these were obtained by adjustment using liquid densities, activity and osmotic coefficients, vapor pressures, and water activities of unsaturated aqueous amino-acid solutions. While neglecting association interactions led to a decrease in the number of model parameters, a binary solute–solvent parameter was needed to yield acceptable agreement with experiment. Additionally, the melting properties of the amino acids were adjusted by using aqueous solubility data. Unfortunately, the resulting prediction of the solubility in pure alcohols was unsatisfactory. In both works, the authors treated the amino acids and peptides without considering their speciation.

Grosse Daldrup et al.^36,37^ used the PC-SAFT EoS to model the mixed-solute solubility in water of amino acids of similar and of differing p*I*s, at variable pH, and Held et al.^38^ modeled the density, vapor-pressure depression, activity coefficient, and solubility of aqueous solutions of an extensive list of amino acids. The model parameters in these studies were adjusted using experimental liquid-density and activity-coefficient data of aqueous solutions with the melting properties treated as adjustable parameters. These studies capture the dissociation equilibria associated with changes in pH but do not incorporate a charge in any of the species, including the amino-acid cation and anion. Charged PC-SAFT models have been used by Wysoczanska et al.,^39^ who studied the density, solubility, and partition coefficients of dinitrophenylated amino acids in aqueous two-phase systems, and by Aliyeva et al.^40^ who studied the impact of the addition of salts on the solubility of aromatic and dicarboxylic amino acids using the ePC-SAFT approach.

An important challenge in modeling the solubility of amino acids and peptides remains the scarcity of accurate and reliable solubility data, which are essential for model validation, and melting-property data, which are required as input in the thermodynamic modeling of the solubility. Additionally, despite ongoing research efforts, there remains a notable absence of fully predictive models capable of describing the solubility across a wide range of conditions. Furthermore, existing models often fail to account for the presence and nonideality of the cationic and anionic species of amino acids, which significantly impact the solubility at pH values away from the isoelectric point. In the current work, we develop a predictive framework to calculate the solid–liquid equilibria (SLE) of amino acids in water and alkanols, and their mixtures, including their dependence on pH. We use the SAFT-γ Mie group-contribution EoS,^41−44^ in which molecules are treated as heteronuclear chains of fused spherical segments. These segments represent the functional groups comprising each molecule and interact with each other via Mie potentials of variable range, and hydrogen bonding between some of the groups is modeled through the interaction of association sites embedded in the segments. The approach has been used to model the thermodynamic behavior and properties of several complex mixtures.^41,42,45−47^ A review of studies applying the SAFT-γ Mie approach together with a summary of the groups and interactions that have been parametrized within our research group can be found in reference (48). In particular, the approach has been shown to be accurate for the prediction of the solubility of pharmaceutical compounds,^47,49^ as well as the properties of electrolyte solutions, and specifically, those containing charged organic molecules,^50−52^ and weak electrolytes.^53^ It has also been used to model the solubility of ionizable active pharmaceutical ingredients as a function of pH.^47^

In our current study, the amino acids are treated using standard (neutral) groups first. In the vicinity of the isoelectric point, amino acids exist primarily in one overall neutral (zwitterionic) form; treating them without explicit charge interactions simplifies the modeling by reducing the number of parameters needed. Later, charged interactions are explicitly accounted for by using charged groups to model the different ionized species of the amino acid which are present as the pH varies. In both cases, as is customarily done in the SAFT-γ Mie approach, dipole–dipole interactions are accounted for effectively through the variable-range Mie potential as well as through the embedded association sites.^54^ The chemical-equilibrium equations are included to determine the amounts of different species present at any given pH. To demonstrate the predictive capability of the model, we do not use solubility data of the amino acids and peptides under consideration in the optimization of the SAFT-γ Mie group parameters. Moreover, we use the melting temperatures and enthalpies of fusion reported by Do et al.^55,56^ without further adjustment.

The remainder of this article is set out as follows: In the section “SAFT-γ Mie Equation of State”, we describe the SAFT-γ Mie theory and provide details of the main Helmholtz free-energy terms, with special attention to the electrostatic contributions. In the section “Amino Acids and Peptides at the Isoelectric Point: Uncharged Models”, the prediction of the solubility of amino acid solutions using neutral groups is discussed. In the section “Solubility of Amino Acids as a Function of pH: Charged Models” the impact of pH changes, including the modeling of the speciation of amino acid zwitterions into the cationic and ionic amino acid forms by incorporating charged groups, are presented; concluding remarks are given in the “Conclusions”.

## SAFT-γ Mie Equation of State

In the SAFT-γ Mie group-contribution (GC) framework, molecules are modeled as heteronuclear chains of fused spherical segments with association sites embedded to mediate hydrogen bonding or directional interactions. Any compound *i* (neutral or ionic) is represented by its different constituent groups, with the number of occurrences of a group of type *k* denoted by ν_*k*,*i*_. Each functional group consists of ν_k_^*^ identical fused spherical segments.

The thermodynamic properties of a fluid mixture are obtained through derivatives of the Helmholtz free energy, which is written as a sum of contributions:^41,42,50^
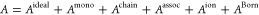
1where *A*^ideal^ corresponds to the free energy of an ideal gas mixture; *A*^mono^ accounts for the interactions among monomer segments, described using Mie potentials; *A*^chain^ accounts for the free energy due to chain formation; *A*^assoc^ accounts for association mediated through short-range directional forces; *A*^ion^ accounts for the Coulombic ion–ion interactions; and *A*^Born^ accounts for ion–solvent electrostatic interactions. Only the first four terms are used when modeling neutral systems. The last two terms are included when charged groups are present in the system.

### The Helmholtz Free Energy of Neutral Systems

The ideal term is given by^57^
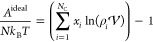
2where *N* is the total number of molecules in the system, *k*_B_ is the Boltzmann constant, *T* is the absolute temperature, *N*_C_ is the total number of components in the mixture, *x*_*i*_ is the mole fraction of component *i*, ρ_*i*_ = *N*_*i*_/*V* is the number density (*V* is the total volume of the system and *N*_*i*_ is the number of molecules of component *i*), and  is taken to represent the thermal de Broglie volume, which implicitly accounts not only for the translational contribution to the kinetic energy but also those from rotations and vibrations of the molecules.

The monomer term accounts for repulsion and dispersion interactions between monomer segments and is expressed using a Barker–Henderson^58,59^ high-temperature perturbation expansion up to third-order:^54^
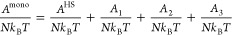
3where *A*^HS^ is the hard-sphere reference free energy, and *A*_1_, *A*_2_, and *A*_3_ are the first, second, and third-order terms of the expansion. For details of these individual terms see, e.g., ref (48).

The chain term corresponds to the change in free energy due to the connectivity of monomer segments forming the molecules of the system. This term is formulated using the TPT1 expression of Wertheim^60,61^ as
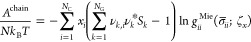
4where *N*_*G*_ is the total number of group types in the mixture, *S*_*k*_ is the shape factor, which describes the contribution of group *k* to the overall Helmholtz free energy of the molecule in terms of a noninteger number of segments, *g*_*ii*_^Mie^(σ̅_*ii*_; ζ_*x*_) is the radial distribution function evaluated at the average molecular segment contact diameter σ̅_*ii*_ of component *i*, in a hypothetical fluid of packing fraction ζ_*x*_. A more detailed description of this term can be found in the original SAFT-γ Mie paper;^41^ note, however, that there is a typographical error (misplaced bracket) in the expression of the chain term (eq (46) in ref (41)), which is presented correctly in eq 4 here.

The association term accounts for the contribution to the free energy due to the association between molecules via short-range directional interactions and is expressed, based on the TPT1 perturbation theory of Wertheim,^60^ as
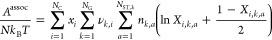
5where *N*_ST,k_ is the total number of site types, *n*_*k*,*a*_ the number of association sites of type *a* for group *k*, and *X*_*i*,*k*,*a*_ the fraction of molecules of component *i* that are not bonded at site *a* on group *k*.

### Electrostatic Contributions to the SAFT-γ Mie Helmholtz Free Energy

In the case of mixtures containing ionic species, the electrostatic interactions between ions and those between ions and neutral solvent molecules are accounted for with the inclusion of the ion and Born terms in the expression of the Helmholtz free energy of the mixture (eq 1). In the SAFT-γ Mie equation, we use the classic expression of Blum for the solution of the mean spherical approximation (MSA) in a nonrestricted electrolyte primitive model^62,63^ to account for charge–charge Coulombic interactions, and the expression of Born^64^ to incorporate ion–solvent (charge–dipole) interactions, following the SAFT-VR Mie EoS.^65^ We note also that Kournopoulos et al.^66^ have demonstrated the validity of the MSA and Born terms in the modeling of strong electrolytes.

It is important to note that in both the MSA and Born approaches (as well as in the seminal Debye–Hückel model) the ionic particles in the underlying molecular model are assumed to be spherical. The assumption of spherical charged particles in incorporating the ion and Born contributions to the Helmholtz free energy is commonly used in electrolyte equations of state, such as the eSAFT-VR Mie EoS^67^ (in which the Debye–Hückel^68^ term is used instead of the MSA), the electrolyte cubic plus association (eCPA)^69^ (in which a Soave–Redlich–Kwong (SRK)^70^ residual term, an association term,^71^ a Debye–Hückle term,^68^ and a Born term^64^ are combined), and the ePC-SAFT EoS.^72^ In the case of nonspherical ionic species, a decision must be made to reconcile the molecular (nonspherical) model of a charged species with the classical ionic expressions that assume a spherical charged particle.

**Figure 1:**
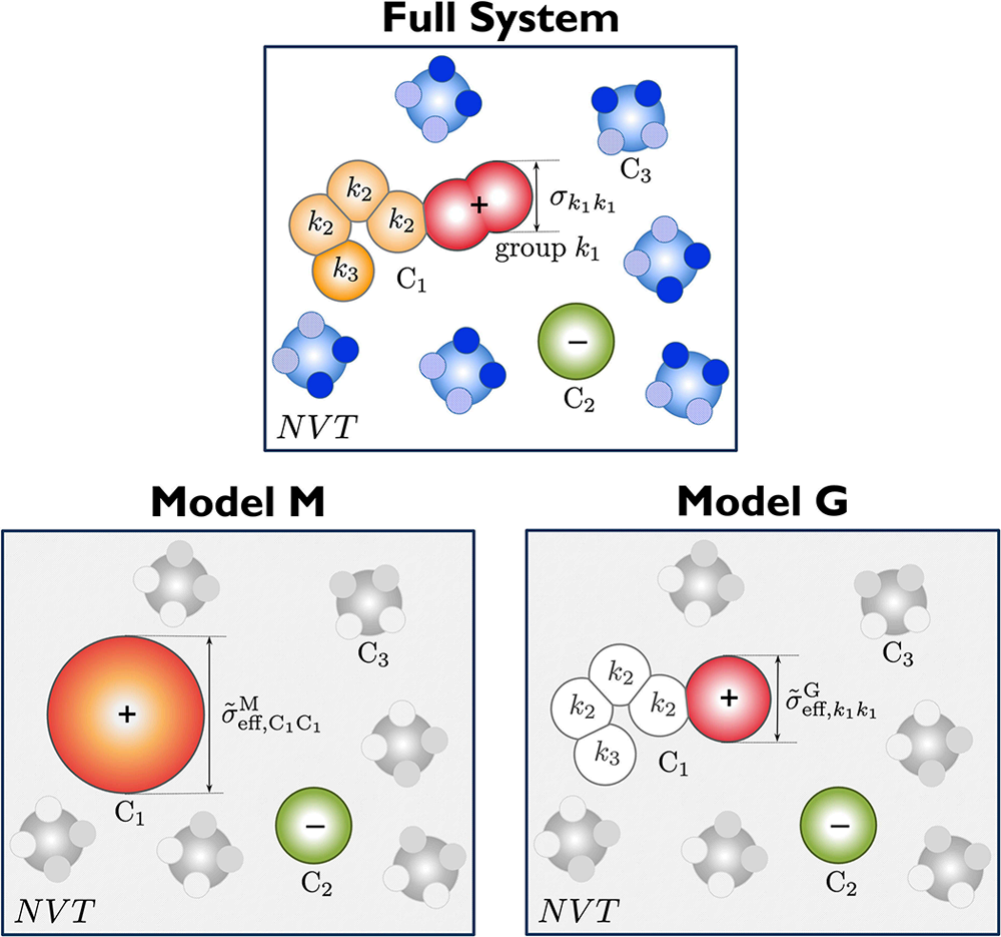
A simplified representation of the models considered within the SAFT-γ Mie approach in the current work. The upper panel depicts an electrolyte comprising a chain-like cation (C_1_) and a spherical anion (C_2_) in water (C_3_). The cation is modeled as a heteronuclear chain of groups including a charged group comprising two identical segments. The bottom panels depict the effective models proposed for the evaluation of the ion and Born contributions of the Helmholtz free energy. Model M refers to “molecular” and Model G refers to “group”.

Here, we discuss two possible mappings^50^ to reconcile the heteronuclear SAFT-γ Mie molecular model with expressions accounting for spherical ionic interactions. These two routes are presented in Figure 1. As an example, consider an electrolyte containing a chain-like cation (component C_1_), a spherical anion (C_2_), and a solvent (C_3_). The cation comprises a charged group *k*_1_ formed by two identical segments ν_*k*_1__^*^ = 2, each of diameter σ_*k*_1_*k*_1__ and Born (solvated) diameter σ_*k*_1_*k*_1__^Born^, which contribute to the overall free energy with a corresponding shape factor *S*_*k*_1__. The group has a positive (central) charge *Z*_*k*_1__. The rest of the cationic species is formed by a heteronuclear chain comprising three groups of type *k*_2_ and one of type *k*_3_. For simplicity, the anion is considered to be spherical here, but the expressions provided below are equally applicable in the case of chain-like anions. The neutral solvent molecules are represented with the standard SAFT representation of water in the figure.

In the first approach, which we refer to as Model M (molecular), the entire ionic chain molecule *i* is mapped onto a single sphere of effective ionic diameter given as
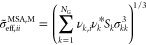
6which is defined to maintain the original molecular volume while mapping the chain onto a sphere. An effective Born diameter given as
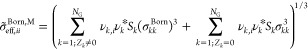
7is also defined. Furthermore, the effective spherical ion carries a central point charge corresponding to the net charge of the original molecule
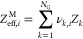
8where *Z*_*k*_ is the charge of group *k*, so that the overall molecular charge density of the ion remains unchanged. Note that in the case where the net charge of *i* is zero, as is the case of a zwitterion, the ion and Born terms *A*^ion^ and *A*^Born^ do not contribute to the free energy of the mixture (they are zero).

In the second approach, which we refer to as Model G (group), only the charged group is mapped to a sphere of effective ionic and Born diameters σ̃_eff,*kk*_^MSA,*G*^ and σ̃_eff,*kk*_^Born,*G*^, given as
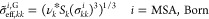
9such that only the charged group is mapped onto a spherical group of equivalent volume. The charge *Z*_*k*_ of the group remains unchanged. The rest of the groups in the ion (e.g., groups *k*_2_ and *k*_3_ in the figure) also remain unchanged and do not contribute to the ionic or Born terms, as they do not carry a charge. In our current work, we adopt Model G as it represents a more physically accurate picture of the ionic molecule within a group-contribution framework. Therefore, we present here the detailed equations corresponding to Model G. (The analogous equations relating to Model M can be found in the Supporting Information.)

The ion term in eq 1 represents the contribution to the free energy due to the electrostatic interactions between charged groups formulated according to the mean spherical approximation (MSA)^62,63^ as
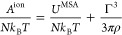
10where *U*^MSA^ is the MSA contribution to the internal energy and Γ is the screening length of the electrostatic forces. Within the SAFT-γ Mie formulation, and implementing Model G as described above, *U*^MSA^ is given by
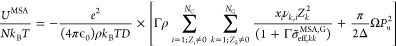
11where *e* = 1.602 × 10^–19^ C is the elementary charge, ϵ_0_ = 8.854 × 10^–12^ C^2^ J^–1^ m^–1^ is the static permittivity in vacuum, and *D* the relative static permittivity. *Z*_*i*_ is the net charge of compound *i*, given by
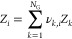
12The constraint *Z*_*i*_ ≠ 0 indicates that the outer sum over components includes only ions with a net charge (i.e., it excludes uncharged molecules and zwitterions) and, similarly, the constraint *Z*_*k*_ ≠ 0 denotes that the sum over groups includes only those that are charged. Δ, given by
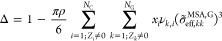
13describes the packing fraction of the ions as a function of the effective ionic diameter σ̃_eff,*kk*_^MSA,*G*^ which is given by eq 9.
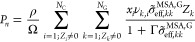
14and
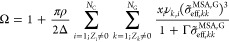
15are coupling parameters that are functions of the ionic parameters and the screening length of the ions. *P*_*n*_ couples the charges of the ions and Ω relates to the packing fractions of the ions. The screening length of the ions Γ is a function of the relative static permittivity *D* and the effective charge *Q*_*k*_ (Γ) of the ions, leading to an implicit formulation through the electric charge of the individual ionic groups *Q*_*k*_:
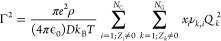
16where the effective charge is related to *Q*_*k*_ and the *P*_*n*_ coupling parameter is expressed as
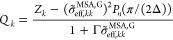
17This concludes the presentation of the ionic term. Expressions for the ion contributions to the chemical potential and pressure, needed when performing phase-equilibrium calculations, are provided in the Supporting Information.

The contribution to the free energy due to ion solvation, *A*^Born^, is incorporated using the classical Born^64^ expression. Accounting for the proposition of Model G, this term is given by
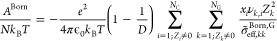
18where the effective Born diameter, σ̃_eff,*kk*_^Born^, is calculated using eq 9 where the Born diameter of the spherical cavity created by each ionic group *k* in the dielectric medium is obtained independently of any other ionic group.

The relative static permittivity is given by^73^
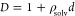
19where ρ_solv_ = *N*_solv_/*V* is the solvent number density of the system, with *N*_solv_ the number of molecules of solvent. In the model proposed here, only a species not containing charged groups (regardless of the net charge) is considered to be a solvent, meaning that any zwitterionic molecule is not a solvent. The variable *d* is calculated as
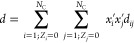
20where *i*, *j* are not zwitterions and *x*_*i*_^′^ and *x*_*j*_^′^ are the salt-free mole fractions of solvents *i* and *j*. The summation is over solvent species only because of the constraints *Z*_*i*_ = 0 and *Z*_*j*_ = 0, with *i* and *j* not being a zwitterion. *d*_*ii*_ is the temperature-dependent contribution to *D* from solvent *i* obtained from
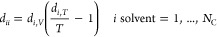
21where *d*_*i*,*V*_ and *d*_*i*,*T*_ are component-specific adjustable parameters. These have been provided for several solvents in previous work.^73^ The unlike *d*_*ij*_ term is obtained as
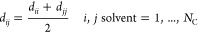
22As can be gleaned from eqs 19–22, the value of the relative static permitivity of the medium is not affected by either the mapping in Model M or in Model G.

Before concluding this section, it is worth highlighting that for Model G, as presented here, in the case of molecules with more than one charged group, each charged group contributes independently to the ionic and Born terms. An exception has been made for the case of zwitterionic molecules, for which the ionic and Born terms are set to zero, accounting for the fact that these are neutral molecules. As we will see in the section “Solubility of Amino Acids as a Function of pH: Charged Models”, these assumptions deliver an accurate description of solutions containing small zwitterions. They may, however, not be appropriate for large zwitterions or polyelectrolytes, which are likely to require other approximations; these will be the subject of future work.

## Amino Acids and Peptides at the Isoelectric Point: Uncharged Models

**Figure 2:**
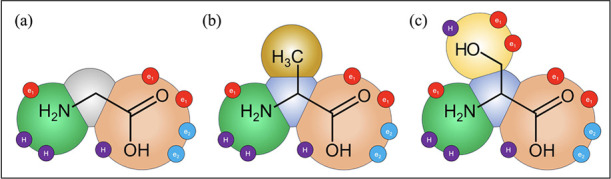
SAFT-γ Mie representation of (a) glycine, (b) alanine, and (c) serine. A heteronuclear model with fused spherical segments is implemented in which short-range association sites are represented with smaller purple (sites of type H), red (type e_1_), and light blue (type e_2_) circles.

In this section, we explore the predictive capability of the SAFT-γ Mie approach for the calculation of solid–liquid equilibria of amino acids and peptides, considering these as neutral molecules, an assumption that is expected to be valid for calculations at the isoelectric point, where the prevalent species in the system is the zwitterion. In this case, we implement a SAFT-γ Mie model where no charged groups are considered, i.e., not only is the amino acid (or peptide) neutral (as corresponds to a zwitterion) but also each of the SAFT-γ Mie groups used to model the systems of interest is also a neutral (standard) group. As an example, glycine is modeled as 1 × NH_2_, 1 × CH_2_, and 1 × COOH group, alanine is modeled as 1 × NH_2_, 1 × CH, 1 × CH_3_, and 1 × COOH group, while serine contains 1 × NH_2_, 1 × CH, 1 × CH_2_OH, and 1 × COOH group. A representation of these models is given in Figure 2.

**Figure 3:**
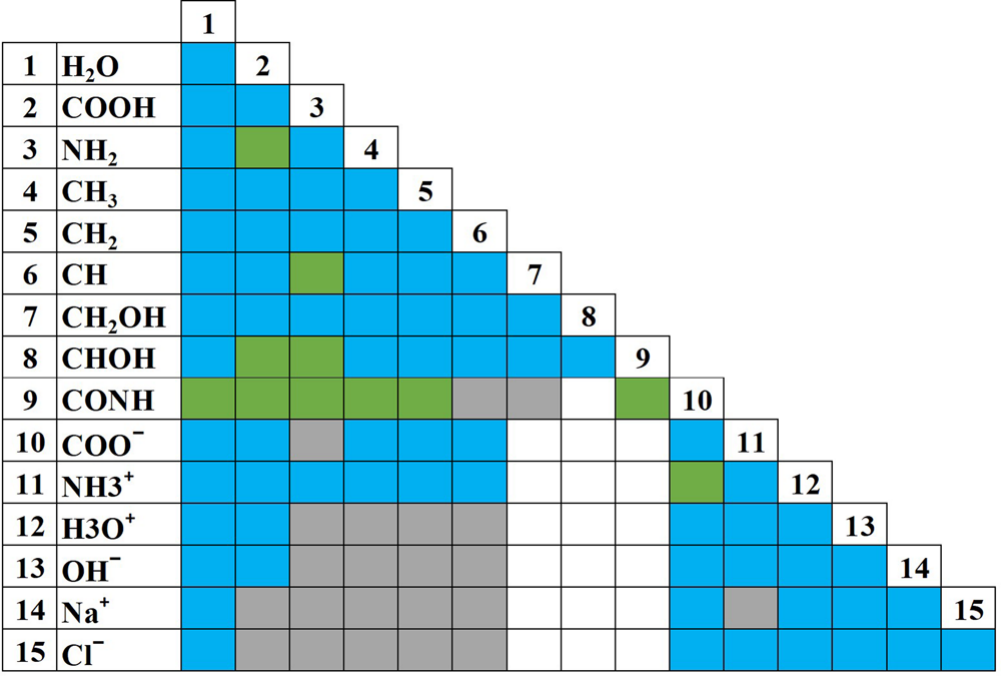
SAFT-γ Mie GC group interaction submatrix containing groups required to model systems in the current work. Blue cells indicate interactions that have been previously estimated;^47,48^ green cells indicate interactions developed in our current work; gray cells indicate interactions that are obtained using combining rules;^41^ and white cells indicate interactions that are not needed in the current work.

We consider glycine, alanine, serine, valine, and leucine, and several small di- and tripeptides of these amino acids, with water and alcohols as solvents. As we assume the system to be at the isoelectric point and use neutral models only, we model the mixtures without the need to treat speciation of the zwitterion or any of the solvents at this point. The similarity in structure of the amino acids and peptides and the group-contribution nature of the SAFT-γ Mie approach mean that a small number of groups is sufficient to model the properties of all the molecules of interest here. Specifically, the uncharged H_2_O, COOH, NH_2_, CH_3_, CH_2_, CH, CH_2_OH, CHOH, and CONH groups are used in this section. The relevant parameter submatrix is shown in Figure 3. Most of these groups and their like and unlike interactions have been characterized in previous work, with one new group and 11 unlike interactions needing to be determined as part of the current work. The interaction parameters of the groups, shown in Tables 1, 2, and 3, are optimized by adjustment using experimental thermodynamic-property data of pure-component and binary systems that contain the groups of interest. The percent absolute average deviation (%AAD) and the absolute average deviation (AAD), which are used to quantify the accuracy of the model description, are defined as follows:
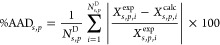
23
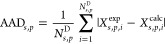
24where *N*_*s*,*p*_^D^ is the number of experimental data points, *X*_*s*,*p*,*i*_^exp^, of property *p* for system *s*, and *X*_*s*,*p*,*i*_^cal^ are the corresponding calculated values. More details on the parameter-optimization strategy, which has been used in past work, can be found in refs (48, 49, and 74). The interactions determined as part of the current work are described below.

**Table 1:** SAFT-γ Mie Interaction Parameters of the Groups Considered in Our Current Work (Excluding Association)^a^

Group *k*	ν_k_*	*S*_*k*_	σ_*kk*_/Å	σ_*kk*_^Born^/Å	λ_*kk*_^r^	(ε_*kk*_/*k*_B_)/K	*N*_ST,k_	*n*_*k*,H_	*n*_*k*,e_1__	*n*_*k*,e_2__	ref
H_2_O	1	1.0000	3.0063		17.020	266.68	2	2	2		(43,44)
COOH	1	0.55593	4.3331		8.0000	405.78	3	1	2	2	(42)
NH_2_	1	0.79675	3.2477		10.254	284.78	2	2	1		(75)
CH_3_	1	0.57255	4.0772		15.050	256.77					(41)
CH_2_	1	0.22932	4.8801		19.871	473.39					(41)
CH	1	0.072100	5.2950		8.0000	95.621					(42)
CH_2_OH	2	0.58538	3.4054		22.699	407.22	2	1	2		(76)
CHOH	2	0.18963	4.5381		18.185	599.66	2	1	2		(48)
CONH	2	0.73764	2.9482		29.839	156.12	2	1	2		*
COO^–^	1	0.55593	4.3331	4.6364	8.0000	21.264	1		4		(52)
NH_3_^+^	1	0.79675	3.2477	3.4750	10.254	48.300	1	3			(47)
H_3_O^+^	1	1.0000	3.0063	3.0063	17.020	68.190	1	3			(65)
OH^–^	1	1.0000	2.4600	3.0063	17.020	170.24	1		3		(65)
Na^+^	1	1.0000	2.3200	3.3600	12.000	31.711					(65)
Cl^–^	1	1.0000	3.3400	3.8740	12.000	113.77					(65)

a The attractive range of the Mie potential is λ_*kk*_^a^ = 6 for all groups here. The asterisk indicates that the CONH group are characterized in the current work.

**Table 2:** Unlike Group Parameters (Excluding Association) for Use with the SAFT-γ Mie Approach^a^

group *k*	group *l*	(ε_*kl*_/*k*_B_)	λ_*kl*_^r^	ref	group *k*	group *l*	(ε_*kl*_/*k*_B_)	λ_*kl*_^r^	ref
H_2_O	COOH	289.76	CR	(76)	CH_3_	COO^–^	255.99	CR	(51,52)
H_2_O	NH_2_	358.55	CR	(75)	CH_3_	NH_3_^+^	244.15	CR	(47)
H_2_O	CH_3_	358.18	100.00	(76)	CH_3_	H_3_O^+^	CR	CR	
H_2_O	CH_2_	423.63	100.00	(76)	CH_3_	Na^+^	CR	CR	
H_2_O	CH	275.75	CR	(77)	CH_3_	Cl^–^	CR	CR	
H_2_O	CH_2_OH	353.37	CR	(76)	CH_3_	OH^–^	CR	CR	
H_2_O	CHOH	479.16	CR	(48)	CH_3_	CONH	430.60	CR	*
H_2_O	COO^–^	171.61	CR	(51,52)	CH_2_	CH	506.21	CR	(42)
H_2_O	NH_3_^+^	450.21	CR	(47)	CH_2_	CH_2_OH	423.17	CR	(76)
H_2_O	H_3_O^+^	391.04	CR	(65)	CH_2_^†^	CHOH	517.64	CR	(48)
H_2_O	OH^–^	134.41	CR	(65)	CH_2_	COO^–^	413.74	CR	(51,52)
H_2_O	CONH	379.59	CR	*	CH_2_	NH_3_^+^	348.39	CR	(47)
H_2_O	Na^+^	539.68	CR	(65)	CH_2_	H_3_O^+^	CR	CR	
H_2_O	Cl^–^	95.406	CR	(65)	CH_2_	OH^–^	CR	CR	
COOH	NH_2_	285.00	CR	*	CH_2_	CONH	315.00	CR	*
COOH	CH_3_	255.99	CR	(42)	CH_2_	Na^+^	CR	CR	
COOH	CH_2_	413.74	CR	(42)	CH_2_	Cl^–^	CR	CR	
COOH	CH	504.99	CR	(42)	CH	CH_2_OH	329.22	CR	(77)
COOH	CH_2_OH	488.18	CR	(78)	CH	CHOH	0	CR	(48)
COOH	CHOH	1154.3	50.000	*	CH	COO^–^	504.99	CR	(47)
COOH	COO^–^	405.78	8.0000	(51,52)	CH	NH_3_^+^	151.01	CR	(47)
COOH	NH_3_^+^	388.58	CR	(47)	CH	H_3_O^+^	CR	CR	
COOH	H_3_O^+^	CR	CR	(47)	CH	OH^–^	CR	CR	
COOH	OH^–^	CR	CR	(47)	CH	CONH	CR	CR	
COOH	CONH	670.04	CR	*	CH	Na^+^	CR	CR	
COOH	Na^+^	CR	CR		CH	Cl^–^	CR	CR	
COOH	Cl^–^	CR	CR		CH_2_OH	CHOH	389.23	CR	(48)
NH_2_	CH_3_	244.15	CR	(75)	CH_2_OH	CONH	CR	CR	
NH_2_	CH_2_	348.39	CR	(75)	COO^–^	NH_3_^+^	26.330	CR	(47)
NH_2_	CH	278.26	CR	*	COO^–^	H_3_O^+^	27.740	CR	(47)
NH_2_	CH_2_OH	528.21	52.305	(79)	COO^–^	OH^–^	44.520	CR	(47)
NH_2_	CHOH	415.54	10.643	*	COO^–^	Na^+^	9.9125	CR	(51,52)
NH_2_	COO^–^	CR	CR		COO^–^	Cl^–^	21.265	CR	(51,52)
NH_2_	NH_3_^+^	284.78	CR	(47)	NH_3_^+^	H_3_O^+^	56.958	CR	(47)
NH_2_	H_3_O^+^	CR	CR		NH_3_^+^	OH^–^	62.238	CR	(47)
NH_2_	OH^–^	CR	CR		NH_3_^+^	Na^+^	CR	CR	
NH_2_	CONH	150.77	CR	*	NH_3_^+^	Cl^–^	65.257	CR	(47)
NH_2_	Na^+^	CR	CR		H_3_O^+^	OH^–^	66.439	CR	(65)
NH_2_	Cl^–^	CR	CR		H_3_O^+^	Na^+^	37.480	CR	(47)
CH_3_	CH_2_	350.77	CR	(41)	H_3_O^+^	Cl^–^	70.552	CR	(65)
CH_3_	CH	387.48	CR	(42)	OH^–^	Na^+^	27.898	CR	(65)
CH_3_	CH_2_OH	333.20	CR	(76)	OH^–^	Cl^–^	123.21	CR	(47)
CH_3_	CHOH	479.38	CR	(48)	Na^+^	Cl^–^	27.938	CR	(65)

a An asterisk indicates that the parameters are characterized in the current work. ^†^ Indicates that the CH_2_−COO^−^ interaction corresponds to that of the Adjacent-CH_2_ group interaciton with COO^−^ as described in refs (51, 52). CR indicates that combining rules are used.^41^

**Table 3:** Group Association Parameters for Use with the SAFT-γ Mie Approach^a^

group *k*	site *a*	group *l*	site *b*	(ε^HB^_kl,ab_/*k*_B_)/K	*K*^HB^_kl,ab_/Å^3^	ref
H_2_O	H	H_2_O	e_1_	1985.4	101.69	(43,44)
H_2_O	e_1_	COOH	H	2567.7	270.09	(76)
H_2_O	H	COOH	e_1_	1451.8	280.89	(76)
H_2_O	H	COOH	e_2_	1252.6	150.98	(76)
H_2_O	H	NH_2_	e_1_	1460.0	179.60	(75)
H_2_O	e_1_	NH_2_	H	1988.3	55.824	(75)
H_2_O	H	CH_2_OH	e_1_	2153.2	147.40	(76)
H_2_O	e_1_	CH_2_OH	H	621.68	425.00	(76)
H_2_O	H	CHOH	e_1_	2140.9	19.478	(48)
H_2_O	e_1_	CHOH	H	2289.1	63.813	(48)
H_2_O	H	COO^–^	e_1_	802.21	52.555	(51,52)
H_2_O	e_1_	NH_3_^+^	H	2016.6	49.397	(47)
H_2_O	e_1_	H_3_O^+^	H	1985.4	101.69	(65)
H_2_O	H	OH^–^	e_1_	1492.0	76.411	(65)
H_2_O	H	CONH	e_1_	1986.2	236.59	*
H_2_O	e_1_	CONH	H	3061.5	130.81	*
COOH	H	COOH	H	6427.9	0.80620	(42)
COOH	H	NH_2_	e_1_	4000.0	100.00	*
COOH	e_1_	NH_2_	H	1446.6	100.00	*
COOH	e_2_	NH_2_	H	1220.1	100.00	*
COOH	H	CH_2_OH	e_1_	3238.4	36.050	(78)
COOH	e_1_	CH_2_OH	H	1062.1	210.67	(78)
COOH	e_2_	CH_2_OH	H	997.89	227.07	(78)
COOH	H	CHOH	e_1_	4000.0	10.834	*
COOH	e_1_	CHOH	H	305.02	1.0000	*
COOH	e_2_	CHOH	H	319.87	0.10000	*
COOH	e_1_	NH_3_^+^	H	334.08	13.500	(47)
COOH	e_2_	NH_3_^+^	H	366.54	9990.0	(47)
COOH	e_1_	H_3_O^+^	H	1451.8	280.89	(47)
COOH	e_2_	H_3_O^+^	H	1252.6	150.98	(47)
COOH	H	OH^–^	e_1_	2036.0	214.16	(47)
COOH	H	CONH	e_1_	2487.2	242.73	*
COOH	e_1_	CONH	H	1723.5	461.80	*
COOH	e_2_	CONH	H	1723.5	461.80	*
NH_2_	e_1_	NH_2_	H	1070.8	95.225	(75)
NH_2_	H	CH_2_OH	e_1_	629.88	346.08	(79)
NH_2_	e_1_	CH_2_OH	H	2403.8	26.192	(79)
NH_2_	H	CHOH	e_1_	1524.9	103.22	*
NH_2_	e_1_	CHOH	H	1470.3	303.47	*
NH_2_	H	COO^–^	e_1_	1220.1	100.00	*
NH_2_	e_1_	NH_3_^+^	H	1070.8	95.225	*
NH_2_	e_1_	H_3_O^+^	H	1460.0	179.60	*
NH_2_	H	OH^–^	e_1_	1511.4	42.436	*
NH_2_	e_1_	CONH	H	2807.1	122.83	*
NH_2_	H	CONH	e_1_	1687.6	122.83	*
CH_2_OH	H	CH_2_OH	e_1_	2097.9	62.309	(76)
CH_2_OH	H	CHOH	e_1_	2500.0	10.444	(48)
CH_2_OH	e_1_	CHOH	H	1464.1	591.55	(48)
CH_2_OH	H	CONH	e_1_	CR	CR	
CH_2_OH	e_1_	CONH	H	CR	CR	
CHOH	H	CHOH	e_1_	2480.6	8.4740	(48)
COO^–^	e_1_	NH_3_^+^	H	1767.0	13.500	*, ^47^
COO^–^	e_1_	H_3_O^+^	H	802.21	52.555	(47)
NH_3_^+^	H	OH^–^	e_1_	1988.3	55.824	(47)
H_3_O^+^	H	OH^–^	e_1_	1492.0	76.411	(65)
CONH	H	CONH	e_1_	3181.7	155.31	*

a An asterisk indicates that the parameters are characterized in the current work.

### NH_2_, COOH, CHOH, and CH Groups

The NH_2_,^75^ COOH,^42^ CHOH,^48^ and CH^42^ groups have been developed in previous work, but the NH_2_–COOH, NH_2_–CHOH, NH_2_–CH, and COOH–CHOH unlike interactions need to be characterized. We use experimental data of simple mixtures containing these groups and aim to include only limited amino-acid data in the parameter estimation, as we are interested in developing a SAFT-γ Mie framework that is predictive for the properties of amino acid and peptide solutions.

**Figure 4:**
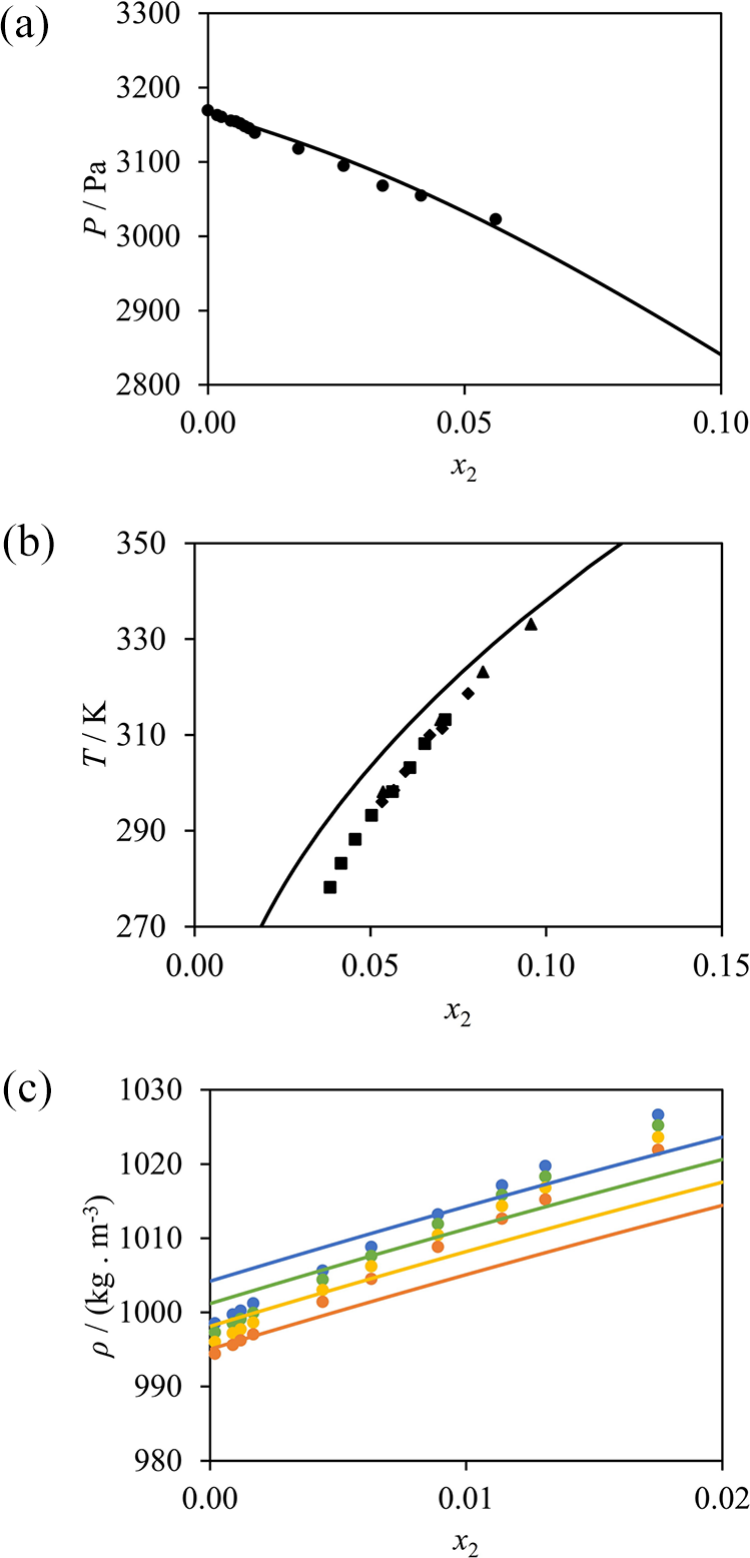
SAFT-γ Mie calculations (curves) used in the estimation of the NH_2_–COOH unlike interaction compared to experimental data (symbols). (a) Bubble pressure of water (1) + glycine (2) at 298 K.^82^ (b) Solid–liquid solubility of water (1) + glycine (2) at 1 bar; circles,^80^ squares,^84^ diamonds,^81^ and triangles.^22^ (c) Liquid densities of water (1) + 3-amino-propanoic acid (2)^83^ at 293.15 K (blue); 298.15 K (green); 303.15 K (yellow); and 308.15 K (orange).

The NH_2_–COOH interaction is key to model amino acids and peptides, however, its optimization represents a major challenge due to the lack of experimental data for mixtures of primary amines (R-NH_2_) and alkanoic acids (R-COOH) as a result of the reactive nature of these mixtures. Instead, we use experimental aqueous-mixture data of amino acids belonging to the glycine homologous series H_2_N-(CH_2_)_*n*_–COOH with *n* = 1–5. These amino acids are chosen to minimize the influence of groups, other than NH_2_ and COOH, on the resulting interaction parameters.^74^ We take into account the SLE^22,80,81^ and VLE^82^ of glycine in water, and the liquid density of aqueous solutions^83^ of glycine, 3-aminopropanoic acid, 4-aminobutanoic acid, 5-aminobutanoic acid, and 6-aminohexanoic acid. Calculations using the optimized parameters, illustrated in Figure 4, exhibit very good agreement with the available experimental data. Optimization strategies incorporating other combinations of thermodynamic-property data, but excluding solubility, led to nonphysical parameter values and larger deviations between the calculated and experimental solubilities.

The NH_2_–CHOH interaction is optimized by adjustment using experimental data of secondary amine + primary amine mixtures. Specifically: VLE data of 2-butanol + 1-butanamine;^85,86^ excess-enthalpy data of 2-propanol + 2-propanamine,^87^ 2-propanol + 1-butanamine,^88^ 2-propanol + 2-butanamine,^88^ and 2-butanol + 1-butanamine;^85^ and density data of 2-propanol + 1-propanamine,^89^ 2-butanol + 1-propanamine,^89^ 2-butanol + 1-butanamine,^90^ 2-hexanol + 1-butanamine,^91^ and 3-hexanol + 1-butanamine^91^ are considered. The COOH–CHOH interaction is optimized using VLE data of 2-propanol + propanoic acid^92^ and 2-propanol + butanoic acid,^93^ and VLE^92^ and liquid-density^94^ data of 2-butanol + propanoic acid.

**Figure 5:**
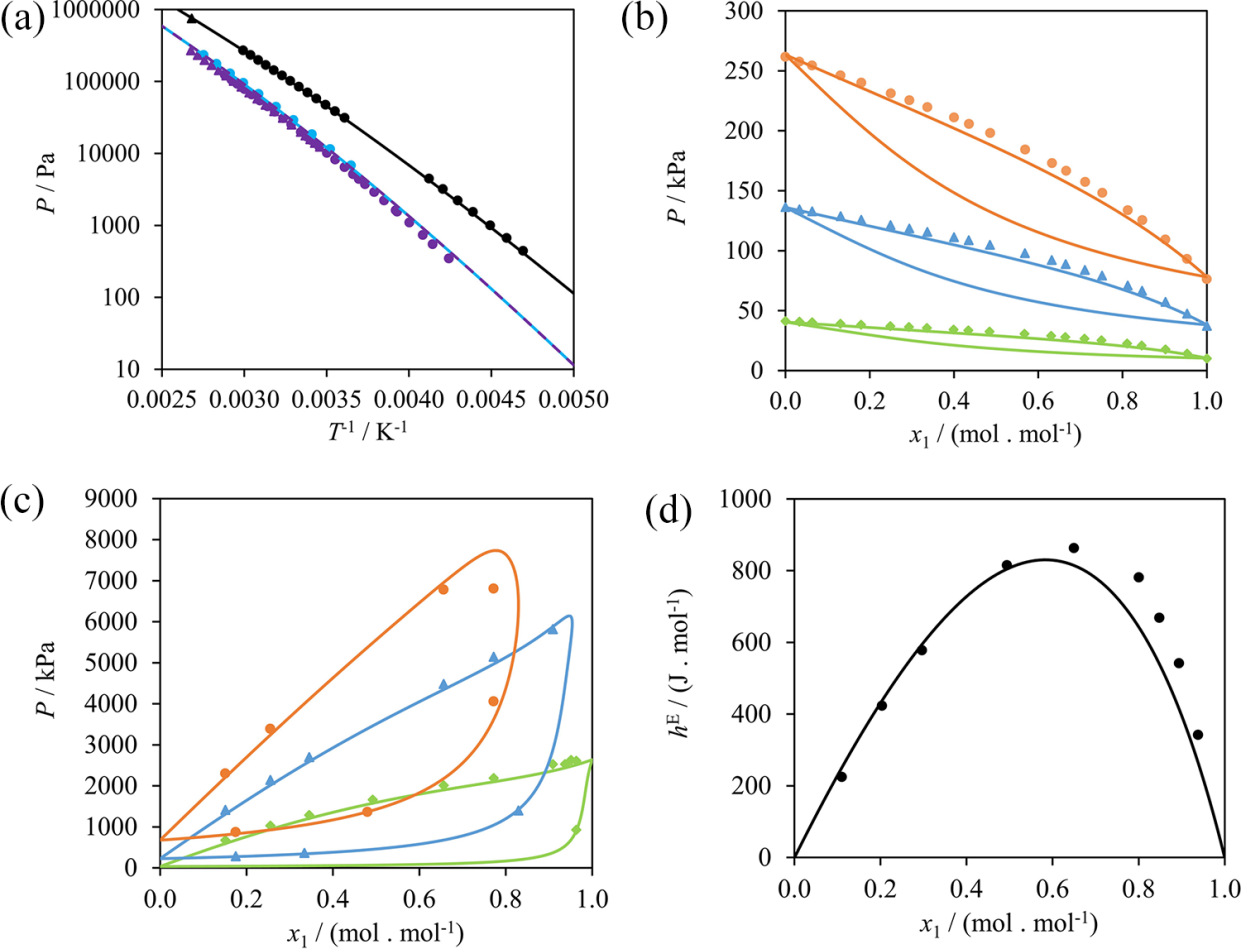
SAFT-γ Mie calculations (curves) of properties used in the estimation of the NH_2_–CH unlike interaction compared to experimental data (symbols). (a) Vapor-pressure of 2-propanamine (black), 2-butanamine (light blue), and 2-methyl-1-propanamine (purple). Symbols denote different experimental sources: black circles;^95^ black triangle;^106^ light blue circles;^96^ purple circles;^98^ and purple triangles.^97^ The SAFT-γ description for 2-butanamine and 2-methyl-1-propanamine is identical because the molecules are comprised of the same groups. (b) Pressure–composition isotherms of hexane (1) + 2-propanamine (2)^103^ at 283.15 K (green), 313.15 K (blue), and 333.15 K (orange). (c) Pressure–composition isotherms of ethane (1) + 2-propanamine (2)^102^ at 279.1 K (green), 328.3 K (blue), and 367.9 K (orange). (d) Excess enthalpy of hexane (1) + 2-butanamine (2) at 298.15 K and 1 bar.^104^

The NH_2_–CH interaction is estimated using experimental vapor-pressure data of 2-propanamine,^95^ 2-butanamine,^96^ and 2-methyl-1-propanamine,^97,98^ liquid-density data of 2-propanamine^99^ and 2-butanamine,^100,101^ VLE data of ethane + 2-propanamine,^102^ and hexane + 2-propanamine^103^ mixtures, and excess-enthalpy data of hexane + 2-butanamine^104^ and heptane + 2-butanamine^105^ mixtures. The theory yields very good agreement with the experimental data, as can be seen in Figure 5. Corresponding %AAD and AAD are presented in Table 4 for each of the systems discussed throughout this work.

**Table 4:** Overview of the Accuracy of SAFT-γ Mie^a^

	*T* range	*P* range		*N*^D^	%AAD	AAD		
system	/K	/kPa	*x*_2_ range		*P*^sat^	*P*^sat^/kPa	figures	ref
2-propanamine	213–373	−	−	21	2.310	1.049	5(a)	(95,106)
2-butanamine	274–363	−	−	10	3.454	1.307	5(a)	(96)
2-methyl-1-propanamine	236–374	−	−	47	17.02	8.158	5(a)	(97,98)
*n*-ethylacetamide	279–423	−	−	59	21.18	0.0964	6(a)	(109,110)
*n*-propylacetamide	297–453	−	−	39	31.42	0.4109	6(a)	(110,111)
*n*-butylacetamide	355–502	−	−	19	6.674	0.9631	6(a)	(112,113)
*n*-pentylacetamide	374–384	−	−	14	34.23	1.283	6(a)	(114)
*n*-methylpropanamide	303–685	−	−	30	22.52	0.2473	6(a)	(109,115)
*n*-methylhexanamide	372–472	−	−	18	19.47	1.221	6(a)	(116)
*n*-butylpropanamide	351–454	−	−	20	4.208	0.0448	6(a)	(111)

	*T* range	*P* range			%AAD	AAD		
system	/K	/kPa	*x*_2_ range	*N*^D^	ρ	ρ/(kg m^–3^)	figures	ref
2-propanamine	213–298	101.33	−	11	2.099	15.14	5(b)	(99)
2-butanamine	288–323	101.33	−	8	1.403	9.976	5(b)	(100,101)
*n*-ethylacetamide	278–318	101.33	−	8	0.3236	3.021	6(b)	(117−119)
*n*-methylpropanamide	288–363	101.33	−	34	0.3699	3.375	6(b)	(120−124)
*n*-methylbutanamide	298–333	101.33	−	5	0.3279	2.950	6(b)	(125)

system	*T* range	*P* range			%AAD	AAD		
(1 + 2)	/K	/kPa	*x*_2_ range	*N*^D^	ρ	ρ/(kg m^–3^)	figures	ref
water + 3-aminopropanoic acid	293–308	101.33	0.0002–0.002	40	0.3274	3.308	4(c)	(83)
water + 4-aminobutanoic acid	293–308	101.33	0.0002–0.002	40	0.3163	3.192	−	(83)
water + 5-aminopentanoic acid	293–308	101.33	0.0002–0.002	40	0.3233	3.261	−	(83)
water + 6-aminohexanoic acid	293–308	101.33	0.0002–0.002	35	0.4528	4.642	−	(83)
2-propanol + 1-propanamine	298–328	81.50	0.00–1.00	52	1.776	13.25	−	(89)
2-butanol + 1-propanamine	298–328	81.50	0.00–1.00	52	0.7513	5.666	−	(89)
2-butanol + 1-butanamine	298–313	101.33	0.05–0.95	21	0.8210	6.248	−	(90)
2-hexanol + 1-butanamine	303–323	101.33	0.00–1.00	33	0.5939	4.539	−	(91)
3-hexanol + 1-butanamine	303–323	101.33	0.00–1.00	33	0.3539	2.696	−	(91)
2-butanol + propanoic acid	293–333	101.33	0.00–1.00	84	1.345	12.05	−	(94)
water + *n*-methylacetamide	298–323	101.33	0.00–1.00	107	1.276	12.47	−	(132,133)
water + *n*-ethylacetamide	298	101.33	0.00–1.00	19	1.934	18.63	−	(132)
water + *n*-methylpropanamide	293–313	101.33	0.00–1.00	20	1.068	10.29	−	(132)
water + methyl urea	274–333	101.33	0.00–0.12	256	0.7603	7.732	9(b)	(138−140,143)
water + ethyl urea	288–323	101.33	0.00–0.13	76	0.7495	7.585	9(b)	(140,141)
water + butyl urea	288–308	101.33	0.00–0.005	30	0.5422	5.414	9(b)	(140)

system	*T* range	*P* range			%AAD	AAD		
(1 + 2)	/K	/kPa	*x*_2_ range	*N*^D^	*T*_bub_	*T*_bub_/K	figures	ref
2-propanol + propanoic acid	358–413	101.33	0.00–1.00	18	0.2829	1.101	−	(92)
water + *n*-methylacetamide	373–478	101.33	0.00–1.00	82	0.4685	1.992	8(a)	(128)
acetic acid + *n*-methylacetamide	391–478	101.33	0.00–1.00	31	1.639	6.879	10(b)	(142)

system	*T* range	*P* range			%AAD	AAD		
(1 + 2)	/K	/kPa	*x*_2_ range	*N*^D^	*T*_dew_	*T*_dew_/K	figures	ref
2-propanol + propanoic acid	358–413	101.33	0.00–1.00	18	0.5097	1.986	−	(92)
water + *n*-methylacetamide	373–478	101.33	0.00–1.00	82	0.7799	3.303	8(a)	(128)
acetic acid + *n*-methylacetamide	373–473	101.33	0.00–1.00	31	0.6934	2.945	10(b)	(142)

system	*T* range	*P* range			%AAD	AAD		
(1 + 2)	/K	/kPa	*x*_2_ range	*N*^D^	*P*_bub_	*P*_bub_/kPa	figures	ref
water + glycine	298	3.0–3.2	0.00–0.06	14	0.6563	0.0205	4(a)	(82)
2-butanol + 1-butanamine	328	14–46	0.00–1.00	12	9.012	2.178	−	(85)
2-propanol + butanoic acid	333–373	1–194	0.00–1.00	50	15.61	5.200	−	(93)
ethane + 2-propanamine	279–372	676–6850	0.04–0.85	100	4.342	140.8	5(c)	(102)
hexane + 2-propanamine	283–333	10–262	0.00–1.00	63	5.263	4.172	5(d)	(103)
*n*-octane + *n*-methylacetamide	363–398	2–98	0.00–1.00	45	26.82	9.460	7(a)	(109,127)
*n*-decane + *n*-methylacetamide	414	12–50	0.00–1.00	43	30.09	8.660	−	(127)
*n*-decane + *n*-ethylacetamide	363–383	1–17	0.00–1.00	24	8.173	0.7078	−	(109)
*n*-octane + *n*-methylpropanamide	363–383	1–64	0.00–1.00	30	12.12	4.098	−	(109)
water + *n*-methylacetamide	313–413	0.05–313	0.00–1.00	67	5.957	2.596	8(b)	(126,136,137)

system	*T* range	*P* range			%AAD	AAD		
(1 + 2)	/K	/kPa	*x*_2_ range	*N*^D^	*P*_dew_	*P*_dew_/kPa	figures	ref
2-butanol + 1-butanamine	328	14–46	0.00–1.00	12	9.192	2.503	−	(85)
2-propanol + butanoic acid	333–373	1–194	0.00–1.00	50	15.75	6.093	−	(93)
ethane + 2-propanamine	279–369	208–4860	0.04–0.82	73	5.958	153.0	5(c)	(102)
water + *n*-methylacetamide	313–373	0.05–69	0.00–1.00	47	15.58	1.780	8(b)	(136,137)

system	*T* range	*P* range			%AAD	AAD		
(1 + 2)	/K	/kPa	*x*_2_ range	*N*^D^	*T*_LLE_	*T*_LLE_/K	figures	ref
*n*-octane + *n*-methylpropanamide	310–361	101.33	0.04–0.70	8	5.638	19.75	7(b)	(109)

system	*T* range	*P* range			%AAD	AAD		
(1 + 2)	/K	/kPa	*x*_2_ range	*N*^D^	*H*^E^	*H*^E^/(J mol^–1^)	figures	ref
2-propanol + 2-propanamine	298	101.33	0.10–0.90	9	5.422	85.24	−	(87)
2-propanol + 1-butanamine	298	101.33	0.00–0.90	137	18.70	289.6	−	(88)
2-propanol + 2-butanamine	298	101.33	0.01–0.99	11	23.69	218.0	−	(88)
2-butanol + 1-butanamine	298	101.33	0.10–0.95	11	14.32	172.0	−	(85)
*n*-hexane + 2-butanamine	298	101.33	0.06–0.89	9	12.04	65.52	5(e)	(104)
*n*-heptane + 2-butanamine	298	101.33	0.05–0.95	19	21.51	161.2	5(f)	(105)
*n*-octane + *n*-methylacetamide	398	1891	0.00–1.00	5	50.56	101.8	7(c)	(126)
*n*-decane + *n*-methylacetamide	413	1617	0.00–1.00	2	90.98	101.1	7(c)	(126)
water + *n*-methylacetamide	323–398	101.33	0.01–0.95	43	15.29	103.7	8(c)	(134)
water + *n*-ethylacetamide	308	101.33	0.02–0.98	18	14.14	110.6	8(c)	(135)
water + *n*-methylpropanamide	308	101.33	0.03–0.98	17	16.20	109.8	8(c)	(135)
1-hexanamine + *n*-methylacetamide	363	1203	0.03–0.96	20	15.33	37.55	9(a)	(134)
propanpoic acid + *n*-methylacetamide	363	1135	0.03–0.98	21	31.35	125.1	10(a)	(134)

system	*T* range	*P* range			AAD		
(1 + 2)	/K	/kPa	*x*_2_ range	*N*^D^	*x*_2_^SLE^	figures	ref
2-propanol + octanoic acid	272–283	101.33	0.50–0.80	2	0.04318	−	(144)
2-propanol + nonanoic acid	273–283	101.33	0.60–0.90	2	0.03696	−	(144)
2-propanol + decanoic acid	273–303	101.33	0.20–0.95	4	0.02287	−	(144)
2-propanol + undecanoic acid	273–293	101.33	0.20–0.60	3	0.02873	−	(144)
2-propanol + dodecanoic acid	273–318	101.33	0.05–1.00	31	0.01339	−	(144−146)
2-propanol + tridecanoic acid	273–313	101.33	0.05–0.95	5	0.01983	−	(144)
2-propanol + tetradecanoic acid	273–323	101.33	0.00–0.80	6	0.01742	−	(144)
2-propanol + pentadecanoic acid	273–323	101.33	0.02–0.80	6	0.01436	−	(144)
2-propanol + hexadecanoic acid	273–336	101.33	0.00–1.00	28	0.04348	−	(144,146,147)
2-propanol + heptadecanoic acid	273–333	101.33	0.00–0.94	7	0.01805		(144)
2-propanol + octadecanoic acid	283–344	101.33	0.00–1.00	42	0.04208	−	(144,146,148,149)

a Represented in %AAD and AAD, in the calculation of vapor pressure (*P*^sat^), liquid pure-component and mixture densities (ρ), bubble temperature (*T*_bub_), dew temperature (*T*_dew_), bubble pressure (*P*_bub_), dew pressure (*P*_dew_), LLE temperature (*T*_LLE_), and excess enthalpy (*H*^E^). *N*^D^ is the number of experimental data points used to evaluate the accuracy of the model.

### CONH Amide Group

We have considered the possibility of modeling the amide functional group as separate C=O and NH groups, which have been parametrized in previous work,^75,107^ but we find that such a description does not lead to an accurate representation of the properties of aqueous dipeptides. This is most likely because the C=O and NH groups were parametrized using experimental data of 2-ketones and secondary amines, respectively. In these families, the two groups are not adjacent, and large polarization effects that arise when the two groups are adjacent, as is the case in peptide molecules, are therefore neglected. Hence, to model mixtures containing peptides, a new group CONH is introduced and parametrized using experimental data of amides (R-CONH-R′), which are structurally similar to peptides. The CONH group is modeled with three associating sites, two of type e (e_1_ in Tables 1–3), representing the lone pairs of the oxygen atom, and one of type H corresponding to the hydrogen. In contrast to our treatment of amine groups, we find that it is not necessary to add a further e-type site to account for the electron pair of the nitrogen atom. This may reflect that the ground state of an amide is stabilized by the delocalization of the nitrogen lone-pair electrons through orbital overlap with the carbonyl group of the amide;^108^ this delocalization is the principal reason that amides are nonbasic in nature, whereas amines, in which the nitrogen lone pair is not delocalized, are quite strong bases.

**Figure 6:**
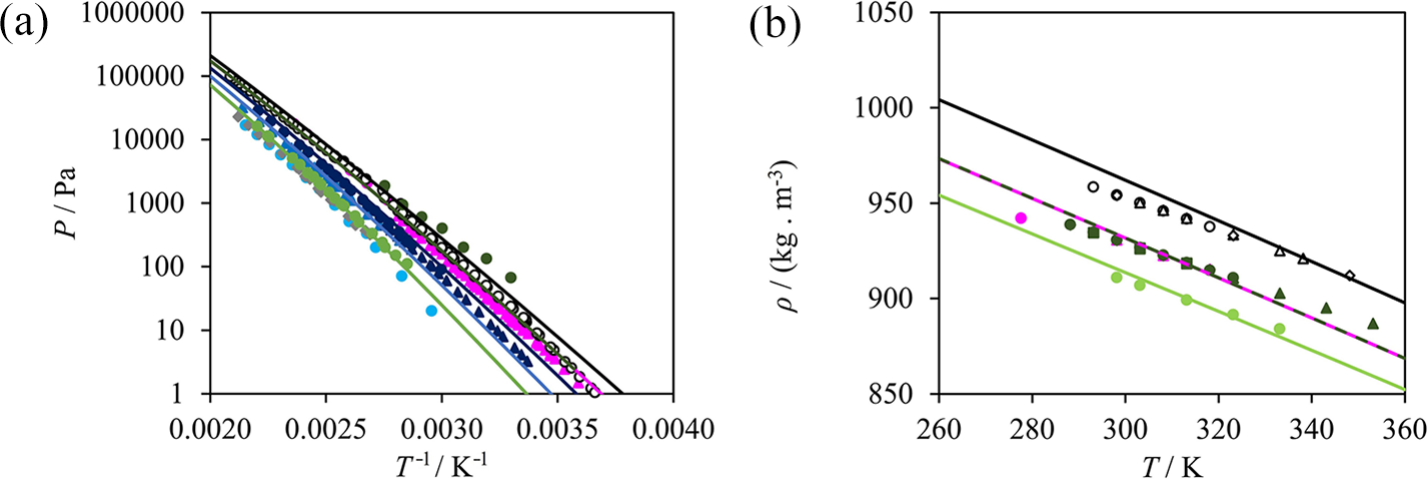
SAFT-γ Mie description of pure-component properties used in the estimation of the CONH–CONH, CONH–CH_3_, and CONH–CH_2_ interactions. Empty symbols denote data not used in the parameter estimation, filled symbols denote those used in the optimization, and curves denote SAFT-γ Mie calculations. (a) Vapor pressures of *n*-methylacetamide (black),^128,129^*n*-ethylacetamide (pink),^109,110^*n*-propylacetamide (navy blue),^110,111^*n*-butylacetamide (blue),^112,113^*n*-pentylacetamide (light blue),^114^*n*-methylpropanamide (dark green),^109,115^*n*-butylpropanamide (green),^111^ and *n*-methylhexanamide (gray).^116^*N*-ethylacetamide and *n*-methylpropanamide comprise the same groups and are therefore represented by the same calculation (pink curve); similarly for *n*-propylacetamide and *n*-methylbutanamide (dark blue curve), and *n*-pentylacetamide, *n*-butylpropanamide, and *n*-methylhexanamide (light blue curve). (b) Isobaric liquid density of *n*-methylacetamide (black),^130,131^*n*-ethylacetamide (pink),^117−119^*n*-methylpropanamide (dark green),^120−124^ and *n*-methylbutanamide (light green)^125^ at 1 bar. *N*-ethylacetamide and *n*-methylpropanamide comprise the same groups and are therefore represented by the same calculation (pink curve).

**Figure 7:**
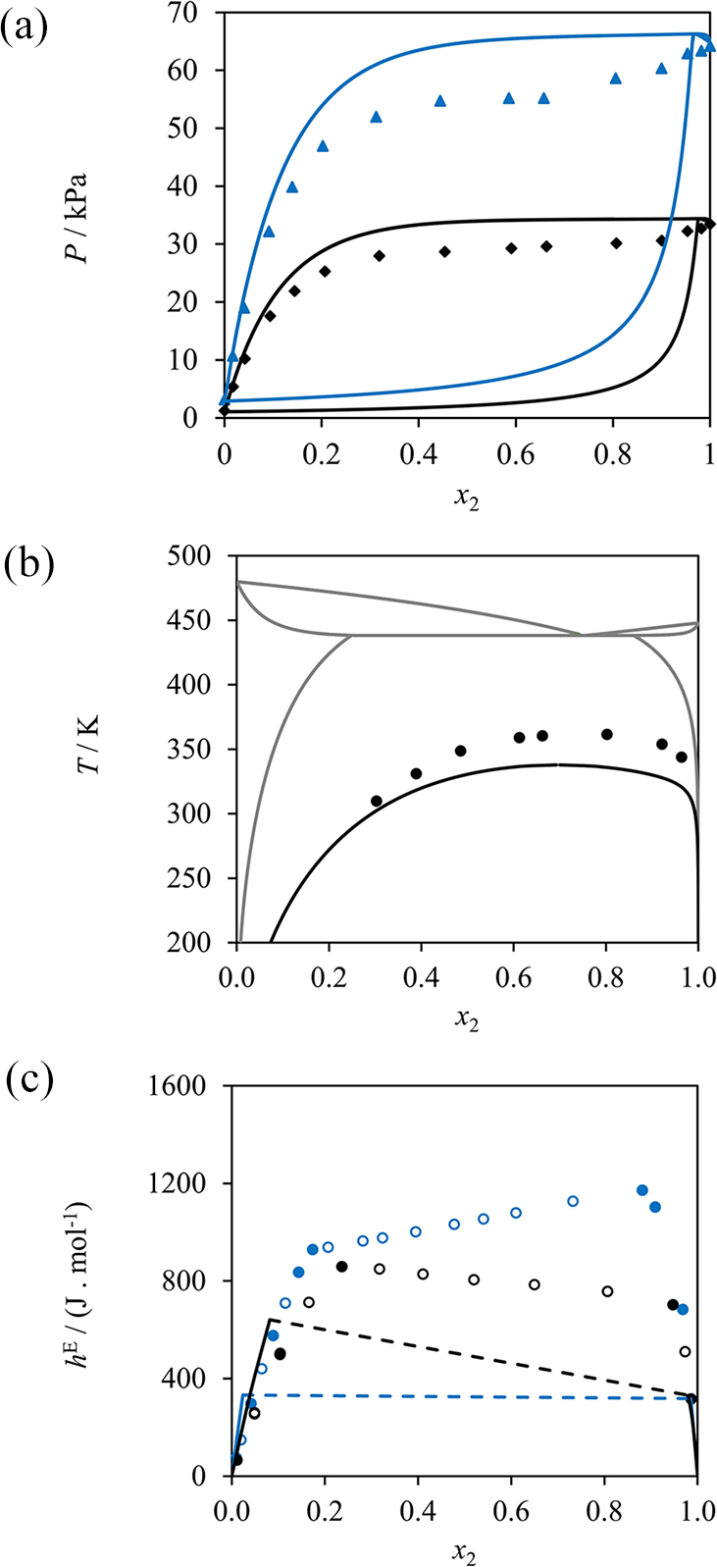
SAFT-γ Mie description of mixture properties used in the estimation of the CONH–CONH, CONH–CH_3_, and CONH–CH_2_ interactions. Filled symbols denote data that were used in the parameter estimation and empty symbols denote data used for validation only; curves represent SAFT calculations. (a) Pressure–composition isotherms illustrating the vapor–liquid equilibrium of *n*-methylpropanamide (1) + *n*-octane (2)^109^ at 363.15 K (black) and 383.15 K (blue). (b) Temperature–composition isobar illustrating the liquid–liquid equilibrium of *n*-methylpropanamide (1) + *n*-octane (2) at 1 bar (black),^109^ and the vapor–liquid–liquid equilibrium of *n*-methylpropanamide (1) + *n*-decane (2) at 1 bar (gray). (c) Excess enthalpies of *n*-methylacetamide (1) + *n*-decane (2) at 413.15 K and 1.617 MPa (blue),^126^ and *n*-methylacetamide (1) + *n*-octane (2) at 398.15 K and 1.891 MPa (black).^126^

The CONH–CONH, CONH–CH_3_, and CONH–CH_2_ interaction parameters are estimated simultaneously by using pure *n*-alkylamide and *n*-alkylamide + *n*-alkane mixture data. Specifically, pure-compound vapor-pressure data of *n*-ethyl-acetamide,^109,110^*n*-propyl-acetamide,^110,111^*n*-butylacetamide,^112,113^*n*-pentyl-acetamide,^114^*n*-methyl-propanamide,^115^*n*-methyl-hexanamide,^116^ and *n*-butyl-propanamide,^111^ and liquid-density data of *n*-ethyl-acetamide,^117−119^*n*-methyl-propanamide,^120−124^ and *n*-methyl-butanamide^125^ are considered. Mixture bubble-pressure and excess-enthalpy data of *n*-decane + *n*-methyl-acetamide,^126^*n*-octane + *n*-methyl-acetamide,^109,127^ bubble-pressure of *n*-decane + *n*-ethyl-acetamide^109^ and bubble-pressure and liquid–liquid-equilibrium (LLE) data of *n*-octane + *n*-methyl-propanamide^109^ are also used. Selected systems used in this parametrization are represented in Figures 6 and 7. As can be seen from the figures, the SAFT-γ Mie calculations yield a good agreement with experimental data of the pure and mixed systems, especially considering the stringent test of delivering vapor–liquid as well as a small region of liquid–liquid equilibrium observed in the mixture of *n*-methylpropanamide + *n*-octane. The cloud curve for a mixture of *n*-methylpropanamide + *n*-decane is also shown in Figure 7(b), for completeness, although no experimental data are currently available for this mixture; the region of liquid–liquid demixing can be gleaned on inspection of the excess-enthalpy data in Figure 7(c). The extrapolative suitability of the group parameters is validated by comparison to the limited number of vapor-pressure and liquid-density of *n*-methylacetamide^128^ data points not included in the parameter estimation.

**Figure 8:**
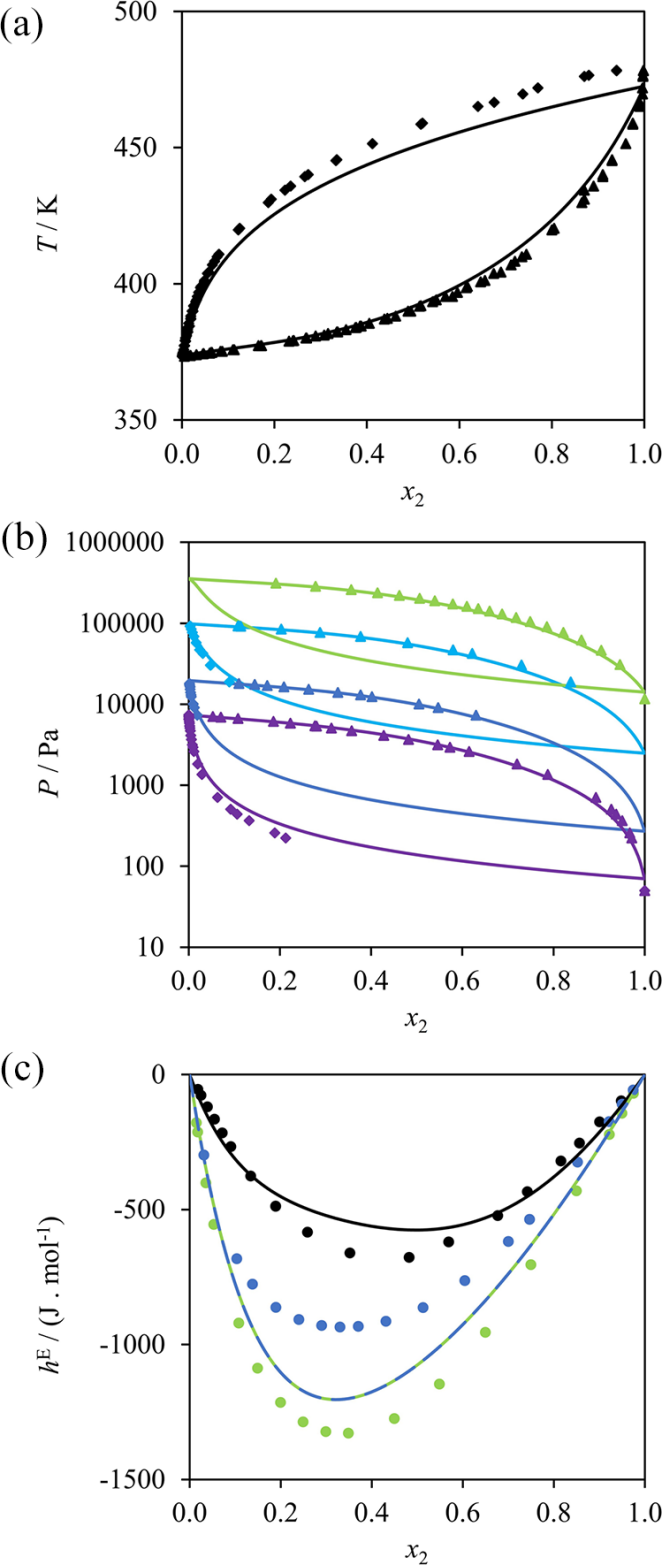
SAFT-γ Mie description of properties used in the estimation of the CONH–H_2_O interaction. (a) Isobaric vapor–liquid equilibrium of water (1) + *n*-methylacetamide (2) at 1 bar.^128^ (b) Isothermal vapor–liquid equilibrium of water (1) + *n*-methylacetamide (2) at 313 K (purple),^136^ 333 K (dark blue),^137^ 373 K (light blue),^137^ and 413 K (light green).^126^ (c) Excess enthalpies of water (1) + *n*-methylacetamide (2) at 398.15 K and 1 bar (black),^134^ water (1) + *n*-methylpropanamide (2) at 308.15 K and 1 bar (blue),^135^ and water (1) + *n*-ethylacetamide (2) at 308.15 K and 1 bar (green).^135^*N*-ethylacetamide and *n*-methylpropanamide are made up of the same functional groups, and their SAFT-γ Mie calculations are identical (green and blue dashed curve).

The interaction CONH−H_2_O, which is crucial in modeling the aqueous solubility of peptides, is estimated using vapor–liquid equilibrium,^126,128^ density^132,133^ and excess-enthalpy^134^ data of water + *n*-methylacetamide mixtures and density,^132^ and excess-enthalpy data^135^ of water + *n*-ethylacetamide and water + *n*-methylpropanamide mixtures. The optimized parameters lead to calculations in good agreement with experiment (cf. Table 4). Selected phase diagrams comparing our SAFT-γ Mie calculations and the experimental data used in parameter development are presented in Figure 8. As can be seen, an accurate description of the bubble and dew pressures is achieved. The excess enthalphies of the water + *n*-methylacetamide and water + *n*-ethylacetamide mixtures are also described in close agreement with the available data, while in the case of the water + *n*-methylpropanamide mixture the calculations present slightly larger deviations from the data. In a SAFT-γ Mie model using first-order groups, such as those developed in the current work, *n*-ethylacetamide and *n*-methylpronanamide are modeled with identical groups and as such have identical calculated properties. Experimentally, however, the two molecules have noticeably different values of the excess enthalpy (Figure 8(c)).

**Figure 9:**
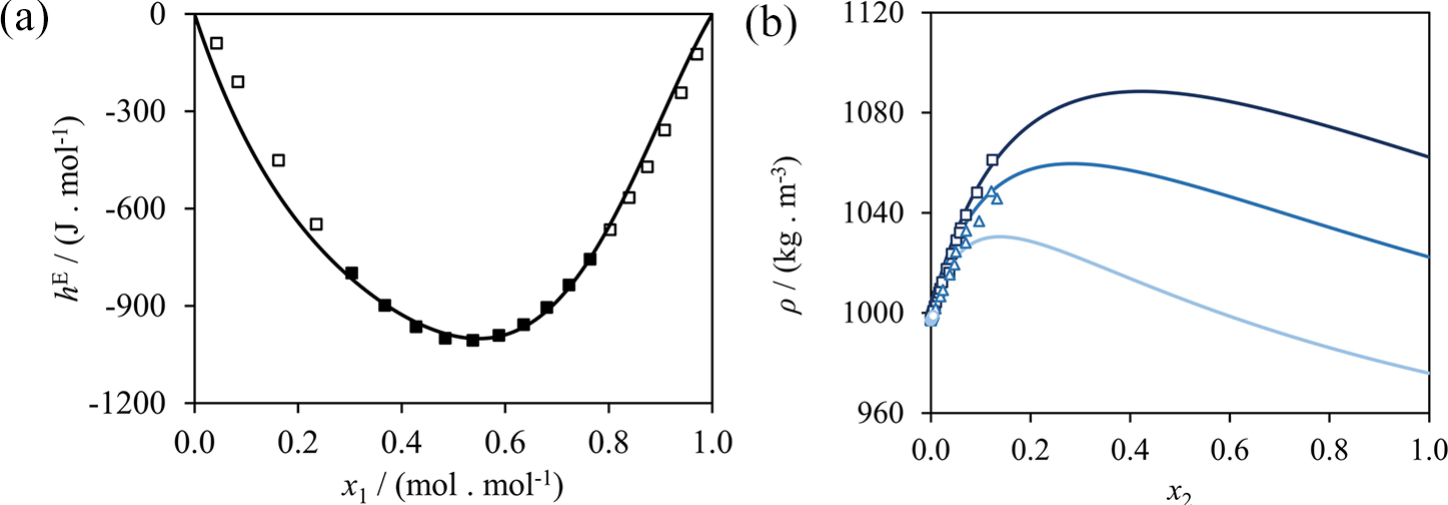
SAFT-γ Mie description of mixture properties used in the parameter estimation and validation of the CONH–NH_2_ interaction. Filled symbols denote data that are used in the parameter optimization and empty symbols denote data used for validation only; curves denote SAFT-γ Mie calculations and predictions. (a) Excess enthalpy of 1-hexanamine (1) + *n*-methylacetamide (2) at 363.15 K and 12.03 bar.^134^ (b) Liquid density for mixtures at 298.15 K and 1 bar of water (1) + methylurea (2) in dark blue and squares,^138−140,143^ water (1) + ethylurea (2) in blue and triangles,^140,141^ and water (1) + butylurea (2) in light blue and circles.^140^

**Figure 10:**
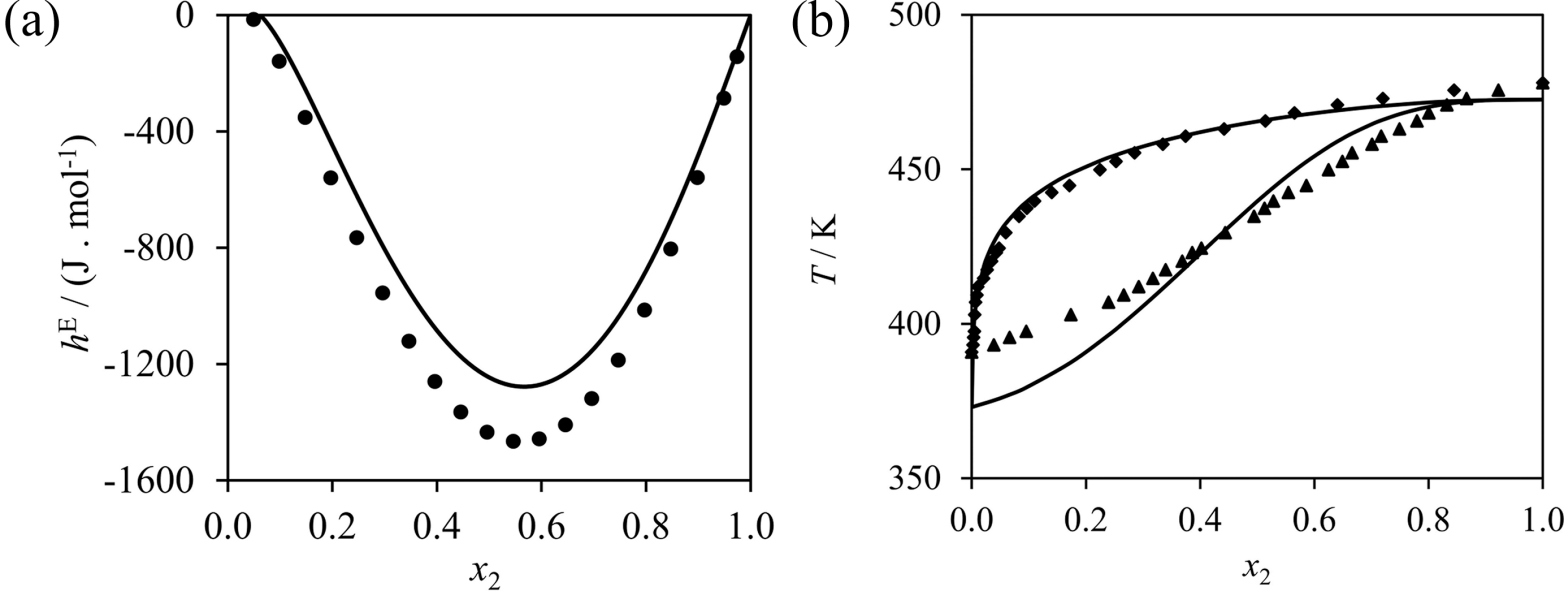
SAFT-γ Mie description of mixture properties used in the parameter estimation of the interaction CONH–COOH. (a) Excess enthalpy of propanoic acid (1) + *n*-methylacetamide (2) at 363.15 K and 11.35 bar.^134^ (b) Isobaric vapor–liquid equilibrium of acetic acid (1) + *n*-methylacetamide (2) at 1 bar;^142^ diamonds and triangles denote dew and bubble temperature data, respectively.

The unlike interactions between CONH, NH_2_, and COOH, also need to be characterized in order to model peptides in our approach. Due to the lack of experimental data of mixtures containing the CONH and NH_2_ groups, the cross-interaction is optimized using only one set of excess-enthalpy data of *n*-methylacetamide + 1-hexanamine mixtures.^134^ The parameters obtained are validated by predicting the liquid density of aqueous mixtures of alkylureas (methyl-,^138−140^ ethyl-,^140,141^ and butylurea,^140^ are considered). As can be seen in Figure 9, the optimized parameters yield predictive calculations of the density of these solutions in very good agreement with the experimental data available. The CONH–COOH interaction is optimized using experimental data of alkanoic acid + *n*-methylacetamide mixtures, although as with the previous groups, few experimental data are found for mixtures including these two groups alone. The optimization is carried out using excess-enthalpy data of propanoic acid + *n*-methylacetamide,^134^ and isobaric VLE and density data of acetic acid + *n*-methylacetamide.^142^ The resulting calculations are compared to the experimental data in Figure 10. As can be seen, the excess enthalpy of the propanoic acid + *n*-methylacetamide mixtures is described in reasonably good agreement with the data (note the small units of J mol^–1^), but in the case of the acetic acid + *n*-methylacetamide VLE, larger deviations are seen. The underestimation of the saturation temperature of pure acetic acid is especially noticeable. A degree of deviation between the SAFT-γ Mie calculations and experiment for acetic-acid mixtures is expected, as no acetic-acid data were used in optimizing the previously characterized COOH–COOH like and COOH–CH_3_ unlike interactions,^42^ added to the fact that in such a small molecule the group-contribution proposition is likely to be inappropriate.^48^ The decision to include acetic acid to characterize first-order groups here is based on the scarcity of other alkanoic acid + amide mixture data.

### Solid–Liquid Equilibrium without Speciation: Thermodynamic Relations and Predictions

The calculation of SLE, at given *T* and *P*, requires the equality of chemical potentials in the solid and liquid phases of any species *i* present in both phases. Assuming that no solvent molecules are present in the solid phase, i.e., that the solid phase is pure amino acid or peptide, and choosing the subcooled liquid of *i* to define the reference state, the well-known solubility equation^150^ follows:
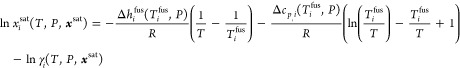
25where ***x***^sat^ is the solid–liquid saturation composition (the solubility) of *i*, *R* the ideal gas constant, *T*_*i*_^fus^ the melting-point temperature of *i*, *Δh*_*i*_^fus^ the corresponding enthalpy of fusion, and *Δc*_*p*,*i*_ = *c*_*p*,*i*_^L^ – *c*_*p*,*i*_^S^ the difference between the molar heat capacity of liquid and solid phases evaluated at *T*_*i*_^fus^; γ_*i*_ is the activity coefficient of *i* (calculated here using SAFT-γ Mie), at the system *T* and *P* and saturation composition. The second term in eq 25 is often neglected, especially when the difference between *T* and *T*_*i*_^fus^ is small.^151^ We neglect this term only when relevant *Δc*_*p*,*i*_ data are not available. The melting properties of amino acids and peptides considered in our current work are obtained from the experimental studies of Do et al.^55,56^ and can be found in Table 5.

**Table 5:** Melting Properties of the Amino Acids and Peptides Used to Calculate Their Solubility^a^

solute	*T*_*i*_^fus^/K	*Δh*_*i*_^fus^/kJ mol^–1^	*Δc*_*p*,*i*_/J mol^–1^ K^–1^	ref
glycine	569 ± 9	22 ± 3		(152)
alanine	608 ± 9	23 ± 3		(152)
valine	529 ± 7	44 ± 6		(55)
leucine	518 ± 8	43 ± 5		(55)
serine	519 ± 7	28 ± 3		(55)
gly-gly	593 ± 7	40 ± 6	51 ± 6	(153)
gly-gly-gly	594 ± 7	54 ± 7	57 ± 15	(56)
ala-ala	606 ± 7	54 ± 7	62 ± 18	(153)
ala-ala-ala	606 ± 7	72 ± 9	124 ± 8	(56)
gly-ala	551 ± 7	41 ± 5	55 ± 6	(153)
ala-gly	611 ± 7	52 ± 7	57 ± 3	(153)
gly-gly-ala	592 ± 10	70 ± 8	66 ± 7	(56)
gly-ala-gly	623 ± 7	61 ± 7	78 ± 11	(56)
ala-gly-ala	557 ± 8	58 ± 7	98 ± 5	(56)
leu-gly-gly	530 ± 7	74 ± 8	111 ± 11	(56)
gly-leu-gly	545 ± 7	60 ± 7	139 ± 11	(56)
gly-gly-leu	521 ± 7	55 ± 7	160 ± 7	(56)
gly-ala-leu	578 ± 7	77 ± 9	112 ± 6	(56)
gly-ser	530 ± 8	49 ± 6	67 ± 6	(56)
ser-gly	553 ± 7	62 ± 7	61 ± 9	(56)
ala-ser	556 ± 7	43 ± 5	48 ± 6	(56)
ser-ala	609 ± 7	73 ± 8	55 ± 3	(56)

a Reported with their experimental uncertainties; melting-point temperature (*T*_*i*_^fus^), enthalpy of fusion (*Δh*_*i*_^fus^), and difference between the molar heat capacity of the liquid and solid phases evaluated at *T*_*i*_^fus^ (*Δc*_*p*,*i*_).

**Figure 11:**
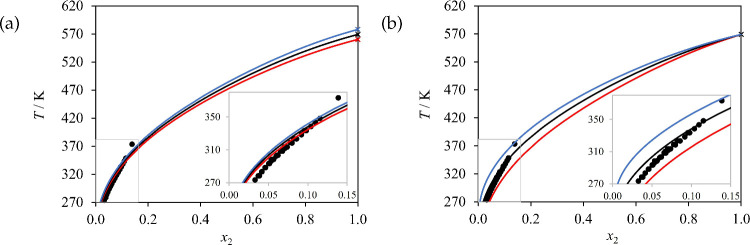
Effect of uncertainty in melting temperature and heat of fusion on the SAFT-γ Mie description of the solid–liquid equilibrium (solubility) of glycine (2) in water (1) at 1 bar. (a) Sensitivity of the solubility calculations to the melting temperature for a fixed enthalpy of fusion of 22 J mol^−1^; the black curve represents calculations using the reported melting temperature (569 K), whereas blue and red curves denote calculations using the maximum (578 K) and minimum (560 K) of the uncertainty range, respectively. (b) The sensitivity of solubility calculations to the enthalpy of fusion for a fixed melting temperature of 569 K; the black curve represents calculations using the reported enthalpy of fusion (22 kJ mol^–1^) whereas blue and red curves denote calculations using the maximum (25 kJ mol^–1^) and minimum (19 kJ mol^–1^) of the uncertainty range, respectively. The symbols (circles) denote the experimental data.^22,80,81,84^

The scarcity of reliable measurements of the melting properties of amino acids and peptides presents a major challenge in modeling their solubility. Amino acids and peptides are known to decompose below their melting points upon slow heating^154^ leading to inconsistent values of the enthalpy of fusion and melting temperature being reported in the literature. Do et al.^55,56^ tried to overcome this challenge by employing fast-scanning calorimetry (FSC) in their work, although their measurements are reported with relatively large uncertainties. For example, the melting point and enthalpy of fusion of glycine were reported as 569 ± 9 K and 22 ± 3 kJ mol^–1^, respectively. In Figure 11 we present solubility calculations using the highest and lowest values of the uncertainty range as well as the reported values. As can be seen, the effect of the reported uncertainty in the melting point on solubility calculations is very small in the region where the solubility data are available (below the boiling point of water); solubility calculations are shown above 373 K in the figure, as the liquid mixture may have a higher saturation temperature that that of pure water, although we note that some of these could correspond to other types of phase equilibria (e.g., liquid–liquid immiscibility)^47^ not explored in the current work. The uncertainty in the enthalpy of fusion, however, leads to clearly different calculated solubilities. We have carried out similar calculations considering the reported uncertainty ranges for each of the amino acids and peptides studied in the current work, and present calculations throughout this work using the reported values of the melting properties obtained experimentally by Do et al.^55,56^ These yield very good predictions of the solubility of the amino acids and di- and tripeptides considered when the neutral models proposed here are used.

**Figure 12:**
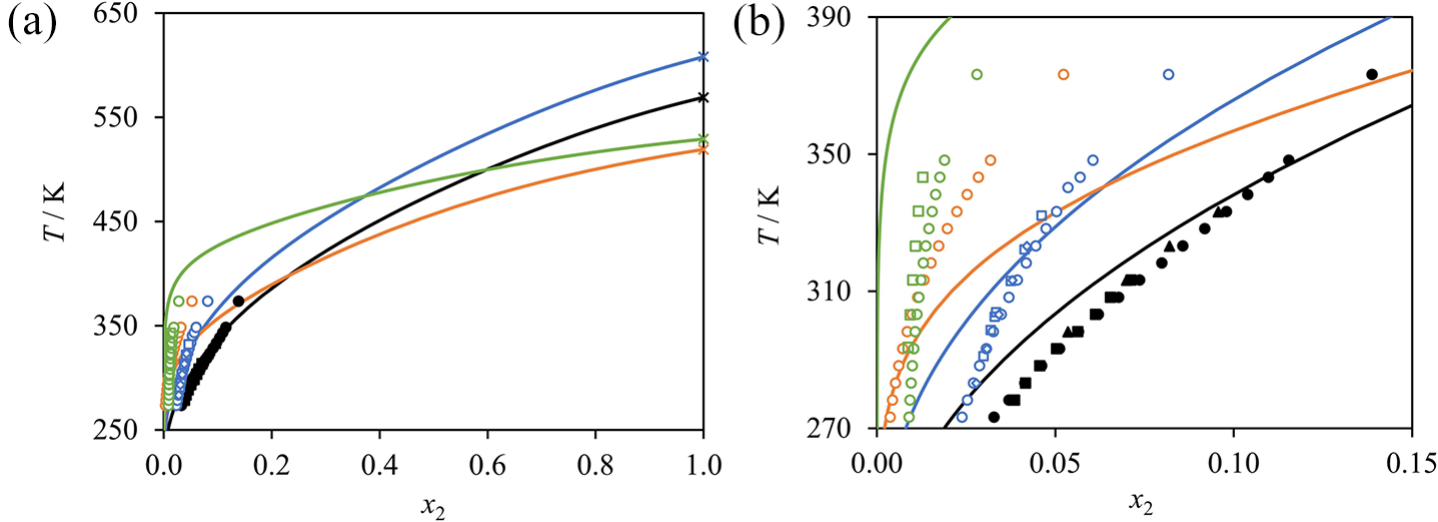
SAFT-γ solid–liquid equilibria (solubility) of amino acid (2) in water (1) at 1 bar; glycine (black); alanine (blue); serine; (orange); and valine (green). (a) Full concentration range depicting the SLE up to the melting points, denoted by “x” symbols. (b) Low amino acid mole-fraction region. The symbols correspond to the experimental data: circles;^80^ squares;^84^ black diamonds;^81^ blue diamonds;^155^ and triangles,^22^ with empty symbols denoting data that are not used in parameter optimization. The curves correspond to the SAFT-γ Mie calculations.

The SLE diagrams of aqueous glycine, alanine, serine, and valine can be seen in Figure 12. Except for glycine (cf. the “NH2, COOH, CHOH, and CH Groups” section), the activity coefficients are entirely predicted, i.e., no solubility data are used in the characterization of the SAFT-γ Mie group parameters. The solubility of alanine is predicted in good agreement with experiment over the entire temperature range measured, while larger deviations can be seen for serine and valine, which have markedly lower solubilities. It is also of interest to note the marked increase in solubility with temperature predicted in the case of serine, which appears to follow a different trend to the other three amino acids. This is likely caused by the presence of the CH_2_OH group and the delicate balance between hydrogen-bonding and dispersion interactions in our model. Valine contains more hydrophobic groups (CH_3_ and CH) than the other amino acids considered here and, as a result, presents the lowest solubility. The temperature dependence of the predicted solubility of valine in water is in overall good agreement with the experimental data, but the predicted values are visibly lower than those measured (e.g., *x*_valine_ = 0.0001 is predicted at 298 K, while the measured value is 0.0108). Although the accuracy of the model could be improved by treating the melting properties as adjustable parameters (as in other studies (26, 29, 34−38)), or by using some of these solubility data to refine the group parameters, we consider the current results satisfactory, and use the models presented to predict the solubility in other solvents and to study di- and tripeptides. We are interested in considering a fully predictive approach at this point and in assessing the merits of standard, neutral, groups to treat these solutions, neglecting in addition any speciation. Moreover, the uncertainty inherent in the measurement of the melting properties of amino acids and peptides (as discussed earlier) means that using solubility data to estimate molecular model parameters may lead to unexpected biasing of the molecular model developed.

**Figure 13:**
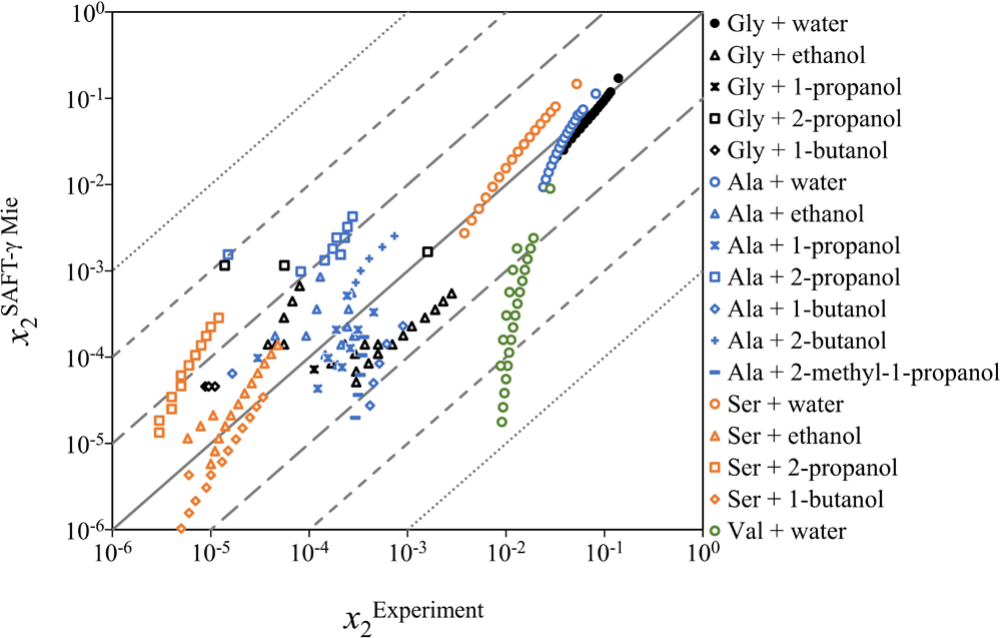
Parity plot of the solid−liquid equilibria (solubility) of glycine (Gly), alanine (Ala), serine (Ser), and valine (Val) in water, ethanol, 1-propanol, 1-butanol, 2-methyl-1-propanol, 2-propanol, and 2-butanol. The solid diagonal line denotes exact agreement between experiments and calculations whereas each pair of dashed and dotted lines denote a change in order of magnitude. Sources of the experimental data can be found in Table 6.

The predictive capability of the model is now assessed by calculating the solubilities of glycine, alanine, and serine, in various primary (ethanol,^18,22,155−163^ 1-propanol,^21,22,155,156,160^ 1-butanol,^23,155,156,162,164^ and 2-methyl-1-propanol^155^) and secondary alcohols (2-propanol^21,22,156,160,162,163,165^ and 2-butanol^155^), and in water + alcohol mixtures (water + ethanol and water + propanol^22^). The predictions are presented in Figure 13 as a parity plot against the experimental data available. The aqueous solubility calculations and data of Figure 12 are also included for completeness, and AADs for each of the systems considered are listed in Table 6. It is encouraging to see that most of the calculations are within an order of magnitude of the experimental data. Given the very low solubility values of some of the systems, these results confirm the predictive capability of the method and validate the use of neutral models as proposed here. It can be seen that the model performs best for the prediction of solubility in water, and that deviations increase as the magnitude of solubility becomes smaller, as is the case in alcohols. It is, however, worth noting that the solubility measurements of amino acids in alcohols reported in the literature vary significantly depending on the source. One clear example is the solubility of glycine in 2-propanol. The prediction is in very good agreement with the data reported by Bouchard et al.,^165^ but off by two orders of magnitude when compared to the data of Abraham et al.,^156^ even though in both studies, the solubilities were measured using the gravimetric method. A similar observation can be made for glycine in ethanol, alanine in ethanol, and alanine in 1-butanol.

**Table 6:** Overview of the Accuracy of SAFT-γ Mie in the Calculation of Solubility for Amino Acids in Water or Alcohol^a^

system	*T*/K	*N*^D^	AAD (*x*_1_^sat^)	figure	ref
glycine (1) + water (2)	273–373	36	1.0101 × 10^–2^	12, 13	(22,80,81,166)
glycine (1) + ethanol (2)	278–333	26	5.3488 × 10^–4^	13	(18,22,156−161)
glycine (1) + 1-propanol (2)	298	2	3.9995 × 10^–5^	13	(22,156,160)
glycine (1) + 2-propanol (2)	298–310	4	8.4938 × 10^–4^	13	(22,156,160,165)
glycine (1) + 1-butanol (2)	298	3	3.5925 × 10^–5^	13	(23,156,164)
alanine (1) + water (2)	273–373	29	8.0163 × 10^–3^	12, 13	(80,155)
alanine (1) + ethanol (2)	283–333	16	1.9809 × 10^–4^	13	(18,22,155,157,159,160)
alanine (1) + 1-propanol (2)	283–333	12	1.1201 × 10^–4^	13	(21,22,155,160)
alanine (1) + 2-propanol (2)	283–333	12	2.1200 × 10^–3^	13	(21,22,155,160)
alanine (1) + 1-butanol (2)	283–323	6	4.0025 × 10^–4^	13	(23,155)
alanine (1) + 2-butanol (2)	283–323	5	1.0442 × 10^–3^	13	(155)
alanine (1) + 2-methyl-1-propanol (2)	283–323	5	2.5037 × 10^–4^	13	(155)
serine (1) + water (2)	273–373	17	1.9293 × 10^–2^	12, 13	(80)
serine (1) + ethanol (2)	278–333	15	2.1964 × 10^–5^	13	(159,162)
serine (1) + 2-propanol (2)	278–333	12	9.3992 × 10^–5^	13	(162)
serine (1) + 1-butanol (2)	278–333	13	4.6741 × 10^–6^	13	(23,162)
valine (1) + water (2)	273–343	23	1.2640 × 10^–2^	12, 13	(80)
valine (1) + ethanol (2)	288–343	9	4.8847 × 10^–5^	12, 13	(159)
valine (1) + 2-propanol (2)	293–343	6	5.6699 × 10^–4^	12, 13	(163)

a *N*^D^ is the number of experimental data points used to calculate the average absolute deviation (AAD) in mole fraction.

**Figure 14:**
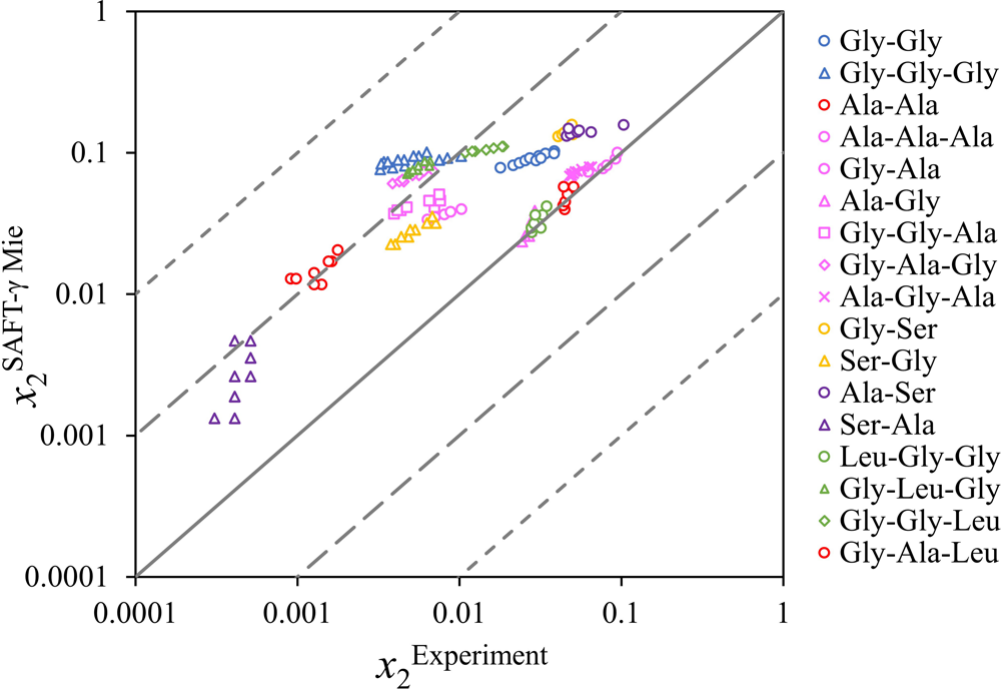
Parity plot of the solid−liquid equilibria (solubility) in water of di- and tripeptides made up of residues of the amino acids glycine (Gly), alanine (Ala), leucine (Leu), and serine (Ser). The solid diagonal line denotes exact agreement between experiments and calculations, whereas each pair of dashed lines denotes deviation of an increasing order of magnitude. Sources of the experimental data can be found in Table 7.

Having assessed the performance of the SAFT-γ Mie model for the prediction of the solubility of amino acids when treated as uncharged unspeciated mixtures, we consider now the prediction of solubility in peptide solutions. The predicted aqueous solubility of 17 di- and tripeptides containing glycine (Gly), alanine (Ala), leucine (Leu), and serine (Ser), is compared to experimental data as a parity plot in Figure 14 and Table 7. The corresponding melting temperatures and enthalpies of fusion of each of the peptides are listed in Table 5. We note that, as in the case of amino acid solubility, the solubility calculations for peptides are highly sensitive to uncertainty in the enthalpy of fusion, which unfortunately are reported with larger uncertainty ranges than those of the amino acids. We use the actual values reported in Do et al.^56,153^ in all of our calculations. We find that the most-accurate predictions are those for glycine homopeptides (Gly-Gly and Gly-Gly-Gly), and we find that with few exceptions, the predicted values are within an order of magnitude of the experimental values, which, given the very low solubility of these larger compounds, is a promising result. Furthermore, it is interesting to note that our model leads either to very good agreement with experiments or to an overprediction; in none of the cases are the solubilities underpredicted.

**Table 7:** Overview of the Accuracy of SAFT-γ Mie in the Calculation of Solubility for Peptides in Water^a^

solute	*T*/K	*N*^D^	AAD (*x*_1_^sat^)	ref
Gly-Gly	278–313	11	0.006086	(56,84)
Gly-Gly-Gly	278–313	16	0.04518	(56,84)
Ala-Ala	293–323	6	0.03935	(56)
Ala-Ala-Ala	288–308	5	0.006761	(56)
Gly-Ala	293–323	5	0.04532	(56)
Ala-Gly	293–323	6	0.01910	(56)
Gly-Gly-Ala	293–323	9	0.01151	(56)
Gly-Ala-Gly	293–323	8	0.02025	(56)
Ala-Gly Ala	288–308	9	0.02611	(56)
Leu-Gly-Gly	293–323	7	0.02242	(56)
Gly-Leu-Gly	293–323	9	0.01583	(56)
Gly-Gly-Leu	288–308	9	0.01603	(56)
Gly-Ala-Leu	293–323	9	0.0007906	(56)
Gly-Ser	293–323	6	0.04701	(56)
Ser-Gly	288–308	9	0.003343	(56)
Ala-Ser	293–323	7	0.03642	(56)
Ser-Ala	288–308	10	0.0002201	(56)

a *N*^D^ is the number of experimental data points used to calculate the average absolute deviation (AAD) in mole fraction.

Overall, the results presented suggest that treating amino acids and peptides as neutral species can lead to satisfactory predictions of solubility in water and alcohols. The assumption of neutrality is useful when modeling systems at the isoelectric point, without the need to account for the zwitterionic nature of the amino acid or the peptide and their speciation, thus reducing the number of species (and equilibrium relations) that need to be accounted for in the model. However, to model the behavior of amino acids or peptides at pH values different from the p*I*, it becomes essential to account explicitly for their zwitterionic nature. We consider these models in the following section.

## Solubility of Amino Acids as a Function of pH: Charged Models

The treatment of amino acids and peptides as neutral species limits the possibility of modeling pH-dependent solubility, which is an important property in pharmaceutical and biological applications. For example, the bioavailability of a pharmaceutical product depends sensitively on its solubility in the human body, within which there are significant variations in pH. Furthermore, the ability to model the speciation behavior of amino acids and peptides is essential to understanding their behavior in salt solutions, which plays a substantial role in screening solvent conditions.

Amino acids are ampholytes, meaning that they possess a dual acid–base nature conferred by the presence of the NH_2_ amino and COOH carboxyl groups, which ionize to NH_3_^+^ and COO^–^, respectively. Additionally, some amino acids contain ionizable side groups, rendering them polyprotic ampholytes. In our current work, only diprotic amino acids (containing nonionizable side groups) are considered. The speciation of polyprotic amino acids will be considered in future work.

We study aqueous solutions of glycine and alanine, as reliable solubility data as a function of pH are available for these.^167,168^ We implement SAFT-γ Mie models in which we treat the amino acids as zwitterions, i.e., molecules that carry an overall charge of zero but which lead to the formation of cationic and anionic species in solution as pH changes. We incorporate the solution of the SLE (solubility) as well as the chemical-equilibrium relations, accounting for speciation of the amino acid and the solvent in the liquid-phase mixture.

### Zwitterion, Cation, and Anion Models

**Figure 15:**
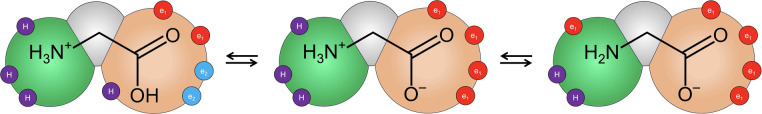
SAFT-γ Mie representation the glycine cation, zwitterion, and anion as modeled in the current work. A heteronuclear model of fused spherical segments is implemented in which short-range association sites are represented with smaller purple (sites of type H), red (type e_1_), and light blue (type e_2_) circles. The reactions also involve water and the hydronium and hydroxide ions, which are not shown here.

To model the speciation behavior and pH-dependent solubility of amino acids in water, the zwitterion, amino acid cation, and anion, as well as water, with the hydronium and hydroxide ions (which are products of water dissociation), and the counterions Na^+^ and Cl^–^ (which are the ionization products of the strong base NaOH and strong acid HCl), respectively, need to be taken into account. Our proposed SAFT-γ Mie model of glycine as a zwitterion, with its corresponding cation and anion, is shown in Figure 15. Modeling these species in solution requires characterizing the like and unlike interactions of the COO^–^, NH_3_^+^, H_3_O^+^, OH^–^, Na^+^, and Cl^–^ groups, in addition to the interactions of the relevant neutral groups. As shown in the parameter matrix of Figure 3, most of the interactions of charged groups have been presented in previous work. The hard-sphere diameter σ_*kk*_ and shape factor *S*_*k*_ of the charged groups are based on those of the corresponding uncharged group (they are assigned the same value). Additionally, a Born diameter is estimated by increasing the bare diameter of the ion by 7% (σ_*kk*_^Born^ = 1.07σ_*kk*_) to correct for the nonsphericity of the solvation ion cavity following the proposition of Rashin and Honig.^169^ Moreover, a corresponding charge is assigned (*Z*_*k*_ = + 1 for a cationic group, *Z*_*k*_ = −1 for an anionic group). To model the amino-acid zwitterion, the NH_3_^+^ and COO^–^ groups are used, but an overall charge of zero is assigned for the molecule (*Z*_*i*_ = 0), such that no Coulombic or Born contribution arises in the calculation of the free energy contribution of this species (so that these contributions equal zero for species with *Z*_*i*_ = 0). The dispersion energy of the charged groups is different from that of the corresponding neutral group, as can be expected, and is calculated as described in Wehbe et al.^47^ The number of association sites is also different from that of the neutral groups, reflecting the loss or gain of a proton and the different tendency to form hydrogen bonds. Furthermore, a number of the unlike interactions involving the charged groups are obtained using combining rules; this was shown to be reliable in a previous study of the solubility of ibuprofen,^47^ and accordingly, we follow the same strategy here. In the case of the unlike COO^–^–NH_3_^+^ interaction, however, we find that the association energy parameter (ε_*kl*,*ab*_^HB^) between the e_1_ site on the COO^–^ group and the H site on the NH_3_^+^ needs further refining to accurately capture the reported solubility of glycine at the isoelectric point.

The optimized and calculated like and unlike parameters of the SAFT-γ Mie groups relevant to the aqueous solutions of glycine, alanine, water, and the related ions that result from their speciation, are presented in Tables 1, 2, and 3.

### Solid–Liquid Equilibrium and Chemical Equilibria

To model the SLE (solubility) of diprotic amino acids, the solid–liquid equilibrium eq 25 is solved for the amino acid zwitterion, taking into account the speciation (chemical equilibrium) relations of the acid–base behavior of the amino acid and the ionization of water in the liquid phase. The concentrations of each of these species are determined as a function of changing pH, at given *T* and *P*. Thus, the liquid phase is a mixture containing the neutral zwitterion, the amino-acid cation and anion, the counterions of the acid and base, and the species related to water, in coexistence with a solid phase containing only the neutral amino acid.

For a diprotic amino acid (NH_3_^+^–RCH_2_–COO^–^), where R is a nonionizable side group in aqueous solution, the acid–base chemical equilibria between the different species of the amino acid can be written as
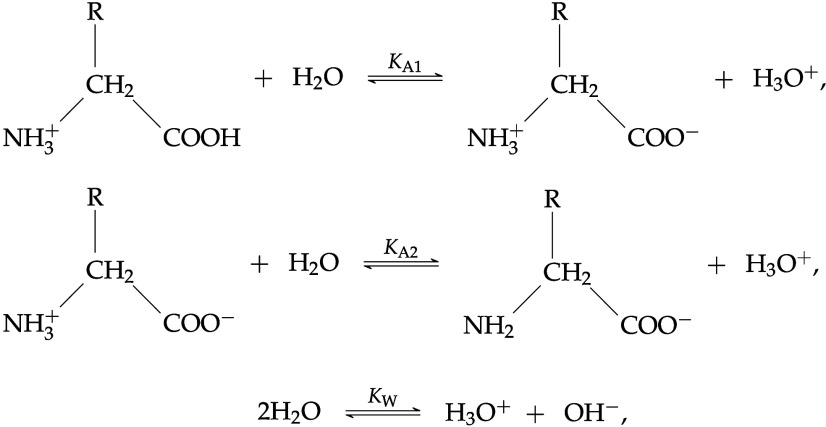
e1where *K*_*A*1_ and *K*_*A*2_ are the (true) equilibrium constants^170^ associated with the speciation of the zwitterion (AA^±^) and the cation (AA^+^) and anion (AA^–^) amino acids, respectively, and *K*_*W*_ is the dissociation constant of water. These are given as
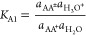
26
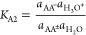
27and
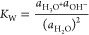
28where *a*_*i*_ is the activity of species *i*. The equilibrium constants are related to the corresponding p*K*_*i*_ by
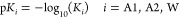
29and we calculate pH as^171^
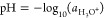
30The activity *a*_*i*_ is calculated following the asymmetric convention.
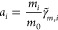
31at each pressure, temperature, and composition for all species except water. Here, *m*_*i*_ is the molality of *i*, *m*_0_ = 1 mol kg^–1^ is the reference molality, and γ̃_*m*,*i*_ is the asymmetric molality-based activity coefficient, calculated as
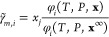
32where *x*_*j*_ is the mole fraction of the solvent (water), φ_*i*_ the fugacity coefficient of *i*, calculated using the SAFT-γ Mie approach, **x** the composition vector of the mixture, and **x**^∞^ the composition vector of the reference which is an infinitely dilute mixture. In our calculations, a mole fraction of 1 × 10^–15^ is used for the infinite-dilution fugacity coefficient. The water activity (*a*_w_) is calculated according to the symmetric convention as
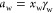
33where *x*_w_ is the mole fraction of water, and γ_w_ is the symmetric mole-fraction-based activity coefficient, calculated as
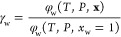
34

To account for the effect of temperature on the equilibrium constants, the van ’t Hoff equation is used:
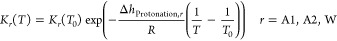
35where *K*_*r*_(*T*) is the equilibrium constant of reaction *r* at the system temperature, *K*_*r*_(*T*_0_) is the equilibrium constant at a known reference temperature (here *T*_0_ = 298.15 K), and *Δh*_Protonation,*r*_ is the enthalpy of protonation of reaction *r*. The values of the equilibrium constant for reactions involving glycine and alanine and the corresponding enthalpies of protonation can be found in Table 8. The temperature dependence of the water dissociation constant *K*_W_ is also calculated using eq 35 with *K*_W_(*T*_0_) = 1.0077 × 10^–14^ and *Δh*_Protonation,W_ = 56.149 kJ mol^−1^.^172,173^

**Table 8:** Acid–Base Equilibrium Properties of Glycine and Alanine

compound	*K*_A1_(298.15 K)	*Δh*_A1_/ (kJ mol^−1^)	*K*_A2_(298.15 K)	*Δh*_A2_/ (kJ mol^−1^)	ref
Glycine	4.525 × 10^–3^	61.101	2.54 × 10^–10^	44.780	(174)
Alanine	4.535 × 10^–3^	61.101	2.06 × 10^–10^	47.919	(174)

**Figure 16:**
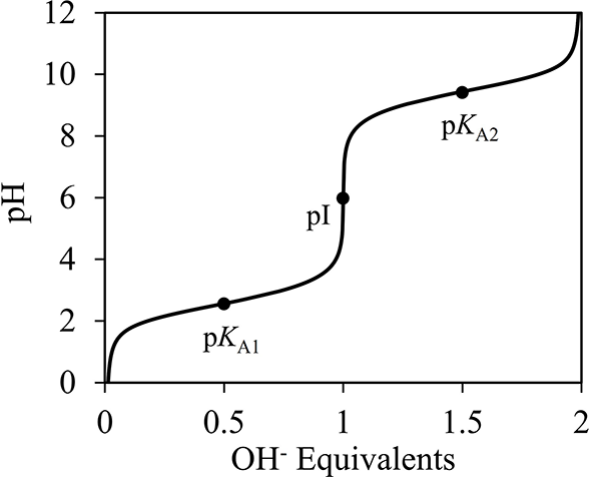
SAFT-γ Mie calculations of the acid–base titration curve for glycine at 298.15 K and 1 bar for an unsaturated glycine zwitterion molar composition of 0.00265. The OH^–^ equivalents are calculated as the proportion of OH^–^ molecules required to neutralize the glycine species, i.e., 50% of the cationic glycine is neutralized at p*K*_A1_ and 100% is neutralized at p*I*, at which glycine is predominantly in the zwitterionic form. At p*K*_A2_, 50% of the zwitterionic species is ionized into the anionic form. Close to pH 12, glycine is predominantly in the anionic form.

In Figure 16, we illustrate schematically the amphoteric speciation behavior of a diprotic amino acid, in this case glycine, at fixed *T* = 298.15 K, *P* = 1 bar, and zwitterion molar composition *x*_AA^±^,glycine_ = 0.00265. The zwitterion composition is fixed to a value known to be below the solid–liquid solubility to model an unsaturated solution of the amino acid fully in the liquid phase. The OH^–^ equivalents are calculated from the net charge of the amino acid species of *i*:
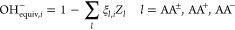
36where ξ_*l*,*i*_ are the relative concentrations of the amino-acid species *l*, given by
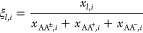
37This is in agreement with the definition of OH^–^ equivalents as the number of moles of OH^–^ ions required to convert 50% of the glycine cations to zwitterions (at the 0.5 equivalence point), to convert 100% of the glycine cations to zwitterions (at the 1.0 equivalence point), and to convert 50% of the glycine zwitterions to anions (at the 1.5 equivalence point). The glycine zwitterions are fully converted to anions at the 2.0 equivalence point.

In order to calculate the solubility of the amino acids incorporating the relevant speciation, for a given *T*, *P*, and pH, eqs 25–28 are solved simultaneously, alongside the equation for material conservation (∑_*i*=1_^*N*_C_^*x*_*i*_ = 1) and the equation for charge conservation (∑_*i*=1_^*N*_C_^*x*_*i*_*Z*_*i*_ = 0). In comparing with the experimental data, we note that solubility here is given as the sum of molalities of all the amino acid species in solution, i.e., (*m*_*AA*_ = *m*_AA^±^_ + *m*_AA^+^_ + *m*_AA^–^_), although only the chemical potential of the zwitterion is equated in the liquid and solid phase (the cation and anion are present only in solution).

**Figure 17:**
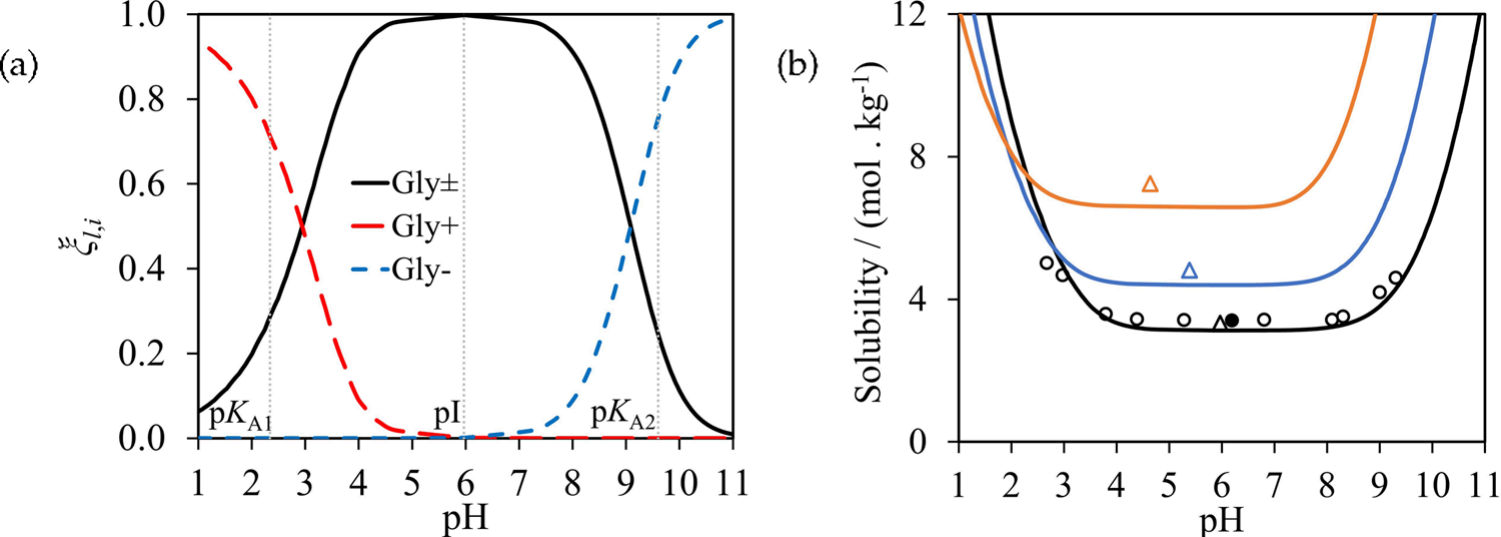
(a) Relative concentration (ξ_*l*,*i*_) of the glycine zwitterion (continuous black curve), the cation (long-dashed red curve), and the anion (short-dashed blue curve) at 298.15 K and 1 bar, as a function of pH. The p*K*_A_ and p*I* values^174^ are denoted by the vertical black dotted lines. (b) The solubility of glycine in water at 298.15 K (black), 318.15 K (blue), and 348.15 K (orange) and at 1 bar as a function of pH. The continuous curves represent SAFT-γ Mie calculations and symbols represent experimental solubility data; circles denote pH-dependent data of Needham et al.^167^ and triangles solubility data at the isoelectric point (p*I*) of Dalton and Schmidt.^80^ The filled circle represents the data point used in optimizing the NH_3_^+^–COO^–^ interaction, while empty symbols denote data not used in the parameter estimation.

In Figure 17 the calculated concentrations of the glycine zwitterion, anion, and cation and the overall SLE (solubility) are presented as a function of pH at 298.15 K and 1 bar. In Figure 17(a) the relative concentrations of the glycine zwitterion AA^±^, cation AA^+^, and anion AA^–^, given by eq 37, can be seen. The calculated solubility is compared to the experimental data available^80,168^ in Figure 17(b). In the calculations, the pH of the solution is varied by adding NaOH (for pH < pI) or HCl (for pH > pI), and both NaOH and HCl are modelled as fully dissociated into their respective ions. The equilibrium constants *K*_A1_ and *K*_A2_ are calculated using eq 35 with the parameters in Table 8, and *K*_W_ is calculated as described earlier.

As can be seen in the figure, for a significant range of pH close to the isoelectric point, the prevalent species in solution is the zwitterion, which has a lower water solubility than the cation and anion and hence leads to a solubility minimum (cf. Figure 17(b)). At low values of pH, the equilibrium tends toward the left-hand side of eq 26, and the glycine cation is the prevalent species in solution. This accumulation of positively charged ions is balanced by the presence of negatively charged counterions (e.g., Cl^–^ from HCl). At high values of pH, the equilibrium tends to the right-hand side of eq 27 resulting in the glycine anion becoming the prevalent species, which is now electrostatically balanced by a similar concentration of positively charged counterions (e.g., Na^+^ from NaOH). The cationic and anionic forms of glycine are highly soluble in water due to favorable solvation interactions between these ions and water molecules, leading to the higher solubility seen at both ends of the pH scale in the figure. The calculated solubility is in good agreement with the experimental data (Figure 17(b)), with only a small deviation noticeable at pH = 2.7. The influence of the temperature on the pH-dependent solubility can also be seen in Figure 17(b). At 348.15 K, the p*K*_A1_ and p*K*_A2_ values calculated using eqs 35 and 29 (0.807 and 8.47, respectively) are significantly lower than those at 298.15 K (2.34 and 9.60, respectively). This results in a shift in the pH-solubility profile to the left, centered around the p*I* value, which is calculated as the arithmetic mean of the p*K*_A1_ and p*K*_A2_ values. Additionally, at higher temperatures, the solubility of glycine is higher, in accordance with eq 25, which leads to an upward shift in the solubility minimum. This upward and leftward shift causes the two curves to cross in the low-pH region.

**Figure 18:**
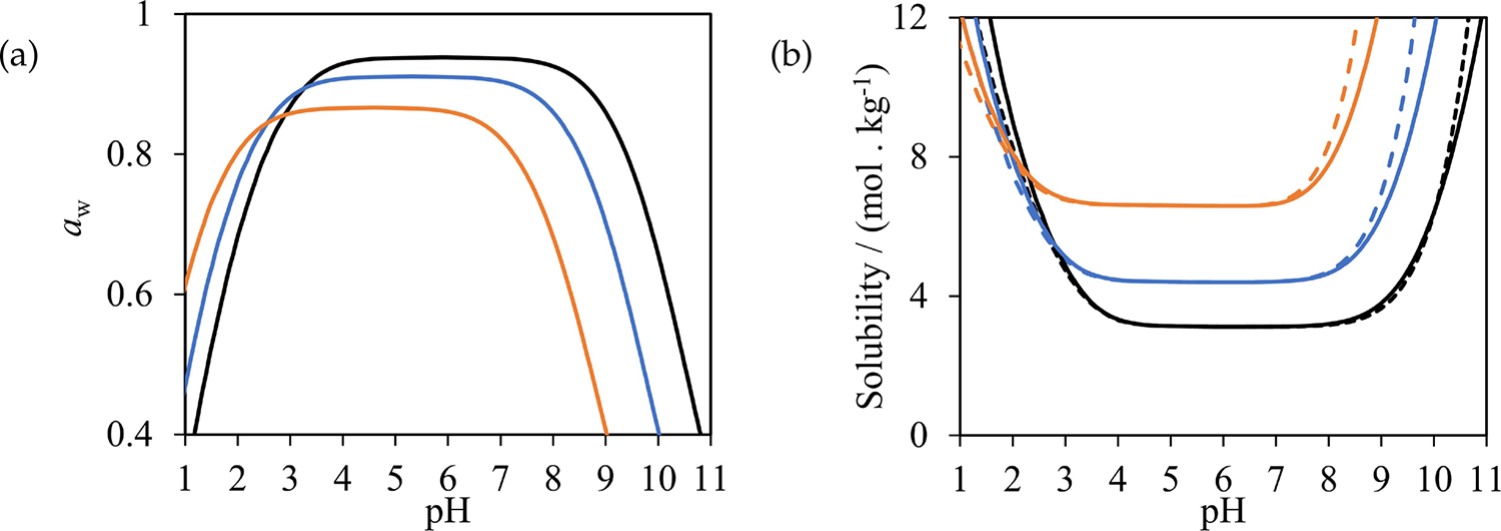
(a) The activity of water (*a*_w_) calculated using SAFT-γ Mie as a function of pH for the system containing glycine (described in Figure 17) at 298.15 K (black), 318.15 K (blue), and 348.15 K (orange) and at 1 bar. (b) The solubility of glycine in water at 298.15 K (black), 318.15 K (blue), and 348.15 K (orange) and at 1 bar as a function of pH. The solid curves represent calculations in which the real value of *a*_w_ is used and the dashed curves represent calculations in which *a*_w_ is taken to be unity.

It is common practice in modeling chemical equilibria in aqueous solutions to assume that the activity of water (*a*_w_) is equal to 1, especially in dilute solutions, and to neglect this contribution in the equilibrium-constant equations. While this can be a valid approximation near the isoelectric point (p*I*), at the high and low ends of the pH range, *a*_w_ is, however, no longer close to 1, and values below 0.5 can be found, as can be seen in Figure 18(a). At the pH extremes, the concentrations of the speciated amino acid and the counterions are high, reflecting their high solubility, and the solution cannot be considered to be close to the reference dilute molality of 1 mol kg^–1^ for these species. The impact of incorporating *a*_w_ in the chemical equilibrium equations on the solubility calculations, can be seen in Figure 18(b). As expected, including *a*_w_ in eqs 26–28 leads to a negligible change in the calculated solubility at the p*I*. However, in the low and high pH range, a more pronounced deviation between calculations that include *a*_w_ and those that take it to be unity can be seen. The calculations shown in Figure 17(b) are obtained with the inclusion of the actual activity of water throughout the range of thermodynamic conditions presented.

**Figure 19:**
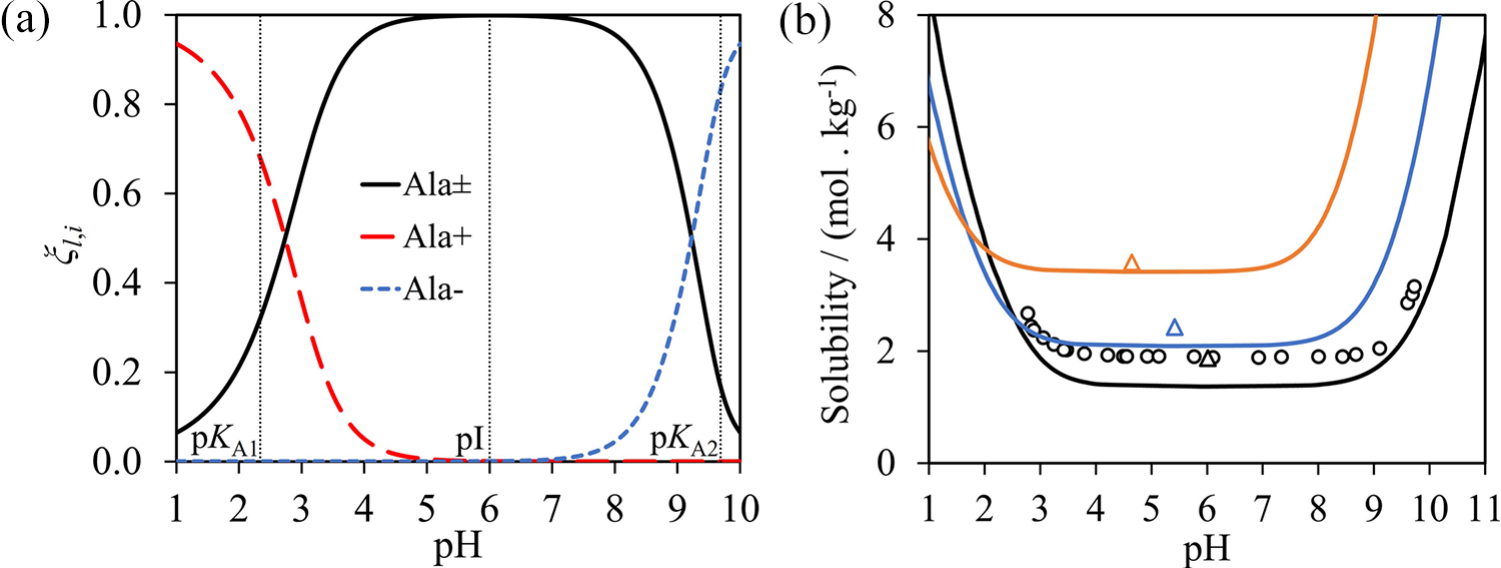
(a) Relative concentration (ξ_*l*,*i*_) of the alanine zwitterion (continuous black curve), the cation (long-dashed red curve), and the anion (short-dashed blue curve) at 298.15 K and 1 bar, as a function of pH. The p*K*_A_ and p*I* values^174^ are denoted by the vertical black dotted lines. (b) The solubility of alanine in water at 298.15 K (black), 318.15 K (blue), and 348.15 K (orange) and 1 bar, as a function of pH. The continuous curves represent SAFT-γ Mie predictions and the symbols the experimental solubility data. The circles denote pH-dependent solubility data of Tseng et al.,^168^ and triangles denote solubility data at the isoelectric point (p*I*) of Dalton and Schmidt.^80^

In order to assess the transferability of the NH_3_^+^−COO^–^ interaction, which is optimized using the solubility data of glycine as a function of pH, we now carry out predictive SAFT-γ Mie calculations for alanine in water (at 298.15 and 348.15 K and 1 bar) as a function of pH, for which no data are used for the parameter estimation. A comparison of our calculations and the available experimental data is shown in Figure 19. As in the case of glycine, the alanine zwitterion dominates over a wide range of pH around the isoelectric point, resulting in the minimum solubility shown in Figure 19(b). We note that these calculations are fully predictive, and that they show a good agreement with the experimental data of Tseng et al.^168^ and Dalton and Schmidt.^80^ The calculations exhibit a small underprediction of the solubility across the pH range at 298 K, but accurately capture the qualitative trend in the data as the pH changes. In these calculations, as discussed in the previous figure, the activity of water is included at each state (i.e., it is not assumed to be 1). The level of agreement with the experimental data available confirms the predictive ability of our modeling approach and the robustness of the SAFT-γ Mie model presented.

## Conclusions

Modeling the solubility of amino acids and peptides is of key relevance in biological and pharmaceutical systems and in pharmaceutical process development. It also poses an interesting challenge that involves the solution of phase and chemical equilibrium and requires detailed molecular models of charged and uncharged species in solution when pH effects are of interest. Here, the SAFT-γ Mie framework has been used to describe the solubility of amino acids and peptides in aqueous and alcohol solvents, over a range of conditions, including as a function of pH. Models treating the amino acids as neutral species (close to the isoelectric point) were considered first. The calculation of solubility with equations of state has been shown to be very sensitive to the value of the fusion enthalpy of the solid, a quantity that is unfortunately reported with large uncertainty for amino acids and peptides. Moreover, in the case of alcohol solvents, large deviations are observed in the measured solubilities. Despite these challenges, the predictions are found to be in good overall agreement with the data available for amino acids and di- and tripeptides. 283 amino acid solubility data points and 141 peptide solubility data points are considered, with average AADs in mole fraction of 0.0038 and 0.02128, respectively. The SAFT-γ Mie models presented have been developed using fluid-phase data (e.g., pure and mixture fluid-phase equilibrium data, and excess enthalpies), but not solid–liquid solubility data (with the exception of solubility data for glycine), which has been reserved as test data in order to minimize the impact of the uncertainty associated with the properties of the solids.

The SAFT-γ Mie treatment has been extended to include electrostatic contributions (ion–solvent and ion–ion) to the Helmholtz free energy to model and predict the solubility of amino acids as a function of pH, with an approach based on an effective spherical charged group deployed to handle the nonspherical nature of charged amino acids. The solid–liquid phase equilibrium condition is combined with the chemical equilibria associated with the speciation of amino acid zwitterions into the amino acid cation and anion, at variable pH. To model these systems, a number of group interactions have been developed as part of the current work. The parameter estimation has been carried out using experimental data of monofunctional compounds and mixtures where possible, with data involving amino acids only used to refine the COOH–NH_2_ and COO^–^–NH_3_^+^ interactions, for which a limited number of solubility data of glycine in water have been used. The transferability of the model parameters has been highlighted by presenting purely predictive calculations for conditions not included in the parameter estimation, hence demonstrating the validity of the predictive nature of the modeling approach. The model allows the study of the relative concentrations of charged and neutral species in solution for varying pH, and we have shown the impact of incorporating the true (activity-based) equilibrium constants, including that of water dissociation. This work paves the way for further studies involving ionic amino acids and larger peptides in varied solvents.

## Data Availability

Data underlying this article can be accessed on Zenodo at DOI: 10.5281/zenodo.14044894 and used under the Creative Commons Attribution license.

## Supporting Information Available

Expressions for the electrostatic contributions in Model M, as well the chemical potential and pressure expressions of Model G, and details of the derivatives of the Helmholtz free energy with respect to the screening length (PDF)

## References

[ref1] Banting F. G.; Best C. H.; Collip J. B.; Campbell W. R.; Fletcher A. A. Pancreatic extracts in the treatment of diabetes mellitus. Can. Med. Assoc. J. 1922, 12, 141–146. 20314060 PMC1524425

[ref2] Press O.W; Appelbaum F; Martin P.J; Matthews D.C; Bernstein I.D; Eary J.F; Nelp W.B; Gooley T; Glenn S; Porter B; Fisher D.R; et al. trial of 131I-B1 (anti-CD20) antibody therapy with autologous stem cell transplantation for relapsed B cell lymphomas. Lancet 1995, 346, 336–340. 10.1016/S0140-6736(95)92225-3. 7623531

[ref3] Goldenberg D. M.; DeLand F.; Kim E.; Bennett S.; Primus F. J.; van Nagell J. R. Jr; Estes N.; DeSimone P.; Rayburn P. Use of radiolabeled antibodies to carcinoembryonic antigen for the detection and localization of diverse cancers by external photoscanning. N. Engl. J. Med. 1978, 298, 1384–1388. 10.1056/NEJM197806222982503. 349387

[ref4] Lee A. C.-L.; Harris J. L.; Khanna K. K.; Hong J.-H. A comprehensive review on current advances in peptide drug development and design. Int. J. Mol. Sci. 2019, 20, 2383 10.3390/ijms20102383. 31091705 PMC6566176

[ref5] Matsson P.; Doak B. C.; Over B.; Kihlberg J. Cell permeability beyond the rule of 5. Adv. Drug Delivery Rev. 2016, 101, 42–61. 10.1016/j.addr.2016.03.013. 27067608

[ref6] Harris L. J.; Birch T. W. Zwitterions: Proof of the zwitterion constitution of the amino-acid molecule. II. Amino-acids, polypeptides, etc., and proteins as zwitterions, with instances of non-zwitterion ampholytes. Biochem. J. 1930, 24, 1080 10.1042/bj0241080. 16744434 PMC1254608

[ref7] Pinho S. P.; Silva C. M.; Macedo E. A. Solubility of amino acids: a group-contribution model involving phase and chemical equilibria. Ind. Eng. Chem. Res. 1994, 33, 1341–1347. 10.1021/ie00029a033.

[ref8] Carta R.; Tola G. Solubilities of L-cystine, L-tyrosine, L-leucine, and glycine in aqueous solutions at various pHs and NaCl concentrations. J. Chem. Eng. Data 1996, 41, 414–417. 10.1021/je9501853.

[ref9] Sano C.; Nagashima N.; Kawakita T.; Iitaka Y. Crystal and molecular structures of monosodium L-glutamate monohydrate. Anal. Sci. 1989, 5, 121–122. 10.2116/analsci.5.121.

[ref10] Guo H. M.; Liu H. W.; Wang Y. L.; Gao H. J.; Gong Y.; Jiang H. Y.; Wang W. Q. Surface structures of DL-valine and L-alanine crystals observed by atomic force microscopy at a molecular resolution. Surf. Sci. 2004, 552, 70–76. 10.1016/j.susc.2003.12.049.

[ref11] Liu Z.; Li C. Solvent-free crystallizations of amino acids: the effects of the hydrophilicity/hydrophobicity of side-chains. Biophys. Chem. 2008, 138, 115–119. 10.1016/j.bpc.2008.09.011. 18854245

[ref12] Marchese R.; Grandori R.; Carloni P.; Raugei S. On the zwitterionic nature of gas-phase peptides and protein ions. PLoS Comput. Biol. 2010, 6, e1000775 10.1371/journal.pcbi.1000775. 20463874 PMC2865515

[ref13] Patriksson A.; Marklund E.; van der Spoel D. Protein structures under electrospray conditions. Biochem. 2007, 46, 933–945. 10.1021/bi061182y. 17240977

[ref14] Patriksson A.; Adams C. M.; Kjeldsen F.; Zubarev R. A.; van der Spoel D. A direct comparison of protein structure in the gas and solution phase: The trp-cage. J. Phys. Chem. B 2007, 111, 13147–13150. 10.1021/jp709901t. 17973523

[ref15] Touboul D.; Jecklin M. C.; Zenobi R. Investigation of deprotonation reactions on globular and denatured proteins at atmospheric pressure by ESSI-MS. J. Am. Soc. Mass Spectrom. 2008, 19, 455–466. 10.1016/j.jasms.2007.12.011. 18276154

[ref16] Prakash H.; Mazumdar S. Direct correlation of the crystal structure of proteins with the maximum positive and negative charge states of gaseous protein ions produced by electrospray ionization. J. Am. Soc. Mass Spectrom. 2005, 16, 1409–1421. 10.1016/j.jasms.2005.04.002. 16006142

[ref17] Kirkwood J. G. Theory of solutions of molecules containing widely separated charges with special application to zwitterions. J. Chem. Phys. 1934, 2, 351–361. 10.1063/1.1749489.

[ref18] Cohn E. J.; McMeekin T. L.; Edsall J. T.; Weare J. H. Studies in the physical chemistry of amino acids, peptides and related substances. II. The solubility of α-amino acids in water and in alcohol-water mixtures. J. Am. Chem. Soc. 1934, 56, 2270–2282. 10.1021/ja01326a019.

[ref19] Chen C.-C.; Zhu Y.; Evans L. B. Phase partitioning of biomolecules: solubilities of amino acids. Biotechnol. Prog. 1989, 5, 111–118. 10.1002/btpr.5420050309.

[ref20] Pitzer K. S. Thermodynamics of electrolytes. I. Theoretical basis and general equations. J. Phys. Chem. 1973, 77, 268–277. 10.1021/j100621a026.

[ref21] Orella C. J.; Kirwan D. J. Correlation of amino acid solubilities in aqueous aliphatic alcohol solutions. Ind. Eng. Chem. Res. 1991, 30, 1040–1045. 10.1021/ie00053a028.

[ref22] Ferreira L. A.; Macedo E. A.; Pinho S. P. Solubility of amino acids and diglycine in aqueous-alkanol solutions. Chem. Eng. Sci. 2004, 59, 3117–3124. 10.1016/j.ces.2004.05.001.

[ref23] Gude M. T.; van der Wielen L. A. M.; Luyben K. C. A. M. Phase behavior of α-amino acids in multicomponent aqueous alkanol solutions. Fluid Ph. Equilib. 1996, 116, 110–117. 10.1016/0378-3812(95)02878-1.

[ref24] van Berlo M.; Gude M. T.; van der Wielen L. A.; Luyben K. C. A. Partition coefficients and solubilities of glycine in the ternary solvent system 1-butanol+ ethanol+ water. Ind. Eng. Chem. Res. 1997, 36, 2474–2482. 10.1021/ie960762w.

[ref25] Rudolph E. S. J.; Zomerdijk M.; Ottens M.; Van Der Wielen L. A. M. Solubilities and partition coefficients of semi-synthetic antibiotics in water+ 1-butanol systems. Ind. Eng. Chem. Res. 2001, 40, 398–406. 10.1021/ie000089h.

[ref26] Khoshkbarchi M. K.; Vera J. H. A simplified perturbed hard-sphere model for the activity coefficients of amino acids and peptides in aqueous solutions. Ind. Eng. Chem. Res. 1996, 35, 4319–4327. 10.1021/ie960076x.

[ref27] Khoshkbarchi M. K.; Vera J. H. Effect of NaCl and KCl on the solubility of amino acids in aqueous solutions at 298.2 K: measurements and modeling. Ind. Eng. Chem. Res. 1997, 36, 2445–2451. 10.1021/ie9606395.

[ref28] Soto A.; Arce A.; K. Khoshkbarchi M.; Vera J. H Effect of the cation and the anion of an electrolyte on the solubility of DL-aminobutyric acid in aqueous solutions: measurement and modelling. Biophys. Chem. 1998, 73, 77–83. 10.1016/S0301-4622(98)00139-2. 17029716

[ref29] Fuchs D.; Fischer J.; Tumakaka F.; Sadowski G. Solubility of amino acids: Influence of the pH value and the addition of alcoholic cosolvents on aqueous solubility. Ind. Eng. Chem. Res. 2006, 45, 6578–6584. 10.1021/ie0602097.

[ref30] Gross J.; Sadowski G. Perturbed-chain SAFT: An equation of state based on a perturbation theory for chain molecules. Ind. Eng. Chem. Res. 2001, 40, 1244–1260. 10.1021/ie0003887.

[ref31] Chapman W. G.; Gubbins K. E.; Jackson G.; Radosz M. SAFT: Equation of state solution model for associating fluids. Fluid Ph. Equilib. 1989, 52, 31–38. 10.1016/0378-3812(89)80308-5.

[ref32] Chapman W. G.; Gubbins K. E.; Jackson G.; Radosz M. New reference equation of state for associating liquids. Ind. Eng. Chem. Res. 1990, 29, 1709–1721. 10.1021/ie00104a021.

[ref33] Marrero J.; Gani R. Group-contribution based estimation of pure component properties. Fluid Ph. Equilib. 2001, 183, 183–208. 10.1016/S0378-3812(01)00431-9.

[ref34] Cameretti L. F.; Sadowski G. Modeling of aqueous amino acid and polypeptide solutions with PC-SAFT. Chem. Eng. Process. 2008, 47, 1018–1025. 10.1016/j.cep.2007.02.034.

[ref35] Ferreira L. A.; Breil M. P.; Pinho S. P.; Macedo E. A.; Mollerup J. M. Thermodynamic modeling of several aqueous alkanol solutions containing amino acids with the perturbed-chain statistical associated fluid theory equation of state. Ind. Eng. Chem. Res. 2009, 48, 5498–5505. 10.1021/ie801567w.

[ref36] Grosse Daldrup J.-B.; Held C.; Ruether F.; Schembecker G.; Sadowski G. Measurement and modeling solubility of aqueous multisolute amino-acid solutions. Ind. Eng. Chem. Res. 2010, 49, 1395–1401. 10.1021/ie900913c.

[ref37] Grosse Daldrup J.-B.; Held C.; Sadowski G.; Schembecker G. Modeling pH and solubilities in aqueous multisolute amino acid solutions. Ind. Eng. Chem. Res. 2011, 50, 3503–3509. 10.1021/ie1010367.

[ref38] Held C.; Cameretti L. F.; Sadowski G. Measuring and modeling activity coefficients in aqueous amino-acid solutions. Ind. Eng. Chem. Res. 2011, 50, 131–141. 10.1021/ie100088c.

[ref39] Wysoczanska K.; Nierhauve B.; Sadowski G.; Macedo E. A.; Held C. Solubility of DNP-amino acids and their partitioning in biodegradable ATPS: Experimental and ePC-SAFT modeling. Fluid Ph. Equilib. 2021, 527, 112830 10.1016/j.fluid.2020.112830.

[ref40] Aliyeva M.; Brandao P.; Gomes J. R.; Coutinho J. A.; Held C.; Ferreira O.; Pinho S. P. Salt effects on the solubility of aromatic and dicarboxylic amino acids in water. J. Chem. Thermodyn. 2023, 177, 106929 10.1016/j.jct.2022.106929.

[ref41] Papaioannou V.; Lafitte T.; Avendaño C.; Adjiman C. S.; Jackson G.; Müller E. A.; Galindo A. Group contribution methodology based on the statistical associating fluid theory for heteronuclear molecules formed from Mie segments. J. Chem. Phys. 2014, 140, 054107 10.1063/1.4851455. 24511922

[ref42] Dufal S.; Papaioannou V.; Sadeqzadeh M.; Pogiatzis T.; Chremos A.; Adjiman C. S.; Jackson G.; Galindo A. Prediction of thermodynamic properties and phase behavior of fluids and mixtures with the SAFT-γ Mie group-contribution equation of state. J. Chem. Eng. Data 2014, 59, 3272–3288. 10.1021/je500248h.

[ref43] Dufal S.; Lafitte T.; Haslam A. J.; Galindo A.; Clark G. N. I.; Vega C.; Jackson G. The A in SAFT: developing the contribution of association to the Helmholtz free energy within a Wertheim TPT1 treatment of generic Mie fluids. Mol. Phys. 2015, 113, 948–984. 10.1080/00268976.2015.1029027.

[ref44] Dufal S.; Lafitte T.; Haslam A. J.; Galindo A.; Clark G. N. I.; Vega C.; Jackson G. Corrigendum: the A in SAFT: developing the contribution of association to the Helmholtz free energy within a Wertheim TPT1 treatment of generic Mie fluids. Mol. Phys. 2018, 116, 283–285. 10.1080/00268976.2017.1402604.

[ref45] Sadeqzadeh M.; Papaioannou V.; Dufal S.; Adjiman C. S.; Jackson G.; Galindo A. The development of unlike induced association-site models to study the phase behaviour of aqueous mixtures comprising acetone, alkanes and alkyl carboxylic acids with the SAFT-γ Mie group contribution methodology. Fluid Ph. Equilib. 2016, 407, 39–57. 10.1016/j.fluid.2015.07.047.

[ref46] Samsatli S.; Staffell I.; Samsatli N. J. Optimal design and operation of integrated wind-hydrogen-electricity networks for decarbonising the domestic transport sector in Great Britain. Int. J. Hydrog. Energy 2016, 41, 447–475. 10.1016/j.ijhydene.2015.10.032.

[ref47] Wehbe M.; Haslam A. J.; Jackson G.; Galindo A. Phase behaviour and pH-solubility profile prediction of aqueous buffered solutions of ibuprofen and ketoprofen. Fluid Ph. Equilib. 2022, 560, 113504 10.1016/j.fluid.2022.113504.

[ref48] Haslam A. J.; González-Pérez A.; Di Lecce S.; Khalit S. H.; Perdomo F. A.; Kournopoulos S.; Kohns M.; Lindeboom T.; Wehbe M.; Febra S.; Jackson G.; Adjiman C. S.; Galindo A. Expanding the Applications of the SAFT-γ Mie Group-Contribution Equation of State: Prediction of Thermodynamic Properties and Phase Behavior of Mixtures. J. Chem. Eng. Data 2020, 65, 5862–5890. 10.1021/acs.jced.0c00746.

[ref49] Febra S. A.; Bernet T.; Mack C.; McGinty J.; Onyemelukwe I. I.; Urwin S. J.; Sefcik J.; ter Horst J. H.; Adjiman C. S.; Jackson G.; Galindo A. Extending the SAFT-γ Mie approach to model benzoic acid, diphenylamine, and mefenamic acid: Solubility prediction and experimental measurement. Fluid Ph. Equilib. 2021, 540, 113002 10.1016/j.fluid.2021.113002.

[ref50] Lazarou G. Development of the SAFT-γ Mie equation of state for predicting the thermodynamic behaviour of strong and weak electrolyte solutions. Ph.D. Thesis, Imperial College London, 2017.

[ref51] Di Lecce S.; Lazarou G.; Khalit S. H.; Adjiman C. S.; Jackson G.; Galindo A.; McQueen L. Modelling and prediction of the thermophysical properties of aqueous mixtures of choline geranate and geranic acid (CAGE) using SAFT-γ Mie. RSC Adv. 2019, 9, 38017–38031. 10.1039/C9RA07057E. 35541791 PMC9075776

[ref52] Di Lecce S.; Lazarou G.; Khalit S. H.; Pugh D.; Adjiman C. S.; Jackson G.; Galindo A.; McQueen L. Correction: Modelling and prediction of the thermophysical properties of aqueous mixtures of choline geranate and geranic acid (CAGE) using SAFT-γ Mie. RSC Adv. 2020, 10, 19463–19465. 10.1039/D0RA90058C. 35532400 PMC9054199

[ref53] Kohns M.; Lazarou G.; Kournopoulos S.; Forte E.; Perdomo F. A.; Jackson G.; Adjiman C. S.; Galindo A. Predictive models for the phase behaviour and solution properties of weak electrolytes: nitric, sulphuric, and carbonic acids. Phys. Chem. Chem. Phys. 2020, 22, 15248–15269. 10.1039/C9CP06795G. 32609107

[ref54] Lafitte T.; Apostolakou A.; Avendaño C.; Galindo A.; Adjiman C. S.; Müller E. A.; Jackson G. Accurate statistical associating fluid theory for chain molecules formed from Mie segments. J. Chem. Phys. 2013, 139, 154504 10.1063/1.4819786. 24160524

[ref55] Do H. T.; Chua Y. Z.; Kumar A.; Pabsch D.; Hallermann M.; Zaitsau D.; Schick C.; Held C. Melting properties of amino acids and their solubility in water. RSC Adv. 2020, 10, 44205–44215. 10.1039/D0RA08947H. 35517171 PMC9058464

[ref56] Do H. T.; Chua Y. Z.; Habicht J.; Klinksiek M.; Volpert S.; Hallermann M.; Thome M.; Pabsch D.; Zaitsau D.; Schick C.; et al. Melting properties of peptides and their solubility in water. Part 2: Di-and tripeptides based on glycine, alanine, leucine, proline, and serine. Ind. Eng. Chem. Res. 2021, 60, 4693–4704. 10.1021/acs.iecr.0c05652.

[ref57] Lee L. L. Molecular Thermodynamics of Nonideal Fluids; Butterworth-Heinemann, 2016.

[ref58] Barker J. A.; Henderson D. What is “liquid”? Understanding the states of matter. Rev. Mod. Phys. 1976, 48, 587 10.1103/RevModPhys.48.587.

[ref59] Barker J. A.; Henderson D. Perturbation theory and equation of state for fluids. II. A successful theory of liquids. J. Chem. Phys. 1967, 47, 4714–4721. 10.1063/1.1701689.

[ref60] Wertheim M. S. Thermodynamic perturbation theory of polymerization. J. Chem. Phys. 1987, 87, 7323–7331. 10.1063/1.453326.

[ref61] Chapman W. G.; Jackson G.; Gubbins K. E. Phase equilibria of associating fluids: chain molecules with multiple bonding sites. Mol. Phys. 1988, 65, 1057–1079. 10.1080/00268978800101601.

[ref62] Blum L. Mean spherical model for asymmetric electrolytes: I. Method of solution. Mol. Phys. 1975, 30, 1529–1535. 10.1080/00268977500103051.

[ref63] Blum L.; Hoye J. S. Mean spherical model for asymmetric electrolytes. 2. Thermodynamic properties and the pair correlation function. J. Phys. Chem. 1977, 81, 1311–1316. 10.1021/j100528a019.

[ref64] Born M. Volumen und hydratationswärme der ionen. Z. Phys. 1920, 1, 45–48. 10.1007/BF01881023.

[ref65] Eriksen D. K.; Lazarou G.; Galindo A.; Jackson G.; Adjiman C. S.; Haslam A. J. Development of intermolecular potential models for electrolyte solutions using an electrolyte SAFT-VR Mie equation of state. Mol. Phys. 2016, 114, 2724–2749. 10.1080/00268976.2016.1236221.

[ref66] Kournopoulos S.; Santos M. S.; Ravipati S.; Haslam A. J.; Jackson G.; Economou I. G.; Galindo A. The contribution of the ion-ion and ion-solvent interactions in a molecular thermodynamic treatment of electrolyte solutions. J. Phys. Chem. B 2022, 126, 9821–9839. 10.1021/acs.jpcb.2c03915. 36395498 PMC9720728

[ref67] Selam M. A.; Economou I. G.; Castier M. A thermodynamic model for strong aqueous electrolytes based on the eSAFT-VR Mie equation of state. Fluid Ph. Equilib. 2018, 464, 47–63. 10.1016/j.fluid.2018.02.018.

[ref68] Debye P.; Huckel E. Zur theorie der electrolyte. Phys. Z. 1923, 185–206.

[ref69] Maribo-Mogensen B.; Thomsen K.; Kontogeorgis G. M. An electrolyte CPA equation of state for mixed solvent electrolytes. AIChE J. 2015, 61, 2933–2950. 10.1002/aic.14829.

[ref70] Soave G. Equibrium constants from a modified Redlich-Kwong equation of state. Chem. Eng. Sci. 1972, 27, 1197–1203. 10.1016/0009-2509(72)80096-4.

[ref71] Huang S. H.; Radosz M. Equation of state for small, large, polydisperse, and associating molecules. Ind. Eng. Chem. Res. 1990, 29, 2284–2294. 10.1021/ie00107a014.

[ref72] Held C.; Reschke T.; Müller R.; Kunz W.; Sadowski G. Measuring and modeling aqueous electrolyte/amino-acid solutions with ePC-SAFT. J. Chem. Thermodyn. 2014, 68, 1–12. 10.1016/j.jct.2013.08.018.

[ref73] Schreckenberg J. M. A.; Dufal S.; Haslam A. J.; Adjiman C. S.; Jackson G.; Galindo A. Modelling of the thermodynamic and solvation properties of electrolyte solutions with the statistical associating fluid theory for potentials of variable range. Mol. Phys. 2014, 112, 2339–2364. 10.1080/00268976.2014.910316.

[ref74] Bernet T.; Wehbe M.; Febra S. A.; Haslam A. J.; Adjiman C. S.; Jackson G.; Galindo A. Modeling the Thermodynamic Properties of Saturated Lactones in Nonideal Mixtures with the SAFT-γ Mie Approach. J. Chem. Eng. Data 2024, 69, 650–678. 10.1021/acs.jced.3c00358. 38352073 PMC10859965

[ref75] Perdomo F. A.; Khalit S. H.; Adjiman C. S.; Galindo A.; Jackson G. Description of the thermodynamic properties and fluid-phase behavior of aqueous solutions of linear, branched, and cyclic amines. AIChE J. 2021, 67, e17194 10.1002/aic.17194.

[ref76] Hutacharoen P.; Dufal S.; Papaioannou V.; Shanker R. M.; Adjiman C. S.; Jackson G.; Galindo A. Predicting the solvation of organic compounds in aqueous environments: from alkanes and alcohols to pharmaceuticals. Ind. Eng. Chem. Res. 2017, 56, 10856–10876. 10.1021/acs.iecr.7b00899.

[ref77] Hutacharoen P. Prediction of partition coefficients and solubilities of active pharmaceutical ingredients with the SAFT-γ Mie group-contribution approach. Ph.D. Thesis, Imperial College London, 2017.

[ref78] Nur Jazlan N. R. Modelling the free energy of solvation: from data-driven to statistical mechanical approaches. Ph.D. Thesis, Imperial College London, 2020.

[ref79] Perdomo F. A.; Khalit S. H.; Graham E. J.; Tzirakis F.; Papadopoulos A. I.; Tsivintzelis I.; Seferlis P.; Adjiman C. S.; Jackson G.; Galindo A. A predictive group-contribution framework for the thermodynamic modelling of CO_2_ absorption in cyclic amines, alkyl polyamines, alkanolamines and phase-change amines: New data and SAFT-γ Mie parameters. Fluid Ph. Equilib. 2023, 566, 113635 10.1016/j.fluid.2022.113635.

[ref80] Dalton J. B.; Schmidt C. L. A. The solubilities of certain amino acids in water, the densities of their solutions at twenty-five degrees, and the calculated heats of solution and partial molal volumes. J. Biol. Chem. 1933, 103, 549–578. 10.1016/S0021-9258(18)75835-3.

[ref81] Yi Y.; Hatziavramidis D.; Myerson A. S.; Waldo M.; Beylin V. G.; Mustakis J. Development of a small-scale automated solubility measurement apparatus. Ind. Eng. Chem. Res. 2005, 44, 5427–5433. 10.1021/ie049215y.

[ref82] Kuramochi H.; Noritomi H.; Hoshino D.; Nagahama K. Measurements of vapor pressures of aqueous amino acid solutions and determination of activity coefficients of amino acids. J. Chem. Eng. Data 1997, 42, 470–474. 10.1021/je960113r.

[ref83] Romero C. M.; Cadena J. C. Effect of temperature on the volumetric properties of α, ω-amino acids in dilute aqueous solutions. J. Solution Chem. 2010, 39, 1474–1483. 10.1007/s10953-010-9602-1.

[ref84] Guo M.; Chang Z. H.; Liang E.; Mitchell H.; Zhou L.; Yin Q.; Guinn E. J.; Heng J. Y. The effect of chain length and side chains on the solubility of peptides in water from 278.15 to 313.15 K: A case study in glycine homopeptides and dipeptides. J. Mol. Liq. 2022, 352, 118681 10.1016/j.molliq.2022.118681.

[ref85] Pradhan S. D. The chain length and isomeric effect of alcohol on the excess properties of amine-alcohol systems: Excess free energy of mixing, enthalpy of mixing and volume change on mixing. Proceedings of the Indian Academy of Sciences-Chemical Sciences 1981, 90, 261–273. 10.1007/BF02879399.

[ref86] Domínguez M.; Martín S.; Artigas H.; López M. C.; Royo F. M. Isobaric Vapor-Liquid Equilibrium for the Binary Mixtures (2-Butanol+ n-Hexane) and (2-Butanol+ 1-Butylamine) and for the Ternary System (2-Butanol+ n-Hexane+ 1-Butylamine) at 101.3 kPa. J. Chem. Eng. Data 2002, 47, 405–410. 10.1021/je0101707.

[ref87] Thacker R.; Rowlinson J. S. The physical properties of some polar solutions. Part 1.–Volumes and heats of mixing. Trans. Faraday Soc. 1954, 50, 1036–1042. 10.1039/TF9545001036.

[ref88] Kimura T.; Suzuki T.; Takata K.; Soga A.; Nomoto Y.; Kamiyama T.; Nakai Y.; Matsui H.; Fujisawa M. Excess enthalpies of binary mixtures of butylamines + propanols at 298.15 K. J. Therm. Anal. Calorim. 2013, 113, 1467–1474. 10.1007/s10973-013-3226-9.

[ref89] Iloukhani H.; Soleimani M. Measurement and modeling the excess molar volumes and refractive index deviations of binary mixtures of 2-Propanol, 2-butanol and 2-pentanol with N-propylamine. J. Solution Chem. 2017, 46, 2135–2158. 10.1007/s10953-017-0683-y.

[ref90] Dominguez M.; Artigas H.; Cea P.; Lopez M. C.; Urieta J. S. Speed of sound and isentropic compressibility of the ternary mixture (2-butanol + n-hexane + 1-butylamine) and the constituent binary mixtures at 298.15 and 313.15 K. J. Mol. Liq. 2000, 88, 243–258. 10.1016/S0167-7322(00)00143-4.

[ref91] Weng W.-L.; Chen J.-T. Density and viscosity measurement of n-butylamine with hexyl alcohol isomer binary systems. J. Chem. Eng. Data 2004, 49, 1748–1751. 10.1021/je0498053.

[ref92] Amer Amezaga S. Vapor-liquid equilibrium at 760 mm of binary systems formed by methyl, ethyl, propyl, isopropyl, butyl, isobutyl, sec-butyl, and tert-butyl alcohols with propionic acid. Ann. Quím. 1975, 71, 117–126.

[ref93] Iwarere S. A.; Raal J. D.; Naidoo P.; Ramjugernath D. Vapour–liquid equilibrium of carboxylic acid–alcohol binary systems: 2-Propanol + butyric acid, 2-butanol + butyric acid and 2-methyl-1-propanol + butyric acid. Fluid Ph. Equilib. 2014, 380, 18–27. 10.1016/j.fluid.2014.07.025.

[ref94] Behroozi M.; Zarei H. Volumetric properties of highly nonideal binary mixtures containing ethanoic acid and propanoic acid with butan-2-ol, methyl-2-propanol, and 2-methyl-2-butanol at different temperatures. J. Chem. Eng. Data 2012, 57, 1089–1094. 10.1021/je201102x.

[ref95] Osborn A. G.; Douslin D. R. Vapor pressure relations of 13 nitrogen compounds related to petroleum. J. Chem. Eng. Data 1968, 13, 534–537. 10.1021/je60039a024.

[ref96] Chiali-Baba Ahmed N.; Negadi L.; Mokbel I.; Kaci A. A.; Jose J. Experimental determination of the isothermal (vapour + liquid) equilibria of binary aqueous solutions of sec-butylamine and cyclohexylamine at several temperatures. J. Chem. Thermodyn. 2012, 44, 116–120. 10.1016/j.jct.2011.08.009.

[ref97] Osborn A. G.; Scott D. W. Vapor pressures of 17 miscellaneous organic compounds. J. Chem. Thermodyn. 1980, 12, 429–438. 10.1016/0021-9614(80)90056-7.

[ref98] Simon A.; Huter J. Zur Kenntnis der Dampfdruckkurven, Schmelzpunkte und der chemischen Konstanten von Dimethyl-, Trimethyl-und Isobutyl-Amin. Z. Elektrochem. Angew. Phys. Chem. 1935, 41, 28–33. 10.1002/bbpc.19350410109.

[ref99] Shirai M. Dielectric Polarization of Some Aliphatic Amines in the Liquid State. Bull. Chem. Soc. Jpn. 1956, 29, 518–521. 10.1246/bcsj.29.518.

[ref100] Tôrres R. B.; Hoga H. E. Volumetric properties of binary mixtures of dichloromethane and amines at several temperatures and p = 0.1 MPa. J. Mol. Liq. 2008, 143, 17–22. 10.1016/j.molliq.2008.04.007.

[ref101] Saleh M. A.; Akhtar S.; Khan A. R. Excess molar volumes of aqueous solutions of butylamine isomers. Phys. Chem. Liq. 2000, 38, 137–149. 10.1080/00319100008045303.

[ref102] De Loos T. W.; Tijsseling H. R.; De Swaan Arons J. Vapor-liquid equilibria of the system ethane + 2-aminopropane. J. Chem. Eng. Data 1987, 32, 374–377. 10.1021/je00049a027.

[ref103] Wolff H.; Shadiakhy A. The vapour-pressure behaviour and the association of isomeric propylamines and n-deuteriopropylamines in mixtures with n-hexane. Fluid Ph. Equilib. 1983, 11, 267–287. 10.1016/0378-3812(83)85029-8.

[ref104] Pradhan S. D.; Mathur H. B. Thermodynamic study of binary mixtures of isomeric butylamines with n-hexane: Enthalpy of hydrogen bonding. Proc. Indian Acad. Sci. 1978, 87, 23–29. 10.1007/BF03182111.

[ref105] Matteoli E.; Lepori L.; Spanedda A. Thermodynamic study of heptane+ amine mixtures: I. Excess and solvation enthalpies at 298.15 K. Fluid Ph. Equilib. 2003, 212, 41–52. 10.1016/S0378-3812(03)00260-7.

[ref106] Brunner E. Löslichkeit von Wasserstoff in Aminen. Ber. Bunsenges. Phys. Chem. 1978, 82, 798–805. 10.1002/bbpc.19780820807.

[ref107] Payne K. Modelling the Carbonyl Group in 2-Ketones using the SAFT-γ Mie Methodology. M.Sc. Thesis, Imperial College London, 2017.

[ref108] McMurry J. In Organic Chemistry, 2nd ed.;Brooks/Cole Publishing Company: Pacific Grove, CA, 1988; section 25.4.

[ref109] Schmelzer J.; Pusch J. Phase equilibria in binary systems containing N-monosubstituted amides and hydrocarbons. Fluid Ph. Equilib. 1995, 110, 183–196. 10.1016/0378-3812(95)02753-2.

[ref110] Zaitseva K. V.; Varfolomeev M. A.; Verevkin S. P. Vapour pressures and enthalpies of vaporisation of N-alkyl acetamides. J. Mol. Liq. 2019, 293, 111453 10.1016/j.molliq.2019.111453.

[ref111] Hahn J.; Stoeck S. Determination of the vapor pressure of N-butylpropionamide and *N*-propylacetamide. Leuna-Protokoll 1983, 7271.

[ref112] Gertler S. I. Screening Tests of some N-Substituted Acetamides as Insecticides and Acarides; Agricultural Research Service, 1955; pp 33–14.

[ref113] Eshghi H.; Shafieyoon P. P_2_O_5_/SiO_2_ as a mild and efficient reagent for acylation of alcohols, phenols and amines under solvent-free conditions. J. Chem. Res. 2004, 2004, 802–805. 10.3184/0308234043431267.

[ref114] Mueller G.; Moerke K. Determination of the vapor-liquid equilibrium in the system trichloroethene-*N*-pentylacetamide. Leuna-Protokoll 1988, 4131.

[ref115] Gopal R.; Rizvi S. A. Vapour pressures of some mono-and di-alkyl substituted aliphatic amides at different temperatures. J. Indian Chem. Soc. 1968, 45, 13.

[ref116] Hahn J.; Moerke K. Vapor pressures of some caprolactam impurities. Leuna-Protokoll 1984, 4121.

[ref117] Gmehling J.; Krafczyk J.; Ahlers J.; Nebig S.; Hunecker I.; Eisel M.; Fischer D.; Krentscher B.; Beyer K. Pure compound data from DDB. DDB 1983, 2014.

[ref118] Mukesh B.; Sekhar M. C.; Reddy K. C. S.; Sreekanth T. Thermodynamic, DFT and molecular dynamics studies of intermolecular interactions between 2-methoxyaniline and N-substituted amide mixtures. Chem. Data Coll 2019, 22, 100241 10.1016/j.cdc.2019.100241.

[ref119] Jović B.; Nikolić A.; Kordić B. Densitometric and spectroscopic investigation of interactions of selected N-substituted amides and acetonitrile. J. Mol. Liq. 2014, 191, 10–15. 10.1016/j.molliq.2013.11.016.

[ref120] Gopal R.; Rizvi S. A. Physical properties of some mono-and dialkyl-substituted amides at different temperatures. J. Indian Chem. Soc. 1966, 43, 179–182.

[ref121] Millero F. J. Relative viscosity and apparent molal volume of N-methylpropionamide solutions at various temperatures. J. Phys. Chem. 1968, 72, 3209–3214. 10.1021/j100855a021.

[ref122] Hoover T. B. Conductance of potassium chloride in highly purified N-methylpropionamide from 20 to 40°. J. Phys. Chem. 1964, 68, 876–879. 10.1021/j100786a030.

[ref123] Hoover T. B. The N-Methylpropionamide-water system. Densities and dielectric constants at 20–40°. J. Phys. Chem. 1969, 73, 57–61. 10.1021/j100721a010.

[ref124] Van Evercooren J. E.; Merken G. V.; Thun H. P. The Conductivity of Hydrochloric Acid in N-Methylpropionamide at Temperatures from 15 to 50 °C. Bull. Soc. Chim. Belg. 1975, 84, 533–539. 10.1002/bscb.19750840603.

[ref125] Dawson L. R.; Graves R. H.; Sears P. G. Solvents Having High Dielectric Constants. III. Solutions of Sodium and Potassium Halides in N-Methylpropionamide and in N-Methylbutyramide from 30 to 60°. J. Am. Chem. Soc. 1957, 79, 298–300. 10.1021/ja01559a014.

[ref126] de Haan A.; Fischer K.; Haacke M.; Aufderhaar O.; Petri M.; Gmehling J. Vapor-liquid equilibria and enthalpies of mixing for binary mixtures of N-methylacetamide with aniline, decane, ethylene glycol, naphthalene, phenol, and water. J. Chem. Eng. Data 1997, 42, 875–881. 10.1021/je970031i.

[ref127] de Haan A. B.; Heine A.; Fischer K.; Gmehling J. Vapor-Liquid Equilibria and Excess Enthalpies for Octane + N-Methylacetamide, Cyclooctane + N-Methylacetamide, and Octane + Acetic Anhydride at 125 °C. J. Chem. Eng. Data 1995, 40, 1228–1232. 10.1021/je00022a018.

[ref128] Kortüm G.; Biedersee H. Dampf/Flüssigkeit-Gleichgewichte (Siedediagramme) binärer Systeme hoher relativer Flüchtigkeit. Wasser/N-Methylacetamid, Wasser/N-Methylformamid und N-Methylformamid/N-Methylacetamid. Chem. Ing. Technol. 1970, 42, 552–560. 10.1002/cite.330420810.

[ref129] Štejfa V.; Chun S.; Pokornỳ V.; Fulem M.; Růžička K. Thermodynamic study of acetamides. J. Mol. Liq. 2020, 319, 114019 10.1016/j.molliq.2020.114019.

[ref130] Nain A. K. Densities and volumetric properties of (acetonitrile + an amide) binary mixtures at temperatures between 293.15 and 318.15 K. J. Chem. Thermodyn. 2006, 38, 1362–1370. 10.1016/j.jct.2006.01.015.

[ref131] Pacak P. Refractivity and density of some organic solvents. Chem. Pap. 1991, 45, 29.

[ref132] Assarsson P.; Eirich F. R. Properties of amides in aqueous solution. I. Viscosity and density changes of amide-water systems. An analysis of volume deficiencies of mixtures based on molecular size differences (mixing of hard spheres). J. Phys. Chem. 1968, 72, 2710–2719. 10.1021/j100854a004.

[ref133] Casteel J. F.; Amis E. S. Conductance of sodium perchlorate in water-N-methylacetamide (NMA) solvent system. J. Chem. Eng. Data 1974, 19, 121–128. 10.1021/je60061a020.

[ref134] de Haan A. B.; Gmehling J. Excess enthalpies for various binary mixtures with N-methylacetamide or acetic anhydride. J. Chem. Eng. Data 1996, 41, 474–478. 10.1021/je950294h.

[ref135] Zaichikov A. M. Enthalpies of mixing of water with secondary amides of carboxylic acids. Russ. J. Gen. Chem. 1997, 67, 1355–1360.

[ref136] Zielkiewicz J. Vapour + liquid equilibrium measurements and correlation of the ternary mixture (N-methylacetamide + ethanol + water) at the temperature 313.15 K. J. Chem. Thermodyn. 2000, 32, 55–62. 10.1006/jcht.1999.0570.

[ref137] Manczinger J.; Kortüm G. Thermodynamische Mischungseffekte im System Wasser (1)/N-Methylacetamid (2). Zeitsch. Phys. Chem. Neue Folge 1975, 95, 177–186. 10.1524/zpch.1975.95.4-6.177.

[ref138] Egorov G. I.; Makarov D. M. Densities and Volumetric Properties of Aqueous Solutions of {water (1) + N-methylurea (2)} Mixtures at Temperatures of 274.15–333.15 K and at Pressures up to 100 MPa. J. Chem. Eng. Data 2017, 62, 4383–4394. 10.1021/acs.jced.7b00750.

[ref139] Saleh J. M.; Al-Azzawi L. H. Determination of the transfer energies of hydrobromic acid in *N*-methylurea + water mixtures at different temperatures. Iraqi J. Sci. 1980, 21, 507–525.

[ref140] Krakowiak J.; Wawer J.; Panuszko A. Densimetric and ultrasonic characterization of urea and its derivatives in water. J. Chem. Thermodyn. 2013, 58, 211–220. 10.1016/j.jct.2012.11.007.

[ref141] Lapanje S.; Vlachy V.; Kranjc Z.; Zerovnik E. Effect of temperature on the apparent molal volume of ethylurea in aqueous solutions. J. Chem. Eng. Data 1985, 30, 29–32. 10.1021/je00039a010.

[ref142] Chang W.; Wan H.; Guan G.; Yao H. Isobaric vapor-liquid equilibria for water + acetic acid + (N-methyl pyrrolidone or N-methyl acetamide). Fluid Ph. Equilib. 2006, 242, 204–209. 10.1016/j.fluid.2006.02.002.

[ref143] Singh M. Determination of Densities of Amino Compounds for Molar Volumes in Aqueous Solutions with Magnetic Float Densimeter at Various Temperatures. Biol. Sci.-PJSIR 2006, 49, 160–169.

[ref144] Hoerr C. W.; Balston A. W. The solubilities of the normal saturated fatty acids. J. Org. Chem. 1944, 09, 329–337. 10.1021/jo01186a005. 20280727

[ref145] Cepeda E. A.; Bravo R.; Calvo B. Solubilities of Lauric Acid in n-Hexane, Acetone, Propanol, 2-Propanol, 1-Bromopropane, and Trichloroethylene from (279.0 to 315.3) K. J. Chem. Eng. Data 2009, 54, 1371–1374. 10.1021/je800739y.

[ref146] Gonçalves Bonassoli A. B.; Oliveira G.; Bordón Sosa F. H.; Rolemberg M. P.; Mota M. A.; Basso R. C.; Igarashi-Mafra L.; Mafra M. R. Solubility Measurement of Lauric, Palmitic, and Stearic Acids in Ethanol, n-Propanol, and 2-Propanol Using Differential Scanning Calorimetry. J. Chem. Eng. Data 2019, 64, 2084–2092. 10.1021/acs.jced.8b01044.

[ref147] Calvo B.; Collado I.; Cepeda E. A. Solubilities of Palmitic Acid in Pure Solvents and Its Mixtures. J. Chem. Eng. Data 2009, 54, 64–68. 10.1021/je8005979.

[ref148] Domańska U. Solid-liquid phase relations of some normal long-chain fatty acids in selected organic one- and two-component solvents. Ind. Eng. Chem. Res. 1987, 26, 1153–1162. 10.1021/ie00066a016.

[ref149] Calvo B.; Cepeda E. A. Solubilities of Stearic Acid in Organic Solvents and in Azeotropic Solvent Mixtures. J. Chem. Eng. Data 2008, 53, 628–633. 10.1021/je7006567.

[ref150] Prausnitz J. M.; Lichtenthaler R. N.; De Azevedo E. G. Molecular Thermodynamics of Fluid-Phase Equilibria; Pearson Education, 1998.

[ref151] Febra S. A. Ring formation in a statistical associating fluid theory framework. Ph.D. thesis, Imperial College London, 2018.

[ref152] Chua Y. Z.; Do H. T.; Schick C.; Zaitsau D.; Held C. New experimental melting properties as access for predicting amino-acid solubility. RSC Adv. 2018, 8, 6365–6372. 10.1039/C8RA00334C. 35540399 PMC9078280

[ref153] Do H. T.; Chua Y. Z.; Habicht J.; Klinksiek M.; Hallermann M.; Zaitsau D.; Schick C.; Held C. Melting properties of peptides and their solubility in water. Part 1: dipeptides based on glycine or alanine. RSC Adv. 2019, 9, 32722–32734. 10.1039/C9RA05730G. 35529741 PMC9073158

[ref154] Wesolowski M.; Konarski T. General remarks on the thermal decomposition of some drugs. J. Therm. Anal. Calorim. 1995, 43, 279–289. 10.1007/BF02635995.

[ref155] An M.; Qiu J.; Yi D.; Liu H.; Hu S.; Han J.; Huang H.; He H.; Liu C.; Zhao Z.; Shi Y.; Wang P. Measurement and Correlation for Solubility of L-Alanine in Pure and Binary Solvents at Temperatures from 283.15 to 323.15 K. J. Chem. Eng. Data 2020, 65, 549–560. 10.1021/acs.jced.9b00743.

[ref156] Abraham M. H.; Grellier P. L. Substitution at saturated carbon. Part XIX. The effect of alcohols and water on the free energy of solutes and on the free energy of transition states in SN and SE reactions. J. Chem. Soc., Perkin Trans. 1975, 2, 1856–1863. 10.1039/p29750001856.

[ref157] Bowden N. A.; Sanders J. P. M.; Bruins M. E. Solubility of the proteinogenic α-amino acids in water, ethanol, and ethanol-water mixtures. J. Chem. Eng. Data 2018, 63, 488–497. 10.1021/acs.jced.7b00486. 29545650 PMC5846082

[ref158] Cao Z.; Hu Y.; Li J.; Kai Y.; Yang W. Solubility of glycine in binary system of ethanol+ water solvent mixtures: Experimental data and thermodynamic modeling. Fluid Ph. Equilib. 2013, 360, 156–160. 10.1016/j.fluid.2013.09.013.

[ref159] Dey B. P.; Lahiri S. C. Solubilities of Amino Acids in Ethanol + Water Mixture at Different Temperatures. J. Indian Chem. Soc. 1992, 69, 552–557.

[ref160] Long B.-W.; Wang L.-S.; Wu J.-S. Solubilities of 1, 3-benzenedicarboxylic acid in water + acetic acid solutions. J. Chem. Eng. Data 2005, 50, 136–137. 10.1021/je049784c.

[ref161] Pucher G.; Dehn W. M. Solubilities in mixtures of two solvents. J. Am. Chem. Soc. 1921, 43, 1753–1758. 10.1021/ja01441a002.

[ref162] Fan Y.; Zhu W.; Hu Y.; Yang W.; Xu Q.; Liu X.; Heng B. The Research and Measurement about the Solubility of L-Serine in Eight Common Pure Solvents and Four Binary Mixed Solvents for T = (278.15–333.15) K. J. Chem. Eng. Data 2019, 64, 4398–4411. 10.1021/acs.jced.9b00460.

[ref163] Zhang C.; Liu B.; Wang X.; Wang H.; Zhang H. Measurement and Correlation of Solubility of L-Valine in Water + (Ethanol, N, N-Dimethylformamide, Acetone, Isopropyl Alcohol) from 293.15 to 343.15 K. J. Chem. Eng. Data 2014, 59, 2732–2740. 10.1021/je500255d.

[ref164] McMeekin T. L.; Cohn E. J.; Weare J. H. Studies in the physical chemistry of amino acids, peptides and related substances. VII. A comparison of the solubility of amino acids, peptides and their derivatives. J. Am. Chem. Soc. 1936, 58, 2173–2181. 10.1021/ja01302a026.

[ref165] Bouchard A.; Hofland G. W.; Witkamp G.-J. Solubility of glycine polymorphs and recrystallization of β-glycine. J. Chem. Eng. Data 2007, 52, 1626–1629. 10.1021/je700014k.

[ref166] Talukdar H.; Rudra S.; Kundu K. K. Thermodynamics of transfer of glycine, diglycine, and triglycine from water to aqueous solutions of urea, glycerol, and sodium nitrate. Can. J. Chem. 1988, 66, 461–468. 10.1139/v88-080.

[ref167] Needham T. E. Jr; Paruta A. N.; Gerraughty R. J. Solubility of amino acids in mixed solvent systems. J. Pharm. Sci. 1971, 60, 258–260. 10.1002/jps.2600600221. 5572449

[ref168] Tseng H.-C.; Lee C.-Y.; Weng W.-L.; Shiah I.-M. Solubilities of amino acids in water at various pH values under 298.15 K. Fluid Ph. Equilib 2009, 285, 90–95. 10.1016/j.fluid.2009.07.017.

[ref169] Rashin A. A.; Honig B. Reevaluation of the Born model of ion hydration. J. Phys. Chem. 1985, 89, 5588–5593. 10.1021/j100272a006.

[ref170] Sandler S. I. Chemical, Biochemical, and Engineering Thermodynamics, 4th ed.; Wiley, 2005.

[ref171] Schick D.; Bierhaus L.; Strangmann A.; Figiel P.; Sadowski G.; Held C. Predicting CO_2_ solubility in aqueous and organic electrolyte solutions with ePC-SAFT advanced. Fluid Phase Equilib. 2023, 567, 113714 10.1016/j.fluid.2022.113714.

[ref172] Harned H. S., Owen B. B. The Physical Chemistry of Electrolyte Solutions; Reinhold, 1958; pp 634–643.

[ref173] Covington A. K.; Ferra M. I. A.; Robinson R. A. Ionic product and enthalpy of ionization of water from electromotive force measurements. J. Chem. Soc., Faraday Trans. 1 1977, 73, 1721–1730. 10.1039/f19777301721.

[ref174] Branch G. E. K.; Miyamoto S. Dissociation constants and heats of ionization of some simple amino acids and peptides. J. Am. Chem. Soc. 1930, 52, 863–868. 10.1021/ja01366a002.

