# Supplementary material for: Predicting the Solubility of Amino Acids and Peptides with the SAFT-γ Mie Approach: Neutral and Charged Models
Source: Ind Eng Chem Res. 2024 Nov 11;63(46):20397–423. doi: 10.1021/acs.iecr.4c02995 (PMC11583215; doi:10.1021/acs.iecr.4c02995)
Supplement: Supplementary file 1 — ie4c02995_si_002.pdf [file ie4c02995_si_002.pdf]

Supporting Information to: On predicting the solubility of amino acids and peptides with the SAFT-γ Mie approach: neutral and charged models

Ahmed Alyazidi,† Shubhani Paliwal,† Felipe A. Perdomo,†,‡ Amy Mead,† Mingxia Guo,† Jerry Heng,† Thomas Bernet,† Andrew Haslam,† Claire S. Adjiman,† George Jackson,† and Amparo Galindo∗∗,†

†*Department of Chemical Engineering, Institute for Molecular Science and Engineering, and Sargent Centre for Process Systems Engineering, Imperial College London, South Kensington Campus, London, SW7 2AZ, UK*

‡*Current address: School of Engineering, The University of Edinburgh, Sanderson Building, Robert Stevenson Road, The King's Buildings, Edinburgh, EH9 3FB, UK*

E-mail: a.galindo@imperial.ac.uk

Electrostatic contributions to the SAFT-γ Mie Helmholtz free energy: Model M

We describe here the treatment of ionic molecules when adopting the Molecular Model (M), as described in Figure 1 of the article. The derivation, which is largely analogous to that relating to Model G, follows a similar path beginning with the ion term from the

SAFT-γ Mie free energy (equation 10, reproduced here as equation (1)):

$$\frac{A^{\text{ion}}}{Nk_{\text{B}}T} = \frac{U^{\text{MSA}}}{Nk_{\text{B}}T} + \frac{\Gamma^3}{3\pi\rho}, \tag{1}$$

where U^{MSA} is the MSA contribution to the internal energy U, $\rho = N/V$ is the number density of molecules in the system, V is the system volume, and Γ is the screening length of the electrostatic forces. Considering spherical ions, U^{MSA} is given by

$$\frac{U^{\text{MSA}}}{Nk_{\text{B}}T} = -\frac{e^2}{(4\pi\epsilon_0)\rho k_{\text{B}}TD}\left[\Gamma\rho\sum_{i\in I}\left(\frac{x_i Z_i^2}{1+\Gamma\sigma_{ii}}\right) + \frac{\pi}{2\Delta}\Omega P_n^2\right], \tag{2}$$

where I is the set of ionic species in the mixture and

$$\Delta = 1 - \frac{\pi\rho}{6}\sum_{i\in I} x_i\sigma_{ii}^3, \tag{3}$$

$$P_n = \frac{\rho}{\Omega}\sum_{i\in I}\frac{x_i\sigma_{ii}Z_i}{1+\Gamma\sigma_{ii}}, \tag{4}$$

and

$$\Omega = 1 + \frac{\pi\rho}{2\Delta}\sum_{i\in I}\frac{x_i\sigma_{ii}^3}{1+\Gamma\sigma_{ii}}. \tag{5}$$

Δ represents the packing fraction of the ions as a function of the diameters σ_{ii} $(i \in I)$. P_n and Ω are coupling parameters. The coupling parameter P_n relates to the charge of the ions, whereas Ω couples to the packing fraction of the ions. Both parameters are functions of the ionic diameters, as well as the screening length, Γ:

$$\Gamma^2 = \frac{\pi e^2\rho}{(4\pi\epsilon_0)\,Dk_{\text{B}}T}\sum_{i\in I} x_i Q_i^2, \tag{6}$$

where the effective charge $Q_i(\Gamma)$ is given by

$$Q_i = \frac{Z_i - \sigma_{ii}^2 P_n\left(\pi/\left(2\Delta\right)\right)}{1+\Gamma\sigma_{ii}}. \tag{7}$$

In adopting Model M one each non-spherical ion onto a single, large sphere of diameter

$$\tilde{\sigma}_{\mathrm{eff},\,ii}^{\mathrm{MSA,M}} = \left(\sum_{k=1}^{N_\mathrm{G}} \nu_{k,i} \nu_k^* S_k \sigma_{kk}^3 \right)^{1/3} \quad, \; i \in I \tag{8}$$

and σ_{ii} is replaced by this effective $\tilde{\sigma}_{\mathrm{eff},\,ii}^{\mathrm{MSA,M}}$ in equations (2) to (7).

Adopting the Born model, one assumes that a spherical cavity of diameter $\sigma_{ii}^{\mathrm{Born}}$ is created for each ion, i, (independently of any others) in the dielectric medium. This leads to a contribution to the Helmholtz free energy given by:

$$A^{\mathrm{Born}} = -\frac{e^2}{4\pi\epsilon_0} \left(1 - \frac{1}{D}\right) \sum_{i \in I} \frac{N_i Z_i^2}{\sigma_{ii}^{\mathrm{Born}}}. \tag{9}$$

The Born diameter $\tilde{\sigma}_{\mathrm{eff},\,ii}^{\mathrm{Born,\,M}}$ of the single large sphere used to map each non-spherical ion in model M is given by:

$$\tilde{\sigma}_{\mathrm{eff},\,ii}^{\mathrm{Born,\,M}} = \left(\sum_{k=1}^{N_{\mathrm{G},i}} \nu_{k,i} \nu_k^* S_k \left(\sigma_{kk}^{\mathrm{Born}}\right)^3 \right)^{1/3} \quad, \; i \in I$$

where $N_{\mathrm{G},i}$ represents the total number of groups making up the ionic molecule of species i, and $\nu_{k,i}$ is the number of groups of type k within the ionic molecule.

This larger diameter is then incorporated into the Born free-energy expression:

$$A^{\mathrm{Born,\,M}} = -\frac{e^2}{4\pi\epsilon_0} \left(1 - \frac{1}{D}\right) \sum_{i \in I} \frac{N_i Z_i^2}{\tilde{\sigma}_{\mathrm{eff},\,ii}^{\mathrm{Born,\,M}}}, \tag{10}$$

or, equivalently (in terms of mole fractions, and dividing through in customary fashion by $k_\mathrm{B}T$ to give a dimensionless free energy),

$$\frac{A^{\mathrm{Born,\,M}}}{N k_\mathrm{B} T} = -\frac{e^2}{4\pi\epsilon_0 k_\mathrm{B} T} \left(1 - \frac{1}{D}\right) \sum_{i \in I} \frac{x_i Z_i^2}{\tilde{\sigma}_{\mathrm{eff},\,ii}^{\mathrm{Born,\,M}}}\,. \tag{11}$$

The Born contribution is thereby reduced by a factor of $\sim \frac{\tilde{\sigma}^{\mathrm{Born,M}}_{\mathrm{eff},ii}}{\sigma^{\mathrm{Born}}_{ii}}$.

Expressions for the chemical potential and pressure due to the ion contribution of Model G

The algebra involved in taking the derivatives of A^{ion} to obtain the chemical potential, $\mu_i^{\mathrm{MSA,G}}$, and pressure, $p^{\mathrm{MSA,G}}$, is cumbersome, comprising multiple applications of the chain rule of calculus, although the procedure is simplified a little by recognising that the derivatives of A^{ion} with respect to Γ are zero (see "Derivatives of the Helmholtz free energy with respect to screening length Γ"). The chemical potential of component i is thus given by

$$
\begin{aligned}
\frac{\mu_i^{\mathrm{ion,G}}}{k_{\mathrm{B}}T} = \frac{1}{k_{\mathrm{B}}T}\left(\frac{\partial A^{\mathrm{ion,G}}}{\partial N_i}\right)_{V,T,N_{j\neq i}} &= -\frac{e^2 V}{4\pi\epsilon_0 D k_{\mathrm{B}}T}\left\{\frac{\Gamma}{V}\sum_{k=1;Z_k\neq 0}^{N_{\mathrm{G}}}\frac{\nu_{k,i}Z_k^2}{\left(1+\Gamma\tilde{\sigma}^{\mathrm{MSA,G}}_{\mathrm{eff},kk}\right)}\right.\\
&+ \left(\frac{\partial A^{\mathrm{MSA,G}}}{\partial \Delta}\right)_{V,T,N_{j\neq i}}\left(\frac{\partial \Delta}{\partial N_i}\right)_{V,T,N_{j\neq i}}\\
&+ \left(\frac{\partial A^{\mathrm{MSA,G}}}{\partial \Omega}\right)_{V,T,N_{j\neq i}}\left(\frac{\partial \Omega}{\partial N_i}\right)_{V,T,N_{j\neq i}}\\
&+ \left.\left(\frac{\partial A^{\mathrm{MSA,G}}}{\partial P_n}\right)_{V,T,N_{j\neq i}}\left(\frac{\partial P_n}{\partial N_i}\right)_{V,T,N_{j\neq i}}\right\}\\
&- \frac{U^{\mathrm{MSA,G}}N}{D k_{\mathrm{B}}T}\left(\frac{\partial D}{\partial N_i}\right)_{V,T,N_{j\neq i}}.
\end{aligned} \tag{12}
$$

The individual partial derivatives appearing in equation (12) are obtained as follows:

$$
\left(\frac{\partial A^{\mathrm{MSA,G}}}{\partial \Delta}\right)_{V,T,N_{j\neq i}} = -\frac{\pi\Omega}{2}\left(\frac{P_n}{\Delta}\right)^2; \tag{13}
$$

$$
\left(\frac{\partial \Delta}{\partial N_i}\right)_{V,T,N_{j\neq i}} = \frac{-\pi}{6V}\sum_{k=1;Z_k\neq 0}^{N_{\mathrm{G}}}\nu_{k,i}\left(\tilde{\sigma}^{\mathrm{MSA,G}}_{\mathrm{eff},kk}\right)^3; \tag{14}
$$

$$\left(\frac{\partial A^{\mathrm{MSA,\,G}}}{\partial \Omega}\right)_{V,T,N_{j\neq i}} = \frac{\pi P_n^2}{2\Delta}\,; \tag{15}$$

$$\left(\frac{\partial \Omega}{\partial N_i}\right)_{V,T,N_{j\neq i}} = \frac{\pi}{2\Delta V}\sum_{k=1;Z_k\neq 0}^{N_{\mathrm{G}}}\frac{\nu_{k,i}Z_k^2}{\left(1+\Gamma\tilde{\sigma}^{\mathrm{MSA,G}}_{\mathrm{eff},\,kk}\right)} + \frac{(1-\Omega)}{\Delta}\left(\frac{\partial \Delta}{\partial N_i}\right)_{V,T,N_{j\neq i}}\,; \tag{16}$$

$$\left(\frac{\partial A^{\mathrm{MSA,\,G}}}{\partial P_n}\right)_{V,T,N_{j\neq i}} = \frac{\pi \Omega P_n}{\Delta}\,; \tag{17}$$

$$\left(\frac{\partial P_n}{\partial N_i}\right)_{V,T,N_{j\neq i}} = \frac{1}{\Omega V}\sum_{k=1;Z_k\neq 0}^{N_{\mathrm{G}}}\frac{\nu_{k,i}\tilde{\sigma}^{\mathrm{MSA,\,G}}_{\mathrm{eff},\,kk}Z_k}{\left(1+\Gamma\tilde{\sigma}^{\mathrm{MSA,G}}_{\mathrm{eff},\,kk}\right)} - \frac{P_n}{\Omega}\left(\frac{\partial \Omega}{\partial N_i}\right)_{V,T,N_{j\neq i}}\,. \tag{18}$$

The pressure is derived from equations 10 and 11 of the article, once again involving multiple applications of the chain rule. The volume derivatives of Δ, Ω and P_n are first required:

$$\left(\frac{\partial \Delta}{\partial V}\right)_{T,N} = \frac{\pi}{6V^2}\sum_{i=1;Z_i\neq 0}^{N_{\mathrm{C}}}\sum_{k=1;Z_k\neq 0}^{N_{\mathrm{G}}} x_i\nu_{k,i}\left(\tilde{\sigma}^{\mathrm{MSA,G}}_{\mathrm{eff},kk}\right)^3 = \frac{1-\Delta}{V}\,; \tag{19}$$

$$\begin{aligned}\left(\frac{\partial \Omega}{\partial V}\right)_{T,N} &= -\left(\frac{\pi}{2V^2\Delta}+\frac{\pi}{2V\Delta^2}\left(\frac{\partial \Delta}{\partial V}\right)\right)\sum_{i=1;Z_i\neq 0}^{N_{\mathrm{C}}}\sum_{k=1;Z_k\neq 0}^{N_{\mathrm{G}}}\frac{x_i\nu_{k,i}\left(\tilde{\sigma}^{\mathrm{MSA,G}}_{\mathrm{eff},kk}\right)^3}{1+\Gamma\tilde{\sigma}^{\mathrm{MSA,G}}_{\mathrm{eff},kk}}\\ &= \frac{1-\Omega}{V}+\left(\frac{1-\Omega}{\Delta}\right)\left(\frac{1-\Delta}{V}\right) = \frac{1-\Omega}{V\Delta}\,;\end{aligned} \tag{20}$$

$$\begin{aligned}\left(\frac{\partial P_n}{\partial V}\right)_{T,N} &= -\left(\frac{1}{V^2\Omega}+\frac{1}{V\Omega^2}\left(\frac{\partial \Omega}{\partial V}\right)\right)\sum_{i=1;Z_i\neq 0}^{N_{\mathrm{C}}}\sum_{k=1;Z_k\neq 0}^{N_{\mathrm{G}}}\frac{x_i\nu_{k,i}\tilde{\sigma}^{\mathrm{MSA,G}}_{\mathrm{eff},kk}Z_k}{1+\Gamma\tilde{\sigma}^{\mathrm{MSA,G}}_{\mathrm{eff},kk}}\\ &= -\frac{P_n}{V}-\frac{P_n}{\Omega}\left(\frac{1-\Omega}{V\Delta}\right)\,.\end{aligned} \tag{21}$$

For the purposes of the volume derivative, the final term in equation 11 of the article can be thought of as a constant $\times$ $V\Omega P_n^2/\Delta$; the relevant derivative is thus obtained via

$$\begin{aligned}\frac{\partial}{\partial V}\left(\frac{V\Omega P_n^2}{\Delta}\right)_{T,N} &= \frac{\Omega P_n^2}{\Delta} + V\left\{\Omega\frac{2P_n}{\Delta}\left(\frac{\partial P_n}{\partial V}\right) - \frac{P_n^2}{\Delta^2}\left(\frac{\partial \Delta}{\partial V}\right) + \frac{P_n^2}{\Delta}\left(\frac{\partial \Omega}{\partial V}\right)\right\} \\ &= \ldots = \left(\frac{P_n}{\Delta}\right)^2 \left(\Omega\Delta - 2\Omega\Delta - 2(1-\Omega) - \Omega(1-\Delta) + (1-\Omega)\right) \\ &= -\left(\frac{P_n}{\Delta}\right)^2 . \end{aligned} \tag{22}$$

One finally obtains

$$\begin{aligned} p^{\mathrm{ion,G}} &= \left(-\frac{\partial A^{\mathrm{ion,G}}}{\partial V}\right)_{N,T} \\ &= -\frac{\Gamma^3 k_{\mathrm{B}} T}{3\pi} - \frac{e^2}{8\epsilon_0 D}\left(\frac{P_n}{\Delta}\right)^2 + \frac{U^{\mathrm{MSA,G}}}{D}\left(\frac{\partial D}{\partial V}\right)_{T,N} . \end{aligned} \tag{23}$$

Derivatives of the Helmholtz free energy with respect to screening length Γ

(This demonstration is adapted from the PhD thesis of Schreckenberg [1].)

Derived thermodynamic properties in the case of the MSA (in the primitive model) are obtained by taking the derivative of the Helmholtz free energy with respect to the appropriate state variable, such as volume, number of components or temperature. Applying the chain rule, the first derivative with respect to any state variable Ψ is obtained as

$$\begin{aligned}\frac{\partial A}{\partial \Psi} &= \left(\frac{\partial A}{\partial \Psi}\right)_{\Gamma,\Delta,\Omega,P_n} + \left(\frac{\partial A}{\partial \Gamma}\right)_{\Psi,\Delta,\Omega,P_n}\left(\frac{\partial \Gamma}{\partial \Psi}\right) + \left(\frac{\partial A}{\partial \Delta}\right)_{\Psi,\Gamma}\left(\frac{\partial \Delta}{\partial \Psi}\right) \\ &+ \left(\frac{\partial A}{\partial \Omega}\right)_{\Psi,\Gamma}\left(\frac{\partial \Omega}{\partial \Psi}\right) + \left(\frac{\partial A}{\partial P_n}\right)_{\Psi,\Gamma}\left(\frac{\partial P_n}{\partial \Psi}\right) . \end{aligned} \tag{24}$$

We provide first the demonstration for the simpler case of Model M. Taking the derivative with respect to Γ in the second term on the right-hand side of equation (24), as applied

to $A^{\rm ion}$ given by equations (1) and (2) for Model M leads to

$$\frac{\partial A^{\rm ion}}{\partial \Gamma} = -\frac{Ve^2}{4\pi D\epsilon_0}\left[\frac{1}{V}\sum_{i\in I}\frac{N_i Z_i^2}{1+\Gamma\sigma_{ii}}\left(1-\frac{\Gamma\sigma_{ii}}{1+\Gamma\sigma_{ii}}\right)+\frac{\pi P_n}{2\Delta}\left(P_n\frac{\partial\Omega}{\partial\Gamma}+2\Omega\frac{\partial P_n}{\partial\Gamma}\right)\right]+\frac{VkT\Gamma^2}{\pi}\,. \tag{25}$$

From equation (4) we have

$$\begin{aligned}\frac{\partial P_n}{\partial \Gamma} &= \frac{-1}{\Omega^2 V}\frac{\partial\Omega}{\partial\Gamma}\sum_{i\in I}\frac{N_i\sigma_{ii}Z_i}{1+\Gamma\sigma_{ii}}-\frac{1}{\Omega V}\sum_{i\in I}\frac{N_i\sigma_{ii}^2 Z_i}{(1+\Gamma\sigma_{ii})^2}\\ &= \frac{-P_n}{\Omega}\frac{\partial\Omega}{\partial\Gamma}-\frac{1}{\Omega V}\sum_{i\in I}\frac{N_i\sigma_{ii}^2 Z_i}{(1+\Gamma\sigma_{ii})^2}\,,\end{aligned} \tag{26}$$

whence

$$\begin{aligned}\frac{\partial A^{\rm ion}}{\partial \Gamma} &= -\frac{Ve^2}{4\pi D\epsilon_0}\left[\frac{1}{V}\sum_{i\in I}\frac{N_i Z_i^2}{(1+\Gamma\sigma_{ii})^2}+\frac{\pi P_n}{2\Delta}\left(P_n\frac{\partial\Omega}{\partial\Gamma}-\frac{2}{V}\sum_{i\in I}\frac{N_i\sigma_{ii}^2 Z_i}{(1+\Gamma\sigma_{ii})^2}-2P_n\frac{\partial\Omega}{\partial\Gamma}\right)\right]\\ &+ \frac{VkT\Gamma^2}{\pi}\,.\end{aligned} \tag{27}$$

Equation (5) provides

$$\frac{\partial\Omega}{\partial\Gamma} = \frac{-\pi}{2\Omega V}\sum_{i\in I}\frac{N_i\sigma_{ii}^4}{(1+\Gamma\sigma_{ii})^2}\,, \tag{28}$$

allowing cancellation of V from the numerator of the prefactor in equation (33) and leading to

$$\begin{aligned}\frac{\partial A^{\rm ion}}{\partial \Gamma} &= -\frac{e^2}{4\pi D\epsilon_0}\left[\sum_{i\in I}\frac{N_i Z_i^2}{(1+\Gamma\sigma_{ii})^2}-\frac{\pi P_n}{\Delta}\sum_{i\in I}\frac{N_i\sigma_{ii}^2 Z_i}{(1+\Gamma\sigma_{ii})^2}\right.\\ &+ \left.\frac{\pi^2 P_n^2}{4\Delta^2}\sum_{i\in I}\frac{N_i\sigma_{ii}^4}{(1+\Gamma\sigma_{ii})^2}\right]+\frac{VkT\Gamma^2}{\pi}\,.\end{aligned} \tag{29}$$

Recalling equation (7) for Q_i, the terms in square brackets in equation (35) can be recognised as $\sum_{i\in I} N_i Q_i^2$, whereby this expression reduces to

$$\frac{\partial A^{\rm ion}}{\partial \Gamma} = -\frac{e^2}{4\pi D\epsilon_0}\sum_{i\in I} N_i Q_i^2+\frac{VkT\Gamma^2}{\pi} = 0\,. \tag{30}$$

The final equality follows upon substitution for Γ^2 using equation (6).

In the case of Model G, taking the derivative with respect to Γ in the second term on the right-hand side of equation (24), as applied to A^{ion} given by equations 10 and 11 of the article leads to

$$\begin{aligned}\frac{\partial A^{\mathrm{ion}}}{\partial \Gamma} &= -\frac{Ve^2}{4\pi D\epsilon_0}\left[\frac{1}{V}\sum_{i=1;Z_i\neq 0}^{N_\mathrm{C}}\sum_{k=1;Z_k\neq 0}^{N_\mathrm{G}}\frac{N_i\nu_{k,i}Z_k^2}{1+\Gamma\tilde{\sigma}^{\mathrm{MSA,G}}_{\mathrm{eff},kk}}\left(1-\frac{\Gamma\tilde{\sigma}^{\mathrm{MSA,G}}_{\mathrm{eff},kk}}{1+\Gamma\tilde{\sigma}^{\mathrm{MSA,G}}_{\mathrm{eff},kk}}\right)\right.\\ &\left.\quad+\frac{\pi P_n}{2\Delta}\left(P_n\frac{\partial\Omega}{\partial\Gamma}+2\Omega\frac{\partial P_n}{\partial\Gamma}\right)\right]+\frac{VkT\Gamma^2}{\pi}. \end{aligned} \tag{31}$$

From equation 14 of the article we have

$$\begin{aligned}\frac{\partial P_n}{\partial\Gamma} &= \frac{-1}{\Omega^2 V}\frac{\partial\Omega}{\partial\Gamma}\sum_{i=1;Z_i\neq 0}^{N_\mathrm{C}}\sum_{k=1;Z_k\neq 0}^{N_\mathrm{G}}\frac{N_i\nu_{k,i}\tilde{\sigma}^{\mathrm{MSA,G}}_{\mathrm{eff},kk}Z_k}{1+\Gamma\tilde{\sigma}^{\mathrm{MSA,G}}_{\mathrm{eff},kk}}\\ &\quad-\frac{1}{\Omega V}\sum_{i=1;Z_i\neq 0}^{N_\mathrm{C}}\sum_{k=1;Z_k\neq 0}^{N_\mathrm{G}}\frac{N_i\nu_{k,i}\left(\tilde{\sigma}^{\mathrm{MSA,G}}_{\mathrm{eff},kk}\right)^2 Z_k}{\left(1+\Gamma\tilde{\sigma}^{\mathrm{MSA,G}}_{\mathrm{eff},kk}\right)^2}\\ &= \frac{-P_n}{\Omega}\frac{\partial\Omega}{\partial\Gamma}-\frac{1}{\Omega V}\sum_{i=1;Z_i\neq 0}^{N_\mathrm{C}}\sum_{k=1;Z_k\neq 0}^{N_\mathrm{G}}\frac{N_i\nu_{k,i}\left(\tilde{\sigma}^{\mathrm{MSA,G}}_{\mathrm{eff},kk}\right)^2 Z_k}{\left(1+\Gamma\tilde{\sigma}^{\mathrm{MSA,G}}_{\mathrm{eff},kk}\right)^2}, \end{aligned} \tag{32}$$

whence

$$\begin{aligned}\frac{\partial A^{\mathrm{ion}}}{\partial \Gamma} &= -\frac{Ve^2}{4\pi D\epsilon_0}\left[\frac{1}{V}\sum_{i=1;Z_i\neq 0}^{N_\mathrm{C}}\sum_{k=1;Z_k\neq 0}^{N_\mathrm{G}}\frac{N_i\nu_{k,i}Z_k^2}{\left(1+\Gamma\tilde{\sigma}^{\mathrm{MSA,G}}_{\mathrm{eff},kk}\right)^2}\right.\\ &\left.\quad+\frac{\pi P_n}{2\Delta}\left(P_n\frac{\partial\Omega}{\partial\Gamma}-\frac{2}{V}\sum_{i=1;Z_i\neq 0}^{N_\mathrm{C}}\sum_{k=1;Z_k\neq 0}^{N_\mathrm{G}}\frac{N_i\nu_{k,i}\left(\tilde{\sigma}^{\mathrm{MSA,G}}_{\mathrm{eff},kk}\right)^2 Z_k}{\left(1+\Gamma\tilde{\sigma}^{\mathrm{MSA,G}}_{\mathrm{eff},kk}\right)^2}-2P_n\frac{\partial\Omega}{\partial\Gamma}\right)\right]\\ &\quad+\frac{VkT\Gamma^2}{\pi}. \end{aligned} \tag{33}$$

Equation 15 of the article provides

$$\frac{\partial \Omega}{\partial \Gamma} = \frac{-\pi}{2\Omega V} \sum_{i=1;Z_i\neq 0}^{N_\mathrm{C}} \sum_{k=1;Z_k\neq 0}^{N_\mathrm{G}} \frac{N_i \nu_{k,i} \left(\tilde{\sigma}_{\mathrm{eff},kk}^{\mathrm{MSA,G}}\right)^4}{\left(1+\Gamma \tilde{\sigma}_{\mathrm{eff},kk}^{\mathrm{MSA,G}}\right)^2}, \quad (34)$$

allowing cancellation of V from the numerator of the prefactor in equation (33) and leading to

$$\begin{aligned}\frac{\partial A^{\mathrm{ion}}}{\partial \Gamma} \quad = \quad & -\frac{e^2}{4\pi D\epsilon_0}\left[\sum_{i=1;Z_i\neq 0}^{N_\mathrm{C}} \sum_{k=1;Z_k\neq 0}^{N_\mathrm{G}} \frac{N_i \nu_{k,i} Z_k^2}{\left(1+\Gamma \tilde{\sigma}_{\mathrm{eff},kk}^{\mathrm{MSA,G}}\right)^2}\right. \\ & -\frac{\pi P_n}{\Delta} \sum_{i=1;Z_i\neq 0}^{N_\mathrm{C}} \sum_{k=1;Z_k\neq 0}^{N_\mathrm{G}} \frac{N_i \nu_{k,i} \left(\tilde{\sigma}_{\mathrm{eff},kk}^{\mathrm{MSA,G}}\right)^2 Z_k}{\left(1+\Gamma \tilde{\sigma}_{\mathrm{eff},kk}^{\mathrm{MSA,G}}\right)^2} \\ & \left. +\frac{\pi^2 P_n^2}{4\Delta^2} \sum_{i=1;Z_i\neq 0}^{N_\mathrm{C}} \sum_{k=1;Z_k\neq 0}^{N_\mathrm{G}} \frac{N_i \nu_{k,i} \left(\tilde{\sigma}_{\mathrm{eff},kk}^{\mathrm{MSA,G}}\right)^4}{\left(1+\Gamma \tilde{\sigma}_{\mathrm{eff},kk}^{\mathrm{MSA,G}}\right)^2}\right] \\ & +\frac{VkT\Gamma^2}{\pi}. \end{aligned} \quad (35)$$

Recalling equation 17 of the article for Q_k, the terms in square brackets in equation (35) can be recognised as $\sum_{i=1;Z_i\neq 0}^{N_\mathrm{C}} \sum_{k=1;Z_k\neq 0}^{N_\mathrm{G}} N_i \nu_{k,i} Q_k^2$, whereby this expression reduces to

$$\frac{\partial A^{\mathrm{ion}}}{\partial \Gamma} = -\frac{e^2}{4\pi D\epsilon_0} \sum_{i=1;Z_i\neq 0}^{N_\mathrm{C}} \sum_{k=1;Z_k\neq 0}^{N_\mathrm{G}} N_i \nu_{k,i} Q_k^2 + \frac{VkT\Gamma^2}{\pi} = 0. \quad (36)$$

The final equality follows upon substitution for Γ^2 using equation 16 of the article.

References

(1) Schreckenberg, J. M. A. Modelling of the Thermodynamic and Solvation Properties of Electrolyte Solutions with the Statistical Associating Fluid Theory for Potentials of

Variable Range. Ph.D. thesis, Imperial College London, UK, 2011.
